# Multifunctional Donepezil Analogues as Cholinesterase and BACE1 Inhibitors

**DOI:** 10.3390/molecules23123252

**Published:** 2018-12-08

**Authors:** Keith D. Green, Marina Y. Fosso, Sylvie Garneau-Tsodikova

**Affiliations:** Department of Pharmaceutical Sciences, College of Pharmacy, University of Kentucky, Lexington, KY 40536-0596, USA; kgr234@uky.edu (K.D.G.); marina.fosso@uky.edu (M.Y.F.)

**Keywords:** Alzheimer’s disease, acetylcholinesterase, butyrylcholinesterase, β-secretase, inhibitors

## Abstract

A series of 22 donepezil analogues were synthesized through alkylation/benzylation and compared to donepezil and its 6-*O*-desmethyl adduct. All the compounds were found to be potent inhibitors of both acetylcholinesterase (AChE) and butyrylcholinesterase (BChE), two enzymes responsible for the hydrolysis of the neurotransmitter acetylcholine in Alzheimer’s disease patient brains. Many of them displayed lower inhibitory concentrations of *Ee*AChE (IC_50_ = 0.016 ± 0.001 µM to 0.23 ± 0.03 µM) and *Ef*BChE (IC_50_ = 0.11 ± 0.01 µM to 1.3 ± 0.2 µM) than donepezil. One of the better compounds was tested against *Hs*AChE and was found to be even more active than donepezil and inhibited *Hs*AChE better than *Ee*AChE. The analogues with the aromatic substituents were generally more potent than the ones with aliphatic substituents. Five of the analogues also inhibited the action of β-secretase (BACE1) enzyme.

## 1. Introduction

Alzheimer’s disease (AD) is a neurodegenerative disorder that is characterized by memory loss and cognitive deficits. It is the most common form of dementia among older adults and the sixth leading cause of death in the United States [1]. In 2018, the World Health Organization (WHO) reported that there have been more than 2 million deaths associated to AD and other dementias in 2016, and this number has doubled since 2000 [2]. In the United States alone, more than 5 million people are currently living with AD, and this number is expected to triple by 2050. Unfortunately, there is currently no cure for AD, which contributes to the deadly nature of this disease.

Despite all the research efforts invested, the specific cause(s) of AD remain(s) unclear [3]. Several molecular mechanisms of AD have been proposed, including the β-amyloid cascade, oxidative stress, metal imbalance, and cholinergic hypothesis [4]. The latter appears to be the most efficient therapeutic avenue in providing temporary relief of AD symptoms. Indeed, five drugs have been approved by the United States Food and Drug Administration (FDA) for the symptomatic treatment of AD, four of which are acetylcholinesterase (AChE) inhibitors: rivastigmine, galantamine, donepezil, and tacrine. These drugs prevent the action of cholinesterases (ChEs), which are responsible for the hydrolysis of the neurotransmitter acetylcholine (ACh), thereby increasing the levels of ACh in the brain and improving the cholinergic functions in AD patients [5,6]. In addition to AChE, another type of enzyme involved in the hydrolysis of the neurotransmitter ACh is butyrylcholinesterase (BChE). The activity and expression of BChE have been suggested to increase throughout the progression of AD, indicating that BChE may play an important role in the late stage of AD [7]. Therefore, inhibition of AChE and BChE remains a potential therapeutic target for AD treatment. However, targeting ChEs alone is definitely not sufficient.

Another hallmark of AD pathology is the accumulation of amyloid-β (Aβ) plaques on the brain [8]. These plaques are composed of Aβ peptides that result from the cleavage of the transmembrane amyloid precursor protein (APP) by secretases to form Aβ monomers that will aggregate to toxic fibrils [9]. β-secretase (BACE1) is an aspartyl protease that cleaves APP near the membrane surface, and it has been targeted for the development of potential therapies against AD [10].

Due to the multifactorial nature of AD, the development of compounds that could target different pathological features of the disease appears to be a viable research avenue. We previously reported on the synthesis and biological evaluation of a number of multifunctional molecules derived from tacrine and chalcones that are capable of targeting ChEs and Aβ [11,12,13,14,15]. Since donepezil is the most commonly prescribed medication for AD [4], and several other studies have focused on this drug to generate multifunctional compounds targeting various hallmarks of AD, including BACE1 [16,17,18,19], we decided to generate multi-targeted analogues derived from donepezil that would inhibit ChEs and β-secretase.

## 2. Results and Discussion

### 2.1. Chemistry

**Scheme 1:**
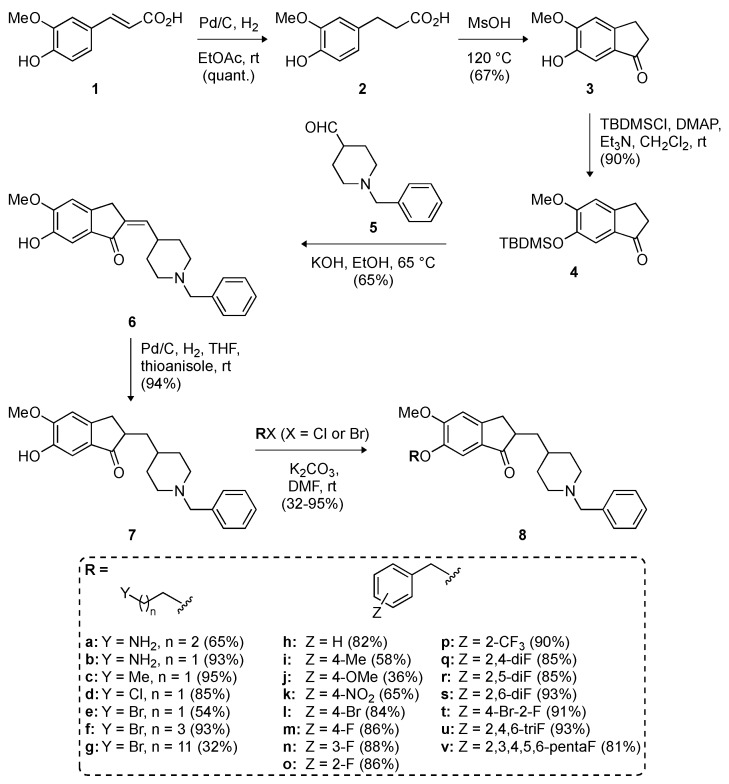
Synthetic scheme for the preparation of compounds **8a**–**v**.

The synthetic route utilized for the synthesis of donepezil analogues is outlined in Scheme 1. Starting from ferulic acid (**1**), hydrogenation in the presence of Pd/C, followed by cyclization in the presence of methanesulfonic acid (MsOH) produced ketone **3** with 67% yield [20,21]. Attempts to react the ketone **3** with the aldehyde **5** through aldol condensation were met with little success. To overcome this shortcoming, the free hydroxyl group in compound **3** was protected with a TBDMS group to yield the corresponding ketone **4** with 90% yield. This was then successfully condensed with the aldehyde **5** in the presence of KOH, to yield the α,β-unsaturated ketone **6** with 65% yield. Selective reduction of the double bond in the presence of a ketone and a benzyl group was achieved through a controlled poisoning of the palladium catalyst with thioanisole to give the 6-*O*-desmethyl donepezil adduct **7** with 94% yield. The latter bears a free hydroxyl group that was reacted with the corresponding alkyl or benzyl halides to yield 22 donepezil analogues (**8a**–**v**) with 32–95% yields.

### 2.2. Cholinesterase Inhibition

To evaluate the potential cholinesterase (ChE) inhibitory activity of donepezil, 6-*O*-desmethyl donepezil **7**, and the 22 newly synthesized donepezil analogues **8a**–**v**, their IC_50_ values were determined against AChE from *Electrophorus electricus* (*Ee*AChE) (Table 1 and Figures S82–S83) and *Ef*BChE from equine serum (*Equus ferus*) (Table 1 and Figures S84–S85) using the well-established Ellman method [22].

#### 2.2.1. AChE Inhibition

When comparing the 6-*O*-desmethyl donepezil **7** (R = H; IC_50_ = 0.41 ± 0.05 µM) with donepezil (R = Me; IC_50_ = 0.12 ± 0.01 µM) and its analogues **8a**–**f** and **8h**–**v** (R = various alkyl and benzylic groups; IC_50_ = 0.016 ± 0.001 µM to 0.23 ± 0.03 µM), it becomes evident that 6-*O*-alkylation/benzylation enhances *Ee*AChE inhibition, with the only exception being **8g** (IC_50_ = 0.79 ± 0.28 µM), which bears a hydrophobic 1-bromododecyl group. Other analogues were equal to or even better than donepezil at inhibiting the action of *Ee*AChE in vitro. With similar IC_50_ values, compound **8c** (R = *n*-propyl; IC_50_ = 0.14 ± 0.02 µM) was as potent as donepezil (R = Me; IC_50_ = 0.12 ± 0.01 µM). However, substituting the terminal methyl in the R group of compound **8c** (R = *n*-propyl; IC_50_ = 0.14 ± 0.02 µM) by a terminal amine in compound **8b** (R = H_2_NCH_2_CH_2_; IC_50_ = 0.021 ± 0.003 µM) drastically increased the potency. Indeed, a 6-fold reduction of the IC_50_ value of donepezil was observed. The amine group may form hydrogen bonds with Tyr70, Asp72, and Gln74 residues near the PAS [11]. Cancellation of this hydrogen bonding by replacing the terminal amine in the R group of compound **8b** (R = H_2_NCH_2_CH_2_; IC_50_ = 0.021 ± 0.003 µM) with a chlorine atom in **8d** (R = ClCH_2_CH_2_; IC_50_ = 0.059 ± 0.004 µM) or a bromine atom in **8e** (R = BrCH_2_CH_2_; IC_50_ = 0.044 ± 0.003 µM) resulted in an increase in the IC_50_ values, which only represented a 2- or 3-fold enhanced potency when compared to donepezil, respectively. Elongation of the R group also appeared to worsen the IC_50_ values. Indeed, adding an extra methylene to compound **8b** (R = H_2_NCH_2_CH_2_; IC_50_ = 0.021 ± 0.003 µM) gives compound **8a** (R = H_2_NCH_2_CH_2_CH_2_; IC_50_ = 0.054 ± 0.003 µM), while the addition of two methylene groups to compound **8e** (R = BrCH_2_CH_2_; IC_50_ = 0.044 ± 0.003 µM) gives compound **8f** (R = BrCH_2_CH_2_CH_2_CH_2_; IC_50_ = 0.061 ± 0.007 µM). Nevertheless, all these analogues remained better inhibitors of *Ee*AChE than donepezil.

Replacing the alkyl group in donepezil (R = Me; IC_50_ = 0.12 ± 0.01 µM) by an aromatic group in compound **8h** (R = Bn; IC_50_ = 0.13 ± 0.01 µM) did not affect the IC_50_ value. Likewise, additional substitutions at the *para*-position of the benzyl group resulted in IC_50_ values that were similar to that of donepezil. Indeed, compounds **8i** (R = 4-MeBn; IC_50_ = 0.23 ± 0.03 µM), **8j** (R = 4-OMeBn; IC_50_ = 0.13 ± 0.01 μM), **8k** (R = 4-NO_2_Bn; IC_50_ = 0.13 ± 0.02 μM), **8l** (R = 4-BrBn; IC_50_ = 0.071 ± 0.015 μM), and **8m** (R = 4-FBn; IC_50_ = 0.081 ± 0.005 μM) displayed IC_50_ values that were still within 1- to 2-fold of that of donepezil (R = Me; IC_50_ = 0.12 ± 0.01 μM). Similarly, when the fluoro group was moved from the *para*-position in compound **8m** (R = 4-FBn; IC_50_ = 0.081 ± 0.005 μM) to the *meta*- or *ortho*-positions in compounds **8n** (R = 3-FBn; IC_50_ = 0.16 ± 0.02 μM) and **8o** (R = 2-FBn; IC_50_ = 0.12 ± 0.02 μM), respectively, the potency of these analogues was comparable to donepezil (R = Me; IC_50_ = 0.12 ± 0.01 μM). However, replacing the fluoro group in **8o** (R = 2-FBn; IC_50_ = 0.12 ± 0.02 μM) by a CF_3_ group in **8p** (R = 2-CF_3_Bn; IC_50_ = 0.032 ± 0.010 μM) improved the IC_50_ by 4-fold. This may suggest that enhanced electron-withdrawing effect on the aromatic ring may improve the potency of the analogue. Attempts to spread out the electron-withdrawing effect throughout the aromatic ring led to compounds **8q** (R = 2,4-diF-Bn; IC_50_ = 0.11 ± 0.01 μM), **8r** (R = 2,5-diF-Bn; IC_50_ = 0.090 ± 0.009 μM), **8s** (R = 2,6-diF-Bn; IC_50_ = 0.016 ± 0.001 μM), and **8t** (R = 4-Br-2-F-Bn; IC_50_ = 0.054 ± 0.007 μM), with two electron-withdrawing groups, and compounds **8u** (R = 2,4,6-triF-Bn; IC_50_ = 0.027 ± 0.004 μM) and **8v** (R = 2,3,4,5-pentaF-Bn; IC_50_ = 0.17 ± 0.02 μM), with three and five electron-withdrawing groups, respectively. It thus appears that both *ortho*-positions on the benzyl group are very sensitive to the presence of electron-withdrawing groups, since **8s** (R = 2,6-diF-Bn; IC_50_ = 0.016 ± 0.001 μM) and **8u** (R = 2,4,6-triF-Bn; IC_50_ = 0.027 ± 0.004 μM) were eight and five times more potent than donepezil, respectively.

In order to confirm that the data obtained with *Ee*AChE would also apply to *Hs*AChE (from *Homo sapiens*), we tested donepezil along with a compound that displayed better inhibition than donepezil, **8t**. We found that both donepezil and compound **8t** inhibited *Hs*AChE better than the *Ee*AChE (Table 2). Compound **8t** (R = 4-Br-2-F-Bn; IC_50_ = 0.0018 ± 0.0006 μM) inhibited *Hs*AChE 18-fold better than donepezil (R = Me; IC_50_ = 0.032 ± 0.011 μM). In the case of *Ee*AChE, compound **8t** (R = 4-Br-2-F-Bn; IC_50_ = 0.054 ± 0.007 μM) had an IC_50_ value that was 2.2-fold better than donepezil (R = Me; IC_50_ = 0.12 ± 0.01 μM). These data would suggest that our inhibitors are well suited for working with *Hs*AChE.

#### 2.2.2. *Ef*BChE Inhibition

As expected, donepezil analogues **8a**–**v** were less effective against *Ef*BChE than *Ee*AChE. Indeed, donepezil is highly selective for *Ee*AChE over *Ef*BChE [23], and as a result, it is expected for its analogues to behave similarly. However, when compared to donepezil (R = Me; IC_50_ = 2.0 ± 0.1 μM), all but compounds **8c** (R = CH_3_CH_2_CH_2;_ IC_50_ = 2.1 ± 0.3 μM) and **8g** (IC_50_ = 5.2 ± 1.6 μM) appeared to be more effective at inhibiting the action of BChE. The presence of a terminal amine in compounds **8a** (R = H_2_NCH_2_CH_2_CH_2_; IC_50_ = 0.57 ± 0.04 μM) and **8b** (R = H_2_NCH_2_CH_2_; IC_50_ = 0.48 ± 0.03 μM) still drastically increased their potency by 4-fold when compared to donepezil. Substitution of the amine group in **8b** (R = H_2_NCH_2_CH_2_; IC_50_ = 0.48 ± 0.03 μM) with a chlorine atom in **8d** (R = ClCH_2_CH_2_; IC_50_ = 1.3 ± 0.1 μM) or a bromine atom in **8e** (R = BrCH_2_CH_2_; IC_50_ = 1.3 ± 0.2 μM) resulted again in an increase in the IC_50_ values, which only represented a 2-fold enhanced potency when compared to donepezil. Elongation of the R group did not have much effect, as the IC_50_ value of **8f** (R = BrCH_2_CH_2_CH_2_CH_2_; IC_50_ = 1.3 ± 0.2 μM) still remained within 2-fold that of donepezil.

A greater improvement of the IC_50_ values was more noticeable when the alkyl group in donepezil (R = Me; IC_50_ = 2.0 ± 0.1 μM) was replaced by an aromatic group. Compound **8h** (R = Bn; IC_50_ = 0.70 ± 0.05 μM) was 3-fold more potent than donepezil. Substitutions at the *para*-position of the benzyl group also contributed to reducing the IC_50_ values. Indeed, compounds **8i** (R = 4-Me-Bn; IC_50_ = 1.0 ± 0.2 μM), **8j** (R = 4-OMe-Bn; IC_50_ = 0.67 ± 0.17 μM), **8k** (R = 4-NO_2_Bn; IC_50_ = 0.46 ± 0.06 μM), **8l** (R = 4-Br-Bn; IC_50_ = 0.72 ± 0.10 μM), and **8m** (R = 4-F-Bn; IC_50_ = 0.57 ± 0.10 μM) displayed inhibitory efficacies of 2- to 4-fold better than donepezil (R = Me; IC_50_ = 2.0 ± 0.1 μM). Similarly, when the fluoro group was moved from the *para*-position in compound **8m** (R = 4-F-Bn; IC_50_ = 0.57 ± 0.10 μM) to the *meta*- or *ortho*-positions in compounds **8n** (R = 3-F-Bn; IC_50_ = 0.96 ± 0.15 μM) and **8o** (R = 2-F-Bn; IC_50_ = 0.76 ± 0.12 μM), respectively, the potency of these analogues was reduced by 1- to 2-fold. Replacing the fluoro group in **8o** (R = 2-F-Bn; IC_50_ = 0.76 ± 0.12 μM) by a CF_3_ group in **8p** (R = 2-CF_3_Bn; IC_50_ = 0.25 ± 0.08 μM) once again improved the IC_50_ by 8-fold. This is in agreement with the trend observed in *Ee*AChE inhibition. Indeed, the additional electron-withdrawing effect on the aromatic ring still appeared to increase the potency of the analogue. Compounds **8q** (R = 2,4-diF-Bn; IC_50_ = 0.48 ± 0.08 μM), **8r** (R = 2,5-diF-Bn; IC_50_ = 0.60 ± 0.15 μM), **8s** (R = 2,6-diF-Bn; IC_50_ = 0.44 ± 0.05 μM), and **8t** (R = 4-Br-2-F-Bn; IC_50_ = 0.37 ± 0.05 μM), with two electron-withdrawing groups, and compounds **8u** (R = 2,4,6-triF-Bn; IC_50_ = 0.20 ± 0.03 μM) and **8v** (R = 2,3,4,5-pentaF-Bn; IC_50_ = 0.11 ± 0.01 μM), with three and five electron-withdrawing groups, respectively, were all better *Ef*BChE inhibitors than donepezil (R = Me; IC_50_ = 2.0 ± 0.1 μM). Compound **8q** (R = 2,4-diF-Bn; IC_50_ = 0.48 ± 0.08 μM) was 4-fold better than donepezil, while **8r** (R = 2,5-diF-Bn; IC_50_ = 0.60 ± 0.15 μM) and **8s** (R = 2,6-diF-Bn; IC_50_ = 0.44 ± 0.05 μM) were 3- and 5-fold better, respectively. Replacing the fluorine atom at the *para*-position in **8q** (R = 2,4-diF-Bn; IC_50_ = 0.48 ± 0.08 μM) with a bromine atom in **8t** (R = 4-Br-2-F-Bn; IC_50_ = 0.37 ± 0.05 μM) did not impart a noticeable change. Compounds **8u** (R = 2,4,6-triF-Bn; IC_50_ = 0.20 ± 0.03 μM) and **8v** (R = 2,3,4,5-pentaF-Bn; IC_50_ = 0.11 ± 0.01 μM), with three and five electron-withdrawing groups, respectively, were 10- and 18-fold better than donepezil. The active site gorge of BChE is less constrained than that of AChE, allowing BChE to better accommodate bulky inhibitors [24]. This supports our observations that additional substitution on the aromatic ring increased the potency of the donepezil analogues against *Ef*BChE more than against *Ee*AChE.

We also calculated the selectivity index (SI) to understand the utility of the compounds. For all but one compound, **8v**, *Ee*AChE was inhibited 3.5- to 30-fold better than *Ef*BChE. Interestingly, compound **8v** was 1.5-fold more selective for *Ef*BChE. Clearly the donepezil analogues are better suited for inhibiting *Ee*AChE. We also looked at the selectivity of the inhibitors for *Ee*AChE *versus Hs*AChE. We observed that donepezil was 3-fold more selective for *Hs*AChE. Perhaps more interesting, compound **8t** was 30-fold more selective for *Hs*AChE over *Ee*AChE.

### 2.3. BACE1 Inhibition

It has previously been reported that donepezil has some BACE1 inhibitory activity [19]. Keeping this in mind we decided to test these compounds for BACE1 inhibitory activity (Table 3). Unlike with AChE and BChE, in general, donepezil analogues **8a**–**v** were not better than the parent donepezil at inhibiting the action of BACE1 in vitro, with the exception of **8c**, **8e**, **8f**, and **8l**, which were in the low micromolar range. Indeed, **8c** (R = CH_3_CH_2_CH_2;_ IC_50_ = 6.1 ± 0.1 μM), **8e** (R = BrCH_2_CH_2_; IC_50_ = 7.9 ± 0.9 μM), **8f** (R = BrCH_2_CH_2_CH_2_CH_2_; IC_50_ = 7.9 ± 2.4 μM), and **8l** (R = 4-Br-Bn; IC_50_ = 3.4 ± 0.1 μM) were within 5-fold of the IC_50_ values of donepezil. This suggests that our analogues are more selective in targeting the ChEs, but they do still target BACE1. As a control for the BACE1 inhibition assays, we used BACE inhibitor IV. Our inhibitors were poorer inhibitors than BACE inhibitor IV (IC_50_ = 0.63 ± 0.18 nM). While BACE inhibitor IV is better, it was designed to be very specific for that one target. However, with an illness such as Alzheimer’s disease, which has many facets and contributing factors, having multifunctional inhibitors that display activity against BACE1 and ChEs is beneficial.

### 2.4. BACE1 Modeling

**Figure 1:**
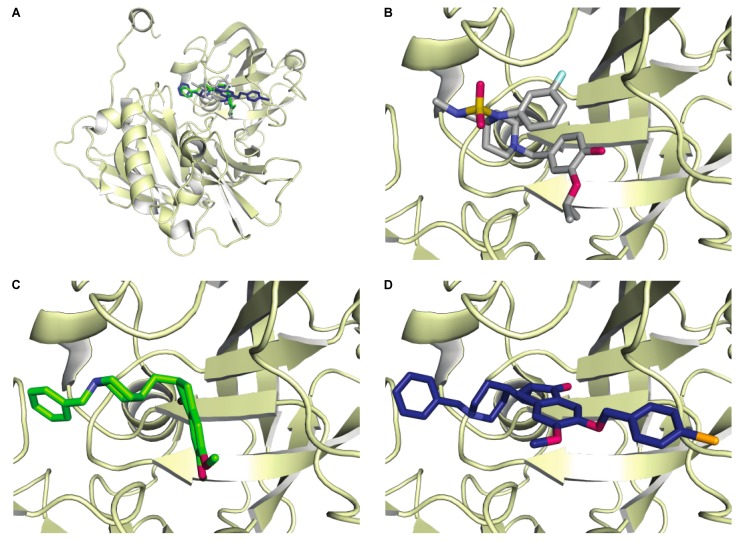
Molecular docking showing the overlay of donepezil (green) and compound **8l** (navy blue) with the known BACE1 inhibitor (gray) crystallized with BACE1 (PDB# 4FM7 [25]), shown as ribbons. Panel **A** shows the three compounds in the active site of BACE1. Panels **B**–**D** show the zoomed-in view of the known inhibitor (**B**), donepezil (**C**), and compound **8l** (**D**).

To aid in the understanding of donepezil and its analogues’ inhibitory activity of BACE1, we used SwissDock to perform some modeling studies. Figure 1 shows the crystal structure (PDB# 4FM7 [25], with a published inhibitor of BACE1 (published IC_50_ value = 0.1 μM). This inhibitor shares the vicinyl dioxygen-substituted phenyl ring found in donepezil. Based on the results of the modeling, the aromatic ring of donepezil aligns with that of the inhibitor originally co-crystallized with BACE1 (Figure 1A,B). When looking at the docking of donepezil (Figure 1C), it is apparent that is binds in a similar location to the reported co-crystallized inhibitor (Figure 1B), albeit not as tightly as apparent by the IC_50_ values, which are 10-fold different. When examining the docking of compound **8l** (Figure 1D), it is slightly twisted, likely due to the bulky 4-bromobenzyl substitution. This slight torsion could explain the roughly doubled IC_50_ value of compound **8l** when compared to that of donepezil. Based on the modeling, there is also room for more optimization at this location, reasoning that modifications of donepezil have the potential to yield better inhibitors than the parent compound if modified correctly.

## 3. Materials and Methods

### 3.1. General Information

All chemicals were purchased from Sigma Aldrich (St. Louis, MO, USA), Alfa Aesar (Ward Hill, MA, USA), and AK scientific (Union, CA, USA), and used without further purification. Chemical reactions were monitored by thin layer chromatography (TLC) using Merck (Darmstadt, Germany), Silica gel 60 F_250_ plates. Visualization was achieved using UV (model UVGL-58, UVP, Upland, CA, USA) light and a ceric molybdate stain (5 g (NH_4_)_2_Ce(NO_3_)_6_, 120 g (NH_4_)_6_Mo_7_O_24_ 4H_2_O, 80 mL H_2_SO_4_, 720 mL H_2_O). ^1^H and ^13^C-NMR spectra were recorded at 400 and 100 MHz, respectively, on a Varian 400 MHz spectrometer (Varian, Palo Alto, CA, USA), using the indicated deuterated solvents. Chemical shifts (δ) are given in parts per million (ppm). Coupling constants (J) are given in Hertz (Hz), and conventional abbreviations used for signal shape are as follows: s = singlet; d = doublet; t = triplet; m = multiplet; dd = doublet of doublets; ddd = doublet of doublet of doublets; br s = broad singlet; dt = doublet of triplets. High-resolution mass spectra were recorded on an AB SCIEX Triple TOF 5600 System (AB SCIEX, Framingham, MA, USA). The purity of the compound was further confirmed to be ≥95% by RP-HPLC (model 1260 Infinity, Agilent, Santa Clara, CA, USA) by using the following method: Flow rate = 0.5 mL/min; λ = 254 nm; column = Vydac 201SP^TM^ C18, 250 × 4.6 mm, 90A 5 μm; eluents: A = H_2_O + 0.1% TFA, B = MeCN; gradient profile: starting from 5% B, increasing from 5% to 100% B over 17 min, holding at 100% for 5 min, decreasing from 100% to 5% over 3 min. Prior to each injection, the HPLC column was equilibrated for 5 min with 5% B.

### 3.2. Synthesis of Compounds ***2***–***8v***

#### 3.2.1. 3-(4-Hydroxy-3-methoxyphenyl)propanoic acid (**2**)

A catalytic amount of 10% Pd/C (0.43 g) was added to a solution of ferulic acid (**1**, 6.0 g, 30.9 mmol) in degassed EtOAc (100 mL). The reaction flask was then sealed with a rubber septum and freed of air. The reaction mixture was stirred at room temperature (RT) overnight under H_2_ atmosphere. Upon completion, the reaction mixture was filtered through a bed of celite, and concentrated to afford the known compound **2** [26] (6.1 g, quant.) as an off-white solid: ^1^H-NMR (400 MHz, CDCl_3_, which matches the literature [26], Figure S1) δ 10.50 (very br s, 1H, CO_2_H), 6.82 (d, *J* = 7.6 Hz, 1H, aromatic), 6.69 (s, 1H, aromatic), 6.68 (d, *J* = 7.6 Hz, 1H, aromatic), 5.60 (very br s, 1H, OH), 3.85 (s, 3H, PhOCH_3_), 2.87 (t, *J* = 7.2 Hz, 2H, PhCH_2_CH_2_CO_2_H), 2.64 (t, *J* = 7.2 Hz, 2H, PhCH_2_CH_2_CO_2_H).

#### 3.2.2. 6-Hydroxy-5-methoxy-2,3-dihydroinden-1-one (**3**)

A solution of compound **2** (6.3 g, 32.1 mmol) in methanesulfonic acid (50 mL) was refluxed at 120 °C for 1 h. After cooling to RT, the reaction mixture was poured into ice-water, stirred for 5 min, and filtered to afford a crude dark brown solid, which was recrystallized from EtOH to afford the known compound **3** [20] (3.8 g, 67%) as a yellow solid: ^1^H-NMR (400 MHz, (CD_3_)_2_SO, which matches the lit. [20], Figure S2) δ 9.38 (s, 1H, OH), 7.03 (s, 1H, aromatic), 6.89 (s, 1H, aromatic), 3.83 (s, 3H, OCH_3_), 2.92 (t, *J* = 5.6 Hz, 2H, CH_2_CH_2_C=O), 2.49 (t, *J* = 5.6 Hz, 2H, CH_2_CH_2_C=O).

#### 3.2.3. 6-[*tert*-Butyl(dimethyl)silyl]oxy-5-methoxy-2,3-dihydroinden-1-one (**4**)

TBDMSCl (3.2 g, 21.3 mmol) was added to a solution of compound **3** (1.9 g, 10.7 mmol), DMAP (0.5 g, 4.3 mmol) and Et_3_N (3.0 mL, 21.3 mmol) in freshly distilled CH_2_Cl_2_ (100 mL). The reaction mixture was stirred at RT overnight before being quenched with H_2_O (100 mL). The organic layer was separated, washed with H_2_O (2 × 100 mL) and brine (100 mL), dried over anhydrous Mg_2_SO_4_, filtered, and concentrated under reduced pressure to afford a crude dark brown solid, which was purified by flash column chromatography (SiO_2_ gel, pure hexanes to hexanes:EtOAc/3:1, R_f_ 0.44 in Hexanes:EtOAc/3:1) to yield a brown solid, which was further triturated in hexanes to give compound **4** (2.8 g, 90%) as a white solid: ^1^H-NMR (400 MHz, CDCl_3_, Figure S3) δ 7.17 (s, 1H, aromatic), 6.84 (s, 1H, aromatic), 3.87 (s, 3H, PhOCH_3_), 3.02 (app. t, *J* = 5.6 Hz, 2H, CH_2_CH_2_C=O), 2.64 (app. t, *J* = 5.6 Hz, 2H, CH_2_CH_2_C=O), 0.98 (s, 9H, SiC(CH_3_)_3_), 0.14 (s, 6H, Si(CH_3_)_2_); ^13^C-NMR (100 MHz, CDCl_3_, Figure S4) δ 205.7 (C=O), 157.5 (C), 150.9 (C), 145.2 (C), 130.0 (C), 114.1 (CH), 107.8 (CH), 55.6 (CH_3_), 36.6 (CH_2_), 25.62 (CH_3_, three carbons), 25.56 (CH_2_), 18.4 (C), −4.7 (CH_3_, two carbons); *m*/*z* calcd. for C_16_H_25_O_3_Si^+^ [M + H]^+^ 293.1567; found 293.1563.

#### 3.2.4. (*E*)-2-[(1-Benzylpiperidin-4-yl)methylene]-6-hydroxy-5-methoxy-2,3-dihydroinden-1-one (**6**)

To a solution of compound **4** (1.00 g, 3.42 mmol) and *N*-benzylpiperidine-4-carboxaldehyde (**5**, 0.68 mL, 3.42 mmol) in EtOH (10 mL) was added KOH (0.5 g), and the mixture was refluxed at 65 °C. After 1 h, the reaction was analyzed by TLC (CH_2_Cl_2_:MeOH/19:1, R_f_ 0.30 in CH_2_Cl_2_:MeOH/19:1). The reaction mixture was concentrated under reduced pressure to give a crude yellow solid, which was re-dissolved in H_2_O (10 mL). 1 N aqueous HCl was then slowly added until pH 5 to yield a yellow precipitate, which was recrystallized in MeCN to afford compound **6** (0.81 g, 65%) as a yellow solid: ^1^H-NMR (400 MHz, CDCl_3_, Figure S5) δ 7.32–7.24 (m, 6H, aromatic), 6.87 (s, 1H, aromatic), 6.63 (d, *J* = 10.0 Hz, 1H, C=CH), 5.70 (br s, 1H, OH), 3.98 (s, 3H, OCH_3_), 3.56 (s, 2H), 3.51 (s, 2H), 2.91 (d, *J* = 11.6 Hz, 2H), 2.30 (m, 1H), 2.04 (t, *J* = 11.6 Hz, 2H), 1.70–1.60 (m, 4H); 13C-NMR (100 MHz, CDCl_3_, Figure S6) δ 192.6 (C=O), 152.6 (C), 145.8 (C), 143.4 (C), 139.9 (C), 138.2 (C), 135.5 (CH), 132.5 (CH), 129.2 (CH, two carbons), 128.2 (CH, two carbons), 127.0 (C), 108.7 (CH), 106.8 (CH), 63.5 (CH_2_), 56.2 (CH_3_), 53.1 (CH_2_, two carbons), 37.2 (CH_2_), 31.2 (CH_2_, two carbons), 29.5 (CH); *m*/*z* calcd. for C_23_H_26_NO_3_^+^ [M + H]^+^ 364.1907; found 364.1909.

#### 3.2.5. 2-[(1-Benzylpiperidin-4-yl)methyl]-6-hydroxy-5-methoxy-2,3-dihydroinden-1-one (**7**)

To a solution of compound **6** (101 mg, 0.28 mmol) in degassed THF (2.5 mL), 10% Pd/C was added (wet support, Sigma 520829-10G, 10 mg). The reaction flask was then sealed with a rubber septum and freed of air. Thioanisole (14.2 × 10^−7^ mL, obtained using 5 μL of a stock solution comprising 14.2 μL of thioanisole in 50 mL of anhydrous THF) was added, and the reaction mixture was stirred at RT overnight under H_2_ atmosphere. Upon completion, the reaction mixture was filtered through a bed of celite, and concentrated to yield the known compound **7** (96 mg, 94%) as a yellow solid: ^1^H-NMR (400 MHz, CDCl_3_, Figure S7) δ 7.30–7.20 (m, 6H, aromatic), 6.82 (s, 1H, aromatic), 3.96 (s, 3H, OCH_3_), 3.49 (s, 2H, NCH_2_Ph), 3.20 (dd, *J*_1_ = 18.0 Hz, *J*_2_ = 7.6 Hz, 1H), 2.87 (m, 2H), 2.66 (dt, *J*_1_ = 13.6 Hz, *J*_2_ = 3.6 Hz, 2H), 1.98–1.82 (m, 3H), 1.72–1.63 (m, 2H), 1.48 (m, 1H), 1.39–1.24 (m, 3H); 13C-NMR (100 MHz, CDCl_3_, Figure S8) δ 207.8 (C=O), 152.9 (C), 147.6 (C), 145.8 (C), 138.3 (C), 130.0 (C), 129.3 (CH, two carbons), 128.1 (CH, two carbons), 126.9 (CH), 108.1 (CH), 106.9 (CH), 63.4 (CH_2_), 56.2 (CH_3_), 53.7 (CH_2_, two carbons), 45.3 (CH), 38.7 (CH_2_), 34.4 (CH_2_), 33.4 (CH_2_), 32.9 (CH_2_), 31.7 (CH); *m/z* calcd. for C_23_H_28_NO_3_^+^ [M + H]^+^ 366.2064; found 366.2065. The purity of the compound was further confirmed by RP-HPLC: R_t_ = 17.17 min (96%; Figure S9).

#### 3.2.6. *tert*-Butyl *N*-(3-chloropropyl)carbamate (Boc-protected 3-chloropropylamine).

A solution of NaHCO_3_ (5.9 g, 70.8 mmol) in H_2_O (15 mL) was slowly added to a mixture of 3-chloropropylamine hydrochloride (1.0 g, 7.69 mmol), Boc_2_O (3.0 g, 13.8 mmol) and 1,4-dioxane (10 mL). The resulting mixture was stirred at 60 °C for 3 h. The reaction mixture was then diluted with H_2_O, and extracted with EtOAc (3×). The combined organic layers were washed with H_2_O (3×) and brine (3×), dried over anhydrous MgSO_4_, filtered, and concentrated under reduced pressure. The crude product obtained was purified by column chromatography (SiO_2_ gel, hexanes:EtOAc/5:1; R_f_ 0.31 in hexanes:EtOAc/5:1) to yield the known compound *tert*-butyl *N*-(3-chloropropyl)carbamate [27] (0.55 g, 36%) as a colorless oil: ^1^H-NMR (400 MHz, CDCl_3_, which matches the lit. [27], Figure S10) δ 4.65 (br s, 1H, NH), 3.56 (t, *J* = 6.4 Hz, 2H, ClCH_2_CH_2_), 3.26 (q, *J* = 6.4 Hz, 2H, CH_2_CH_2_NHBoc), 1.94 (p, *J* = 6.4 Hz, 2H, CH_2_CH_2_CH_2_), 1.42 (s, 9H, C(CH_3_)_3_).

#### 3.2.7. 2-[(1-Benzylpiperidin-4-yl)methyl]-6-[(3-*tert*-butyl-*N*-propylcarbamate)oxy]-5-methoxy-2,3-dihydroinden-1-one (Boc-protected compound **8a**)

A solution of compound **7** (215 mg, 0.59 mmol), *tert*-butyl *N*-(3-chloropropyl)carbamate (228 mg, 1.18 mmol), Cs_2_CO_3_ (575 mg, 1.76 mmol), and TBAI (109 mg, 0.29 mmol) in anhydrous DMF (5 mL) was stirred at RT overnight. The reaction mixture was then diluted with H_2_O, and extracted with EtOAc (3×). The combined organic layers were washed with H_2_O (3×) and brine (3×), dried over anhydrous MgSO_4_, filtered, and concentrated under reduced pressure. The crude product obtained was purified by column chromatography (SiO_2_ gel, pure CH_2_Cl_2_ to CH_2_Cl_2_:MeOH/19:1; R_f_ 0.55 in CH_2_Cl_2_:MeOH/9:1) to yield 2-[(1-benzylpiperidin-4-yl)methyl]-6-[(3-*tert*-butyl-*N*-propylcarbamate)oxy]-5-methoxy-2,3-dihydroinden-1-one (276 mg, 90%) as a white foam: ^1^H-NMR (400 MHz, CDCl_3_, Figure S11) δ 7.30–7.20 (m, 5H, aromatic), 7.11 (s, 1H, aromatic), 6.82 (s, 1H, aromatic), 5.52 (very br t, 1H, NH), 4.08 (t, *J* = 5.6 Hz, 2H, OCH_2_CH_2_), 3.93 (s, 3H, OCH_3_), 3.49 (s, 2H, NCH_2_Ph), 3.34 (q, *J* = 5.2 Hz, 2H, CH_2_CH_2_NHBoc), 3.20 (dd, *J*_1_ = 17.6 Hz, *J*_2_ = 8.0 Hz, 1H), 2.88 (m, 2H), 2.67 (m, 2H), 2.02–1.85 (m, 5H), 1.72–1.62 (m, 2H), 1.44 (s, 9H, C(CH_3_)_3_), 1.36–1.02 (m, 4H); 13C-NMR (100 MHz, CDCl_3_, Figure S12) δ 207.7 (C=O), 156.0 (CH), 155.6 (C=O), 148.9 (C), 148.4 (C), 138.3 (C), 129.2 (CH, two carbons), 128.1 (CH, three carbons), 126.9 (C), 107.4 (CH), 105.3 (CH), 78.9 (C), 68.3 (CH_2_), 63.4 (CH_2_), 56.0 (CH_3_), 53.74 (CH_2_), 53.72 (CH_2_), 45.4 (CH), 39.1 (CH_2_), 38.7 (CH_2_), 34.4 (CH_2_), 33.4 (CH_2_), 33.0 (CH_2_), 31.7 (CH), 29.0 (CH_2_), 28.5 (CH_3_, three carbons); *m/z* calcd. for C_31_H_43_N_2_O_5_^+^ [M + H]^+^ 523.3166; found 523.3131.

#### 3.2.8. 6-[(3-Aminopropyl)oxy]-2-[(1-benzylpiperidin-4-yl)methyl]-5-methoxy-2,3-dihydroinden-1-one (**8a**)

A solution of the 2-[(1-benzylpiperidin-4-yl)methyl]-6-[(3-*tert*-butyl-*N*-propylcarbamate)oxy]-5-methoxy-2,3-dihydroinden-1-one (100 mg, 0.19 mmol) in CH_2_Cl_2_ (2 mL) was treated with TFA (1 mL) and allowed to stir at RT for 5 min. The reaction was then quenched by addition of saturated aqueous NaHCO_3_ and the resulting mixture was extracted with CH_2_Cl_2_ (3×). The combined organic layers were washed with brine, dried over anhydrous MgSO_4_, filtered, and concentrated under reduced pressure. The crude product obtained was purified by column chromatography (SiO_2_ gel, pure CH_2_Cl_2_ to CH_2_Cl_2_:MeOH/19:1; R_f_ 0.12 in CH_2_Cl_2_:MeOH/9:1) to yield compound **8a** (53 mg, 65%) as a white solid: ^1^H-NMR (400 MHz, CDCl_3_, Figure S13) δ 7.28–7.18 (m, 5H, aromatic), 7.13 (s, 1H, aromatic), 6.81 (s, 1H, aromatic), 4.09 (t, *J* = 6.4 Hz, 2H, OCH_2_CH_2_), 3.90 (s, 3H, OCH_3_), 3.47 (s, 2H, NCH_2_Ph), 3.19 (dd, *J*_1_ = 17.6 Hz, *J*_2_ = 8.0 Hz, 1H), 2.84–2.90 (m, 4H), 2.67 (m, 2H), 1.99–1.84 (m, 7H), 1.72–1.58 (m, 2H), 1.45 (m, 1H), 1.38–1.23 (m, 3H); 13C-NMR (100 MHz, CDCl_3_, Figure S14) δ 207.8 (C=O), 155.7 (C), 148.73 (C), 148.66 (C), 138.4 (C), 129.2 (CH, three carbons), 128.1 (CH, two carbons), 126.9 (C), 107.5 (CH), 105.6 (CH), 67.4 (CH_2_), 63.4 (CH_2_), 56.2 (CH_3_), 53.76 (CH_2_), 53.74 (CH_2_), 45.4 (CH), 39.4 (CH_2_), 38.7 (CH_2_), 34.4 (CH_2_), 33.3 (CH_2_), 33.0 (CH_2_), 32.5 (CH_2_), 31.8 (CH); *m/z* calcd. for C_26_H_35_N_2_O_3_^+^ [M + H]^+^ 423.2642; found 423.2656. The purity of the compound was further confirmed by RP-HPLC: *R*_t_ = 15.86 min (96%; Figure S15).

#### 3.2.9. *tert*-Butyl *N*-(2-chloroethyl)carbamate. 

A solution of NaHCO_3_ (6.7 g, 79.3 mmol) in H_2_O (15 mL) was slowly added to a mixture of 2-chloroethylamine hydrochloride (1.0 g, 8.6 mmol), Boc_2_O (3.4 g, 15.5 mmol) and 1,4-dioxane (10 mL) at 0 °C. The resulting mixture was allowed to warm to RT and was stirred overnight. The reaction mixture was then diluted with H_2_O, and extracted with CH_2_Cl_2_ (3×). The combined organic layers were washed with H_2_O (3×) and brine (3×), dried over anhydrous MgSO_4_, filtered, and concentrated under reduced pressure. The crude product obtained was purified by column chromatography (SiO_2_ gel, Hexanes:EtOAc/9:1; R_f_ 0.55 in hexanes:EtOAc/4:1) to yield the known compound *tert*-butyl *N*-(2-chloroethyl)carbamate [28] (1.25 g, 83%) as a colorless oil: ^1^H-NMR (400 MHz, CDCl_3_, which matches the lit. [28], Figure S16) δ 4.94 (br s, 1H, NH), 3.57 (t, *J* = 6.0 Hz, 2H, ClCH_2_CH_2_), 3.44 (q, *J* = 6.0 Hz, 2H, CH_2_CH_2_NHBoc), 1.47 (s, 9H, C(CH_3_)_3_).

#### 3.2.10. 2-[(1-Benzylpiperidin-4-yl)methyl]-6-[(3-*tert*-butyl-*N*-ethylcarbamate)oxy]-5-methoxy-2,3-dihydroinden-1-one (Boc-protected compound **8b**). 

A solution of compound **7** (216 mg, 1.20 mmol), Cs_2_CO_3_ (196 mg, 0.60 mmol), and TBAI (56 mg, 0.15 mmol) in anhydrous DMF (5 mL) was stirred at RT overnight. The reaction mixture was then diluted with H_2_O, and extracted with EtOAc (3×). The combined organic layers were washed with H_2_O (3×) and brine (3×), dried over anhydrous MgSO_4_, filtered, and concentrated under reduced pressure. The crude product obtained was purified by column chromatography (SiO_2_ gel, pure CH_2_Cl_2_ to CH_2_Cl_2_:MeOH/19:1; R_f_ 0.48 in CH_2_Cl_2_:MeOH/9:1) to yield 2-[(1-benzylpiperidin-4-yl)methyl]-6-[(3-*tert*-butyl-*N*-ethylcarbamate)oxy]-5-methoxy-2,3-dihydroinden-1-one (93 mg, 61%) as a pale yellow solid: ^1^H-NMR (400 MHz, CDCl_3_, Figure S17) δ 7.34–7.20 (m, 5H, aromatic), 7.14 (s, 1H, aromatic), 6.83 (s, 1H, aromatic), 5.05 (m, 1H, NH), 4.05 (br t, 2H, OCH_2_CH_2_), 3.92 (s, 3H, OCH_3_), 3.53 (m, 4H, NCH_2_Ph, CH_2_CH_2_NHBoc), 3.21 (dd, *J*_1_ = 17.2 Hz, *J*_2_ = 8.4 Hz, 1H), 2.90 (m, 2H), 2.67 (m, 2H), 1.99 (m, 2H), 1.88 (m, 1H), 1.69 (m, 2H), 1.43 (m, 10H), 1.37–1.23 (m, 3H); 13C-NMR (100 MHz, CDCl_3_, Figure S18) δ 207.6 (C=O), 155.8 (C and C=O), 149.2 (C), 148.3 (C), 138.3 (C), 129.3 (CH, two carbons), 128.1 (CH, three carbons), 126.9 (C), 107.7 (CH), 106.7 (CH), 79.5 (C), 68.8 (CH_2_), 63.4 (CH_2_), 56.1 (CH_3_), 53.7 (CH_2_, two carbons), 45.4 (CH), 39.9 (CH_2_), 38.7 (CH_2_), 34.4 (CH_2_), 33.3 (CH_2_), 32.9 (CH_2_), 31.7 (CH), 28.4 (CH_3_, three carbons); *m/z* calcd. for C_30_H_41_N_2_O_5_^+^ [M + H]^+^ 509.3010; found 509.3025.

#### 3.2.11. 6-[(3-Aminoethyl)oxy]-2-[(1-benzylpiperidin-4-yl)methyl]-5-methoxy-2,3-dihydroinden-1-one (**8b**)

A solution of 2-[(1-benzylpiperidin-4-yl)methyl]-6-[(3-*tert*-butyl-*N*-ethylcarbamate)oxy]-5-methoxy-2,3-dihydroinden-1-one (83 mg, 0.16 mmol) in CH_2_Cl_2_ (1 mL) was treated with TFA (1 mL) and allowed to stir at RT. After 1 h, the reaction was analyzed by TLC (CH_2_Cl_2_:MeOH/9:1, R_f_ 0.19 in CH_2_Cl_2_:MeOH/9:1). The reaction was then quenched by addition of saturated aqueous NaHCO_3_ and the resulting mixture was extracted with CH_2_Cl_2_ (3×). The combined organic layers were washed with brine, dried over anhydrous MgSO_4_, filtered, and concentrated under reduced pressure to yield compound **8b** (62 mg, 93%) as a white solid: ^1^H-NMR (400 MHz, CDCl_3_, Figure S19) δ 7.32–7.20 (m, 5H, aromatic), 7.15 (s, 1H, aromatic), 6.83 (s, 1H, aromatic), 4.03 (t, *J* = 5.2 Hz, 2H, OCH_2_CH_2_), 3.92 (s, 3H, OCH_3_), 3.57 (s, 2H, NCH_2_Ph), 3.21 (dd, *J*_1_ = 17.6 Hz, *J*_2_ = 8.0 Hz, 1H), 3.11 (t, *J* = 5.2 Hz, 2H, OCH_2_CH_2_), 2.95 (m, 2H), 2.67 (m, 2H), 2.03 (m, 2H), 1.89 (m, 1H), 1.71 (m, 2H), 1.52 (m, 2H), 1.44–1.23 (m, 4H); ^13^C-NMR (100 MHz, CDCl_3_, Figure S20) δ 207.7 (C=O), 155.8 (C), 148.9 (C), 148.6 (C), 138.1 (C), 129.3 (CH, four carbons), 129.2 (CH), 128.1 (CH, two carbons), 127.0 (C), 107.6 (CH), 106.0 (CH), 71.1 (CH_2_), 63.3 (CH_2_), 56.1 (CH_3_), 53.68 (CH_2_), 53.65 (CH_2_), 45.4 (CH), 41.2 (CH_2_), 38.6 (CH_2_), 34.3 (CH_2_), 33.3 (CH_2_), 32.8 (CH_2_), 31.7 (CH); *m/z* calcd. for C_25_H_33_N_2_O_3_^+^ [M + H]^+^ 409.2486; found 409.2496. The purity of the compound was further confirmed by RP-HPLC: *R*_t_ = 15.74 min (95%; Figure S21).

#### 3.2.12. 2-[(1-Benzylpiperidin-4-yl)methyl]-5-methoxy-6-propoxy-2,3-dihydroinden-1-one (**8c**). 

A solution of compound **7** (50 mg, 0.14 mmol) and K_2_CO_3_ (95 mg, 0.68 mmol) in anhydrous DMF (5 mL) was treated with 1-bromopropane (0.06 mL, 0.68 mmol), and the resulting mixture was stirred at RT overnight. The reaction mixture was then diluted with H_2_O, and extracted with EtOAc (3×). The combined organic layers were washed with H_2_O (3×) and brine (3×), dried over anhydrous MgSO_4_, filtered, and concentrated under reduced pressure. The crude product obtained was purified by column chromatography (SiO_2_ gel, pure CH_2_Cl_2_ to CH_2_Cl_2_:MeOH/19:1; R_f_ 0.38 in CH_2_Cl_2_:MeOH/19:1) to yield compound **8c** (53 mg, 95%) as an off-white solid: ^1^H-NMR (400 MHz, CDCl_3_, Figure S22) δ 7.32–7.20 (m, 5H, aromatic), 7.13 (s, 1H, aromatic), 6.82 (s, 1H, aromatic), 3.98 (t, *J* = 6.8 Hz, 2H, CH_3_CH_2_CH_2_OAr), 3.92 (s, 3H, OCH_3_), 3.51 (s, 2H, NCH_2_Ph), 3.20 (dd, *J*_1_ = 17.6 Hz, *J*_2_ = 8.4 Hz, 1H), 2.90 (m, 2H), 2.66 (dt, *J*_1_ = 14.0 Hz, *J*_2_ = 3.6 Hz, 2H), 1.98 (m, 2H), 1.90 (m, 1H), 1.85 (sextet, *J* = 7.2 Hz, 2H, CH_3_CH_2_CH_2_OAr), 1.73–1.64 (m, 2H), 1.49 (m, 1H), 1.40–1.24 (m, 3H), 1.02 (t, *J* = 7.2 Hz, 3H, CH_3_CH_2_CH_2_OAr); ^13^C-NMR (100 MHz, CDCl_3_, Figure S23) δ 207.8 (C=O), 155.8 (C), 148.9 (C), 148.5 (C), 129.3 (CH, two carbons), 129.2 (C), 128.2 (CH, three carbons), 127.1 (C), 107.5 (CH), 105.5 (CH), 70.5 (CH_2_), 63.2 (CH_2_), 56.2 (CH_3_), 53.7 (CH_2_), 53.6 (CH_2_), 45.4 (CH), 38.6 (CH_2_), 34.3 (CH_2_), 33.3 (CH_2_), 32.7 (CH_2_), 31.6 (CH), 22.2 (CH_2_), 10.3 (CH_3_); *m/z* calcd. for C_26_H_34_NO_3_^+^ [M + H]^+^ 408.2533; found 408.2524. The purity of the compound was further confirmed by RP-HPLC: *R*_t_ = 19.30 min (96%; Figure S24).

#### 3.2.13. 2-[(1-Benzylpiperidin-4-yl)methyl]-6-[(chloroethyl)oxy]-5-methoxy-2,3-dihydroinden-1-one (**8d**)

A solution of compound **7** (50 mg, 0.14 mmol) and K_2_CO_3_ (189 mg, 1.37 mmol) in anhydrous DMF (5 mL) was treated with 1,2-dichloroethane (0.11 mL, 1.37 mmol), and the resulting mixture was stirred at RT overnight. The reaction mixture was then diluted with H_2_O, and extracted with CH_2_Cl_2_ (3×). The combined organic layers were washed with H_2_O (3×) and brine (3×), dried over anhydrous MgSO_4_, filtered, and concentrated under reduced pressure. The crude product obtained was purified by column chromatography (SiO_2_ gel, pure CH_2_Cl_2_ to CH_2_Cl_2_:MeOH/19:1; R_f_ 0.38 in CH_2_Cl_2_:MeOH/19:1) to yield compound **8d** (50 mg, 85%) as a brown oil: ^1^H-NMR (400 MHz, CDCl_3_, Figure S25) δ 7.30–7.20 (m, 5H, aromatic), 7.14 (s, 1H, aromatic), 6.84 (s, 1H, aromatic), 4.25 (t, *J* = 6.0 Hz, 2H), 3.91 (s, 3H, OCH_3_), 3.82 (t, *J* = 6.0 Hz, 2H), 3.49 (s, 2H, NCH_2_Ph), 3.20 (dd, *J*_1_ = 17.6 Hz, *J_2_* = 8.4 Hz, 1H), 2.88 (m, 2H), 2.66 (dt, *J*_1_ = 14.4 Hz, *J_2_* = 2.8 Hz, 2H), 1.99–1.93 (m, 2H), 1.92–1.85 (m, 1H), 1.72–1.63 (m, 2H), 1.47 (m, 1H), 1.40–1.23 (m, 3H); ^13^C-NMR (100 MHz, CDCl_3_, Figure S26) δ 207.6 (C=O), 155.9 (C), 149.5 (C), 148.0 (C), 138.1 (C), 129.3 (CH, two carbons), 129.2 (CH), 128.1 (CH, two carbons), 127.0 (C), 107.9 (CH), 106.6 (CH), 69.0 (CH_2_), 63.3 (CH_2_), 56.2 (CH_3_), 53.68 (CH_2_), 53.65 (CH_2_), 45.4 (CH), 41.4 (CH_2_), 38.6 (CH_2_), 34.3 (CH_2_), 33.3 (CH_2_), 32.8 (CH_2_), 31.7 (CH); *m/z* calcd. for C_25_H_31_ClNO_3_^+^ [M + H]^+^ 428.1987; found 428.1984. The purity of the compound was further confirmed by RP-HPLC: *R*_t_ = 18.90 min (96%; Figure S27).

#### 3.2.14. 2-[(1-Benzylpiperidin-4-yl)methyl]-6-[(bromoethyl)oxy]-5-methoxy-2,3-dihydroinden-1-one (**8e**)

A solution of compound **7** (100 mg, 0.27 mmol) and K_2_CO_3_ (380 mg, 2.74 mmol) in anhydrous DMF (5 mL) was treated with 1,2-dibromoethane (0.24 mL, 2.74 mmol) and the resulting mixture was stirred at RT overnight. The reaction mixture was then diluted with H_2_O, and extracted with CH_2_Cl_2_ (3×). The combined organic layers were washed with H_2_O (3×) and brine (3×), dried over anhydrous MgSO_4_, filtered, and concentrated under reduced pressure. The crude product obtained was purified by column chromatography (SiO_2_ gel, pure CH_2_Cl_2_ to CH_2_Cl_2_:MeOH/19:1; R_f_ 0.49 in CH_2_Cl_2_:MeOH/19:1) to yield compound **8e** (70 mg, 54%) as an off-white solid: ^1^H-NMR (400 MHz, CDCl_3_, Figure S28) δ 7.30-7.20 (m, 5H, aromatic), 7.15 (s, 1H, aromatic), 6.85 (s, 1H, aromatic), 4.32 (t, *J* = 6.4 Hz, 2H), 3.93 (s, 3H, OCH_3_), 3.65 (t, *J* = 6.4 Hz, 2H), 3.49 (s, 2H, NCH_2_Ph), 3.21 (dd, *J*_1_ = 17.6 Hz, *J_2_* = 8.0 Hz, 1H), 2.88 (m, 2H), 2.67 (dt, *J*_1_ = 14.0 Hz, *J_2_* = 2.8 Hz, 2H), 1.98–1.86 (m, 3H), 1.72–1.62 (m, 2H), 1.47 (m, 1H), 1.40–1.24 (m, 3H); ^13^C-NMR (100 MHz, CDCl_3_, Figure S29) δ 207.6 (C=O), 155.9 (C), 149.5 (C), 147.8 (C), 138.4 (C), 129.2 (CH, 3 carbons), 128.1 (CH, 2 carbons), 126.9 (C), 108.0 (CH), 106.6 (CH), 68.8 (CH_2_), 63.4 (CH_2_), 56.3 (CH_3_), 53.75 (CH_2_), 53.72 (CH_2_), 45.4 (CH), 38.7 (CH_2_), 34.4 (CH_2_), 33.4 (CH_2_), 33.0 (CH_2_), 31.8 (CH), 28.4 (CH_2_); *m/z* calcd. for C_25_H_31_BrNO_3_^+^ [M + H]^+^ 472.1482; found 472.1477. The purity of the compound was further confirmed by RP-HPLC: *R*_t_ = 19.10 min (97%; Figure S30).

#### 3.2.15. 2-[(1-Benzylpiperidin-4-yl)methyl]-6-[(bromobutyl)oxy]-5-methoxy-2,3-dihydroinden-1-one (**8f**)

A solution of compound **7** (50 mg, 0.14 mmol) and K_2_CO_3_ (189 mg, 1.37 mmol) in anhydrous DMF (5 mL) was treated with 1,4-dibromobutane (0.16 mL, 1.37 mmol), and the resulting mixture was stirred at RT overnight. The reaction mixture was then diluted with H_2_O, and extracted with CH_2_Cl_2_ (3×). The combined organic layers were washed with H_2_O (3×) and brine (3×), dried over anhydrous MgSO_4_, filtered, and concentrated under reduced pressure. The crude product obtained was purified by column chromatography (SiO_2_ gel, pure CH_2_Cl_2_ to CH_2_Cl_2_:MeOH/19:1; R_f_ 0.38 in CH_2_Cl_2_:MeOH/19:1) to yield compound **8f** (63 mg, 93%) as an off-white solid: ^1^H-NMR (400 MHz, CDCl_3_, Figure S31) δ 7.32–7.22 (m, 5H, aromatic), 7.13 (s, 1H, aromatic), 6.83 (s, 1H, aromatic), 4.04 (t, *J* = 6.4 Hz, 2H), 3.92 (s, 3H, OCH_3_), 3.52 (s, 2H, NCH_2_Ph), 3.48 (t, *J* = 6.4 Hz, 2H), 3.20 (dd, *J*_1_ = 17.6 Hz, *J_2_* = 8.0 Hz, 1H), 2.90 (m, 2H), 2.67 (m, 2H), 2.10–1.93 (m, 6H), 1.92–1.85 (m, 1H), 1.73–1.64 (m, 2H), 1.48 (m, 1H), 1.40–1.23 (m, 3H); ^13^C-NMR (100 MHz, CDCl_3_, Figure S32) δ 207.8 (C=O), 155.8 (C), 148.8 (C), 148.6 (C), 129.4 (CH, two carbons), 129.2 (C), 128.2 (CH, three carbons), 127.2 (C), 107.5 (CH), 105.6 (CH), 68.0 (CH_2_), 63.2 (CH_2_), 56.2 (CH_3_), 53.6 (CH_2_, two carbons), 45.3 (CH), 38.6 (CH_2_), 34.2 (CH_2_), 33.3 (CH_2_, two carbons), 32.6 (CH_2_), 31.5 (CH), 29.4 (CH_2_), 27.6 (CH_2_); *m/z* calcd. for C_27_H_35_BrNO_3_^+^ [M + H]^+^ 500.1795; found 500.1794. The purity of the compound was further confirmed by RP-HPLC: *R*_t_ = 20.22 min (95%; Figure S33).

#### 3.2.16. 2-[(1-Benzylpiperidin-4-yl)methyl]-6-[(bromododecyl)oxy]-5-methoxy-2,3-dihydroinden-1-one (**8g**)

A solution of compound **7** (100 mg, 0.27 mmol) and K_2_CO_3_ (380 mg, 2.74 mmol) in anhydrous DMF (5 mL) was treated with 1,12-dibromododecane (900 mg, 2.74 mmol) and the resulting mixture was stirred at RT overnight. The reaction mixture was then diluted with H_2_O, and extracted with CH_2_Cl_2_ (3×). The combined organic layers were washed with H_2_O (3×) and brine (3×), dried over anhydrous MgSO_4_, filtered, and concentrated under reduced pressure. The crude product obtained was purified by column chromatography (SiO_2_ gel, pure CH_2_Cl_2_ to CH_2_Cl_2_:MeOH/19:1; R_f_ 0.49 in CH_2_Cl_2_:MeOH/19:1) to yield compound **8g** (53 mg, 32%) as an off-white solid: ^1^H-NMR (400 MHz, CDCl_3_, Figure S34) δ 7.32–7.22 (m, 5H, aromatic), 7.13 (s, 1H, aromatic), 6.82 (s, 1H, aromatic), 3.99 (t, *J* = 6.8 Hz, 2H), 3.92 (s, 3H, OCH_3_), 3.53 (s, 2H, NCH_2_Ph), 3.38 (t, *J* = 6.8 Hz, 2H), 3.20 (dd, *J*_1_ = 17.6 Hz, *J*_2_ = 8.0 Hz, 1H), 2.91 (m, 2H), 2.66 (dt, *J*_1_ = 14.0 Hz, *J*_2_ = 3.6 Hz, 2H), 1.99 (m, 2H), 1.92–1.78 (m, 5H), 1.78–1.62 (m, 3H), 1.44–1.38 (m, 6H), 1.36–1.22 (m, 13H); ^13^C-NMR (100 MHz, CDCl_3_, Figure S35) δ 207.8 (C=O), 155.8 (C), 148.9 (C), 148.5 (C), 129.3 (CH, two carbons), 129.2 (C), 128.2 (CH, three carbons), 127.1 (C), 107.4 (CH), 105.5 (CH), 69.1 (CH_2_), 63.2 (CH_2_), 56.2 (CH_3_), 53.6 (CH_2_, two carbons), 45.4 (CH), 38.6 (CH_2_), 34.3 (CH_2_), 34.1 (CH_2_), 33.3 (CH_2_), 32.8 (CH_2_), 32.6 (CH_2_), 31.6 (CH), 29.5 (CH_2_, two carbons), 29.4 (CH_2_), 29.3 (CH_2_), 28.9 (CH_2_), 28.7 (CH_2_), 28.1 (CH_2_), 26.9 (CH_2_), 25.9 (CH_2_); *m/z* calcd. for C_35_H_51_BrNO_3_^+^ [M + H]^+^ 612.3047; found 612.3045. The purity of the compound was further confirmed by RP-HPLC: *R*_t_ = 25.00 min (96%; Figure S36).

#### 3.2.17. 6-[(Benzyl)oxy-2-[(1-benzylpiperidin-4-yl)methyl]-5-methoxy-2,3-dihydroinden-1-one (**8h**)

A solution of compound **7** (50 mg, 0.14 mmol) and K_2_CO_3_ (38 mg, 0.27 mmol) in anhydrous DMF (5 mL) was treated with benzyl bromide (20 μL, 0.16 mmol), and the resulting mixture was stirred at RT overnight. The reaction mixture was then diluted with H_2_O, and extracted with EtOAc (3×). The combined organic layers were washed with H_2_O (3×) and brine (3×), dried over anhydrous MgSO_4_, and filtered. After standing at RT overnight, white solids precipitated out, which were filtered off. The filtrate was further concentrated under reduced pressure, and purified by column chromatography (SiO_2_ gel, pure CH_2_Cl_2_ to CH_2_Cl_2_:MeOH/19:1; R_f_ 0.37 in CH_2_Cl_2_:MeOH/19:1) to yield compound **8h** (51 mg, 82%) as an off-white solid: ^1^H-NMR (400 MHz, CDCl_3_, Figure S37) δ 7.42 (d, *J* = 7.6 Hz, 2H, aromatic), 7.35 (t, *J* = 7.6 Hz, 2H, aromatic), 7.31–7.28 (m, 5H, aromatic), 7.24 (s, 1H, aromatic), 7.19 (s, 1H, aromatic), 6.85 (s, 1H, aromatic), 5.13 (s, 2H, OCH_2_Ph), 3.93 (s, 3H, OCH_3_), 3.51 (s, 2H, NCH_2_Ph), 3.20 (dd, *J*_1_ = 17.6 Hz, *J*_2_ = 8.4 Hz, 1H), 2.89 (m, 2H), 2.63 (dt, *J*_1_ = 14.0 Hz, *J*_2_ = 4.0 Hz, 2H), 1.97 (m, 2H), 1.88 (m, 1H), 1.73–1.64 (m, 2H), 1.48 (m, 1H), 1.40–1.23 (m, 3H); ^13^C-NMR (100 MHz, CDCl_3_, Figure S38) δ 207.6 (C=O), 156.0 (C), 148.9 (C), 148.5 (C), 136.3 (C), 129.4 (CH, two carbons), 129.2 (C), 128.6 (CH, two carbons), 128.2 (CH, two carbons), 128.0 (CH, two carbons), 127.4 (CH, two carbons), 127.1 (C), 107.6 (CH), 106.4 (CH), 70.8 (CH_2_), 63.2 (CH_2_), 56.2 (CH_3_), 53.6 (CH_2_, two carbons), 45.3 (CH), 38.6 (CH_2_), 34.3 (CH_2_), 33.4 (CH_2_), 32.7 (CH_2_), 31.6 (CH); *m/z* calcd. for C_30_H_34_NO_3_^+^ [M + H]^+^ 456.2533; found 456.2528. The purity of the compound was further confirmed by RP-HPLC: *R*_t_ = 20.05 min (96%; Figure S39).

#### 3.2.18. 2-[(1-Benzylpiperidin-4-yl)methyl]-5-methoxy-6-[(4-methylbenzyl)oxy-2,3-dihydroinden-1-one (**8i**)

A solution of compound **7** (50 mg, 0.14 mmol) and K_2_CO_3_ (38 mg, 0.27 mmol) in anhydrous DMF (5 mL) was treated with 4-methylbenzyl bromide (30 mg, 0.16 mmol), and the resulting mixture was stirred at RT overnight. The reaction mixture was then diluted with H_2_O, and extracted with CH_2_Cl_2_ (3×). The combined organic layers were washed with H_2_O (3×) and brine (3×), dried over anhydrous MgSO_4_, and filtered. After standing at RT overnight, the white solids precipitated out, which were filtered off. The filtrate was further concentrated under reduced pressure, and purified by column chromatography (SiO_2_ gel, pure CH_2_Cl_2_ to CH_2_Cl_2_:MeOH/19:1; R_f_ 0.35 in CH_2_Cl_2_:MeOH/19:1) to yield compound **8i** (37 mg, 58%) as an off-white solid: ^1^H-NMR (400 MHz, CDCl_3_, Figure S40) δ 7.31 (d, *J* = 8.0 Hz, 2H, aromatic), 7.31–7.29 (m, 4H, aromatic), 7.24 (s, 1H, aromatic), 7.18 (s, 1H, aromatic), 7.15 (d, *J* = 8.0 Hz, 2H, aromatic), 6.83 (s, 1H, aromatic), 5.09 (s, 2H, OCH_2_Ph), 3.92 (s, 3H, OCH_3_), 3.51 (s, 2H, NCH_2_Ph), 3.20 (dd, *J*_1_ = 17.6 Hz, *J*_2_ = 8.4 Hz, 1H), 2.89 (m, 2H), 2.63 (dt, *J*_1_ = 13.6 Hz, *J*_2_ = 3.6 Hz, 2H), 2.32 (s, 3H, CH_3_Ph), 1.97 (m, 2H), 1.91–1.84 (m, 1H), 1.72–1.64 (m, 2H), 1.47 (m, 1H), 1.40–1.23 (m, 3H); ^13^C-NMR (100 MHz, CDCl_3_, Figure S41) δ 207.6 (C=O), 156.0 (C), 148.9 (C), 148.5 (C), 137.8 (C), 133.3 (C), 129.30 (CH, two carbons), 129.25 (CH, two carbons), 129.17 (C), 128.2 (CH, three carbons), 127.5 (CH, 2 carbons), 127.0 (C), 107.6 (CH), 106.4 (CH), 70.7 (CH_2_), 63.3 (CH_2_), 56.2 (CH_3_), 53.7 (CH_2_, 2 carbons), 45.4 (CH), 38.7 (CH_2_), 34.3 (CH_2_), 33.4 (CH_2_), 32.8 (CH_2_), 31.7 (CH), 21.2 (CH_3_); *m/z* calcd. for C_31_H_36_NO_3_^+^ [M + H]^+^ 470.2690; found 470.2681. The purity of the compound was further confirmed by RP-HPLC: *R*_t_ = 20.65 min (95%; Figure S42).

#### 3.2.19. 2-[(1-Benzylpiperidin-4-yl)methyl]-5-methoxy-6-[(4-methoxybenzyl)oxy-2,3-dihydroinden-1-one (**8j**)

A solution of compound **7** (50 mg, 0.14 mmol) and K_2_CO_3_ (189 mg, 1.37 mmol) in anhydrous DMF (5 mL) was treated with 4-methoxybenzyl chloride (0.19 mL, 1.37 mmol), and the resulting mixture was stirred at RT overnight. The reaction mixture was then diluted with H_2_O, and extracted with CH_2_Cl_2_ (3×). The combined organic layers were washed with H_2_O (3×) and brine (3×), dried over anhydrous MgSO_4_, filtered, and concentrated under reduced pressure. The crude product obtained was purified by column chromatography (SiO_2_ gel, pure CH_2_Cl_2_ to CH_2_Cl_2_:MeOH/19:1; R_f_ 0.30 in CH_2_Cl_2_:MeOH/19:1) to yield compound **8j** (24 mg, 36%) as an off-white solid: ^1^H-NMR (400 MHz, CDCl_3_, Figure S43) δ 7.34 (d, *J* = 8.8 Hz, 2H, aromatic), 7.33–7.26 (m, 5H, aromatic), 7.19 (s, 1H, aromatic), 6.87 (d, *J* = 8.8 Hz, 2H, aromatic), 6.83 (s, 1H, aromatic), 5.05 (s, 2H, OCH_2_Ph), 3.91 (s, 3H, OCH_3_), 3.78 (s, 3H, OCH_3_), 3.56 (s, 2H, NCH_2_Ph), 3.20 (dd, *J*_1_ = 17.6 Hz, *J*_2_ = 8.4 Hz, 1H), 2.95 (m, 2H), 2.65 (dt, *J*_1_ = 13.6 Hz, *J_2_* = 3.6 Hz, 2H), 2.08–1.98 (m, 2H), 1.92–1.82 (m, 1H), 1.76–1.64 (m, 2H), 1.53 (m, 1H), 1.40–1.23 (m, 3H); ^13^C-NMR (100 MHz, CDCl_3_, Figure S44) δ 207.6 (C=O), 159.5 (C), 156.0 (C), 148.9 (C), 148.5 (C), 137.2 (C), 129.5 (CH, two carbons), 129.2 (CH, two carbons), 129.1 (C), 128.4 (CH), 128.2 (CH, two carbons), 127.3 (C), 114.0 (CH, 2 carbons), 107.6 (CH), 106.5 (CH), 70.6 (CH_2_), 63.1 (CH_2_), 56.2 (CH_3_), 55.3 (CH_3_), 53.6 (CH_2_), 53.5 (CH_2_), 45.3 (CH), 38.6 (CH_2_), 34.1 (CH_2_), 33.4 (CH_2_), 32.4 (CH_2_), 31.4 (CH); *m/z* calcd. for C_31_H_36_NO_4_^+^ [M + H]^+^ 486.2639; found 486.2635. The purity of the compound was further confirmed by RP-HPLC: *R*_t_ = 19.93 min (95%; Figure S45).

#### 3.2.20. 2-[(1-Benzylpiperidin-4-yl)methyl]-5-methoxy-6-[(4-nitrobenzyl)oxy-2,3-dihydroinden-1-one (**8k**). 

A solution of compound **7** (50 mg, 0.14 mmol) and K_2_CO_3_ (38 mg, 0.27 mmol) in anhydrous DMF (5 mL) was treated with 4-nitrobenzyl bromide (35 mg, 0.16 mmol), and the resulting mixture was stirred at RT overnight. The reaction mixture was then diluted with H_2_O, and extracted with EtOAc (3×). The combined organic layers were washed with H_2_O (3×) and brine (3×), dried over anhydrous MgSO_4_, and filtered. After standing at RT overnight, white solids precipitated out, which were filtered off. The filtrate was further concentrated under reduced pressure and purified by column chromatography (SiO_2_ gel, pure CH_2_Cl_2_ to CH_2_Cl_2_:MeOH/19:1; R_f_ 0.37 in CH_2_Cl_2_:MeOH/19:1) to yield compound **8k** (44 mg, 65%) as a brown foam: ^1^H-NMR (400 MHz, CDCl_3_, Figure S46) δ 8.22 (d, *J* = 8.4 Hz, 2H, aromatic), 7.60 (d, *J* = 8.4 Hz, 2H, aromatic), 7.31–7.28 (m, 4H, aromatic), 7.24 (s, 1H, aromatic), 7.14 (s, 1H, aromatic), 6.88 (s, 1H, aromatic), 5.23 (s, 2H, OCH_2_Ph), 3.97 (s, 3H, OCH_3_), 3.50 (s, 2H, NCH_2_Ph), 3.22 (dd, *J*_1_ = 17.6 Hz, *J*_2_ = 8.0 Hz, 1H), 2.89 (m, 2H), 2.68 (m, 2H), 1.96 (m, 2H), 1.87 (m, 1H), 1.72–1.63 (m, 2H), 1.47 (m, 1H), 1.36–1.23 (m, 3H); ^13^C-NMR (100 MHz, CDCl_3_, Figure S47) δ 207.5 (C=O), 155.9 (C), 149.5 (C), 147.8 (C), 147.6 (C), 143.8 (C), 129.3 (CH), 129.2 (CH), 128.2 (CH, two carbons), 127.5 (CH, three carbons), 127.0 (C), 123.9 (CH, two carbons + C), 107.9 (CH), 106.6 (CH), 69.5 (CH_2_), 63.3 (CH_2_), 56.3 (CH_3_), 53.7 (CH_2_, two carbons), 45.4 (CH), 38.6 (CH_2_), 34.3 (CH_2_), 33.4 (CH_2_), 32.8 (CH_2_), 31.6 (CH); *m/z* calcd. for C_30_H_33_N_2_O_5_^+^ [M + H]^+^ 501.2384; found 501.2385. The purity of the compound was further confirmed by RP-HPLC: *R*_t_ = 19.98 min (96%; Figure S48).

#### 3.2.21. 2-[(1-Benzylpiperidin-4-yl)methyl]-6-[(4-bromobenzyl)oxy-5-methoxy-2,3-dihydroinden-1-one (**8l**)

A solution of compound **7** (50 mg, 0.14 mmol) and K_2_CO_3_ (38 mg, 0.27 mmol) in anhydrous DMF (5 mL) was treated with 4-bromobenzyl bromide (41 mg, 0.16 mmol) and the resulting mixture was stirred at RT overnight. The reaction mixture was then diluted with H_2_O, and extracted with EtOAc (3×). The combined organic layers were washed with H_2_O (3×) and brine (3×), dried over anhydrous MgSO_4_, and filtered. After standing at RT overnight, white solids precipitated out, which were filtered off. The filtrate was further concentrated under reduced pressure and purified by column chromatography (SiO_2_ gel, pure CH_2_Cl_2_ to CH_2_Cl_2_:MeOH/19:1; R_f_ 0.37 in CH_2_Cl_2_:MeOH/19:1) to yield compound **8l** (61 mg, 84%) as an off-white solid: ^1^H-NMR (400 MHz, CDCl_3_, Figure S49) δ 7.47 (d, *J* = 8.4 Hz, 2H, aromatic), 7.30 (d, *J* = 8.4 Hz, 2H, aromatic), 7.31–7.28 (m, 4H, aromatic), 7.24 (s, 1H, aromatic), 7.14 (s, 1H, aromatic), 6.85 (s, 1H, aromatic), 5.07 (s, 2H, OCH_2_Ph), 3.94 (s, 3H, OCH_3_), 3.51 (s, 2H, NCH_2_Ph), 3.21 (dd, *J*_1_ = 17.6 Hz, *J*_2_ = 8.4 Hz, 1H), 2.89 (m, 2H), 2.67 (dt, *J*_1_ = 14.4 Hz, *J*_2_ = 4.0 Hz, 2H), 1.96 (m, 2H), 1.87 (m, 1H), 1.72–1.63 (m, 2H), 1.47 (m, 1H), 1.40–1.23 (m, 3H); ^13^C-NMR (100 MHz, CDCl_3_, Figure S50) δ 207.6 (C=O), 155.9 (C), 149.1 (C), 148.2 (C), 135.4 (C), 131.7 (CH, two carbons + C), 129.4 (CH), 129.2 (CH), 129.0 (CH, three carbons), 128.2 (CH, two carbons), 127.1 (C), 122.0 (C), 107.7 (CH), 106.5 (CH), 70.1 (CH_2_), 63.2 (CH_2_), 56.2 (CH_3_), 53.6 (CH_2_, two carbons), 45.3 (CH), 38.6 (CH_2_), 34.2 (CH_2_), 33.4 (CH_2_), 32.7 (CH_2_), 31.6 (CH); *m/z* calcd. for C_30_H_33_BrNO_3_^+^ [M + H]^+^ 534.1638; found 534.1650. The purity of the compound was further confirmed by RP-HPLC: *R*_t_ = 21.05 min (96%; Figure S51).

#### 3.2.22. 2-[(1-Benzylpiperidin-4-yl)methyl]-6-[(4-fluorobenzyl)oxy-5-methoxy-2,3-dihydroinden-1-one (**8m**)

A solution of compound **7** (50 mg, 0.14 mmol) and K_2_CO_3_ (38 mg, 0.27 mmol) in anhydrous DMF (5 mL) was treated with 4-fluorobenzyl bromide (20 μL, 0.16 mmol), and the resulting mixture was stirred at RT overnight. The reaction mixture was then diluted with H_2_O, and extracted with EtOAc (3×). The combined organic layers were washed with H_2_O (3×) and brine (3×), dried over anhydrous MgSO_4_, and filtered. The filtrate was concentrated under reduced pressure and purified by column chromatography (SiO_2_ gel, pure CH_2_Cl_2_ to CH_2_Cl_2_:MeOH/19:1; R_f_ 0.31 in CH_2_Cl_2_:MeOH/19:1) to yield compound **8m** (56 mg, 86%) as an off-white solid: ^1^H-NMR (400 MHz, CDCl_3_, Figure S52) δ 7.40 (dd, *J*_1_ = 8.8 Hz, *J*_2_ = 5.6 Hz, 2H, aromatic), 7.30 (m, 4H, aromatic), 7.24 (s, 1H, aromatic), 7.18 (s, 1H, aromatic), 7.04 (t, *J* = 8.8 Hz, 2H, aromatic), 6.85 (s, 1H, aromatic), 5.08 (s, 2H, OCH_2_Ph), 3.93 (s, 3H, OCH_3_), 3.51 (s, 2H, NCH_2_Ph), 3.21 (dd, *J*_1_ = 17.6 Hz, *J*_2_ = 8.4 Hz, 1H), 2.90 (m, 2H), 2.67 (dt, *J*_1_ = 14.0 Hz, *J*_2_ = 3.6 Hz, 2H), 1.98 (m, 2H), 1.88 (m, 1H), 1.69 (m, 2H), 1.49 (m, 1H), 1.36–1.23 (m, 3H); ^13^C-NMR (100 MHz, CDCl_3_, Figure S53) δ 207.6 (C=O), 163.8–161.3 (d, *^1^J*_C-F_ = 245.2 Hz, C, one carbon), 155.9 (C), 149.1 (C), 148.3 (C), 138.1 (C), 132.14–132.11 (d, *^4^J*_C-F_ = 3.8 Hz, C, one carbon), 129.4–129.27 (d, *^3^J*_C-F_ = 8.4 Hz, CH, two carbons), 129.28 (CH, two carbons), 129.2 (CH), 128.2 (CH, two carbons), 127.0 (C), 115.6–115.4 (d, *^2^J*_C-F_ = 21.2 Hz, CH, two carbons), 107.7 (CH), 106.4 (CH), 70.1 (CH_2_), 63.3 (CH_2_), 56.2 (CH_3_), 53.70 (CH_2_), 53.68 (CH_2_), 45.4 (CH), 38.7 (CH_2_), 34.3 (CH_2_), 33.3 (CH_2_), 32.8 (CH_2_), 31.7 (CH); *m/z* calcd. C_30_H_33_FNO_3_^+^ [M + H]^+^ 474.2439; found 474.2442. The purity of the compound was further confirmed by RP-HPLC: *R*_t_ = 20.14 min (96%; Figure S54).

#### 3.2.23. 2-[(1-Benzylpiperidin-4-yl)methyl]-6-[(3-fluorobenzyl)oxy-5-methoxy-2,3-dihydroinden-1-one (**8n**)

A solution of compound **7** (50 mg, 0.14 mmol) and K_2_CO_3_ (38 mg, 0.27 mmol) in anhydrous DMF (5 mL) was treated with 3-fluorobenzyl bromide (20 μL, 0.16 mmol), and the resulting mixture was stirred at RT overnight. The reaction mixture was then diluted with H_2_O, and extracted with EtOAc (3×). The combined organic layers were washed with H_2_O (3×) and brine (3×), dried over anhydrous MgSO_4_, and filtered. After standing at RT overnight, the white solids precipitated out, which were filtered off. The filtrate was further concentrated under reduced pressure, and purified by column chromatography (SiO_2_ gel, pure CH_2_Cl_2_ to CH_2_Cl_2_:MeOH/19:1; R_f_ 0.31 in CH_2_Cl_2_:MeOH/19:1) to yield compound **8n** (57 mg, 88%) as an off-white solid: ^1^H-NMR (400 MHz, CDCl_3_, Figure S55) δ 7.34–7.26 (m, 5H, aromatic), 7.24 (s, 1H, aromatic), 7.18–7.12 (m, 3H, aromatic), 6.98 (td, *J*_1_ = 8.4 Hz, *J*_2_ = 2.4 Hz, 1H, aromatic), 6.86 (s, 1H, aromatic), 5.12 (s, 2H, OCH_2_Ph), 3.95 (s, 3H, OCH_3_), 3.54 (s, 2H, NCH_2_Ph), 3.21 (dd, *J*_1_ = 17.6 Hz, *J*_2_ = 8.0 Hz, 1H), 2.92 (m, 2H), 2.67 (dt, *J*_1_ = 14.4 Hz, *J*_2_ = 4.4 Hz, 2H), 2.0 (m, 2H), 1.87 (m, 1H), 1.73–1.66 (m, 2H), 1.50 (m, 1H), 1.40-1.26 (m, 3H); ^13^C-NMR (100 MHz, CDCl_3_, Figure S56) δ 207.6 (C=O), 164.2–161.8 (d, *^1^J*_C-F_ = 245.2 Hz, C, 1 carbon), 155.9 (C), 149.2 (C), 148.2 (C), 139.0–138.9 (d, *^3^J*_C-F_ = 7.6 Hz, C, one carbon), 137.4 (C), 130.2–130.1 (d, *^3^J*_C-F_ = 7.6 Hz, CH, one carbon), 129.4 (CH, two carbons), 129.1 (CH), 128.2 (CH, two carbons), 127.2 (C), 122.7–122.6 (d, *^4^J*_C-F_ = 3.1 Hz, CH, one carbon), 115.0–114.8 (d, *^2^J*_C-F_ = 20.5 Hz, CH, one carbon), 114.2–114.0 (d, *^2^J*_C-F_ = 22.0 Hz, CH, one carbon), 107.7 (CH), 106.4 (CH), 69.9 (CH_2_), 63.1 (CH_2_), 56.2 (CH_3_), 53.6 (CH_2_), 53.5 (CH_2_), 45.3 (CH), 38.6 (CH_2_), 34.2 (CH_2_), 33.4 (CH_2_), 32.5 (CH_2_), 31.5 (CH); *m/z* calcd. for C_30_H_33_FNO_3_^+^ [M + H]^+^ 474.2439; found 474.2426. The purity of the compound was further confirmed by RP-HPLC: *R*_t_ = 20.20 min (96%; Figure S57).

#### 3.2.24. 2-[(1-Benzylpiperidin-4-yl)methyl]-6-[(2-fluorobenzyl)oxy-5-methoxy-2,3-dihydroinden-1-one (**8o**)

A solution of compound **7** (50 mg, 0.14 mmol) and K_2_CO_3_ (38 mg, 0.27 mmol) in anhydrous DMF (5 mL) was treated with 2-fluorobenzyl bromide (20 μL, 0.16 mmol), and the resulting mixture was stirred at RT overnight. The reaction mixture was then diluted with H_2_O, and extracted with EtOAc (3×). The combined organic layers were washed with H_2_O (3×) and brine (3×), dried over anhydrous MgSO_4_, and filtered. After standing at RT overnight, white solids precipitated out, which were filtered off. The filtrate was further concentrated under reduced pressure and purified by column chromatography (SiO_2_ gel, pure CH_2_Cl_2_ to CH_2_Cl_2_:MeOH/19:1; R_f_ 0.31 in CH_2_Cl_2_:MeOH/19:1) to yield compound **8o** (56 mg, 86%) as an off-white solid: ^1^H-NMR (400 MHz, CDCl_3_, Figure S58) δ 7.49 (t, *J* = 7.6 Hz, 1H, aromatic), 7.31–7.25 (m, 5H, aromatic), 7.24 (s, 1H, aromatic), 7.23 (d, *J* = 7.6 Hz, 1H, aromatic), 7.12 (t, *J* = 7.6 Hz, 1H, aromatic), 7.06 (t, *J* = 8.4 Hz, 1H, aromatic), 6.85 (s, 1H, aromatic), 5.18 (s, 2H, OCH_2_Ph), 3.93 (s, 3H, OCH_3_), 3.52 (s, 2H, NCH_2_Ph), 3.21 (dd, *J*_1_ = 17.6 Hz, *J*_2_ = 8.0 Hz, 1H), 2.90 (m, 2H), 2.67 (m, 2H), 1.98 (m, 2H), 1.88 (m, 1H), 1.73–1.65 (m, 2H), 1.49 (m, 1H), 1.36–1.23 (m, 3H); ^13^C-NMR (100 MHz, CDCl_3_, Figure S59) δ 207.6 (C=O), 161.7–159.2 (d, ^1^*J*_C-F_ = 245.9 Hz, C, one carbon), 156.0 (C), 149.2 (C), 148.3 (C), 129.8 (CH), 129.7 (CH), 129.57-129.53 (d, *^3^J*_C-F_ = 3.8 Hz, CH, one carbon), 129.3 (C, two carbons), 129.2 (CH), 128.2 (CH, two carbons), 127.1 (C), 124.23–124.20 (d, *^3^J*_C-F_ = 3.8 Hz, CH, one carbon), 123.66–123.51 (d, *^2^J*_C-F_ = 14.4 Hz, CH, one carbon), 115.5–115.3 (d, *^2^J*_C-F_ = 20.5 Hz, CH, one carbon), 107.7 (CH), 106.6 (CH), 64.80–64.75 (d, *^3^J*_C-F_ = 4.5 Hz, CH_2_, one carbon), 63.2 (CH_2_), 56.2 (CH_3_), 53.6 (CH_2_, two carbons), 45.4 (CH), 38.6 (CH_2_), 34.3 (CH_2_), 33.4 (CH_2_), 32.7 (CH_2_), 31.6 (CH); *m/z* calcd. for C_30_H_33_FNO_3_^+^ [M + H]^+^ 474.2439; found 474.2429. The purity of the compound was further confirmed by RP-HPLC: *R*_t_ = 20.06 min (96%; Figure S60).

#### 3.2.25. 2-[(1-Benzylpiperidin-4-yl)methyl]-6-[(2-trifluoromethylbenzyl)oxy-5-methoxy-2,3-dihydroinden-1-one (**8p**)

A solution of compound **7** (50 mg, 0.14 mmol) and K_2_CO_3_ (38 mg, 0.27 mmol) in anhydrous DMF (5 mL) was treated with 2-trifluoromethylbenzyl bromide (25 μL, 0.16 mmol) and the resulting mixture was stirred at RT overnight. The reaction mixture was then diluted with H_2_O, and extracted with EtOAc (3×). The combined organic layers were washed with H_2_O (3×) and brine (3×), dried over anhydrous MgSO_4_, and filtered. The filtrate was concentrated under reduced pressure, and purified by column chromatography (SiO_2_ gel, pure CH_2_Cl_2_ to CH_2_Cl_2_:MeOH/19:1; R_f_ 0.41 in CH_2_Cl_2_:MeOH/19:1) to yield compound **8p** (65 mg, 90%) as an off-white solid: ^1^H-NMR (400 MHz, CDCl_3_, Figure S61) δ 7.75 (d, *J* = 7.6 Hz, 1H, aromatic), 7.67 (d, *J* = 8.0 Hz, 1H, aromatic), 7.54 (t, *J* = 8.0 Hz, 1H, aromatic), 7.39 (t, *J* = 7.6 Hz, 1H, aromatic), 7.31–7.27 (m, 4H, aromatic), 7.24 (m, 1H, aromatic), 7.18 (s, 1H, aromatic), 6.88 (s, 1H, aromatic), 5.31 (s, 2H, OCH_2_Ph), 3.96 (s, 3H, OCH_3_), 3.51 (s, 2H, NCH_2_Ph), 3.22 (dd, *J*_1_ = 17.6 Hz, *J*_2_ = 8.0 Hz, 1H), 2.90 (m, 2H), 2.68 (dt, *J*_1_ = 14.4 Hz, *J*_2_ = 3.2 Hz, 2H), 1.97 (m, 2H), 1.88 (m, 1H), 1.69 (m, 2H), 1.50 (m, 1H), 1.40–1.24 (m, 3H); 13C-NMR (100 MHz, CDCl_3_, Figure S62) δ 207.6, 156.0, 149.4, 148.2, 138.1, 135.07, 135.06, 132.1, 129.3 (two carbons), 129.2, 128.4, 128.2 (two carbons), 127.8, 127.5, 127.2, 127.0, 126.01, 125.95, 125.90, 125.8, 125.6, 122.9, 107.8, 106.6, 67.04, 67.01, 63.3, 56.2, 53.71. 53.68, 45.4, 38.7, 34.3, 33.4, 32.8, 31.7; *m/z* calcd. for C_31_H_33_F_3_NO_3_^+^ [M + H]^+^ 524.2407; found 524.2401. The purity of the compound was further confirmed by RP-HPLC: *R*_t_ = 20.90 min (96%; Figure S63).

#### 3.2.26. 2-[(1-Benzylpiperidin-4-yl)methyl]-6-[(2,4-difluorobenzyl)oxy-5-methoxy-2,3-dihydroinden-1-one (**8q**)

A solution of compound **7** (50 mg, 0.14 mmol) and K_2_CO_3_ (38 mg, 0.27 mmol) in anhydrous DMF (5 mL) was treated with 2,4-difluorobenzyl bromide (21 μL, 0.16 mmol) and the resulting mixture was stirred at RT overnight. The reaction mixture was then diluted with H_2_O, and extracted with EtOAc (3×). The combined organic layers were washed with H_2_O (3×) and brine (3×), dried over anhydrous MgSO_4_, and filtered. The filtrate was concentrated under reduced pressure, and purified by column chromatography (SiO_2_ gel, pure CH_2_Cl_2_ to CH_2_Cl_2_:MeOH/19:1; R_f_ 0.17 in CH_2_Cl_2_:MeOH/19:1) to yield compound **8q** (57 mg, 85%) as an off-white solid: ^1^H-NMR (400 MHz, CDCl_3_, Figure S64) δ 7.46 (dd, *J*_1_ = 14.8 Hz, *J*_2_ = 8.4 Hz, 1H, aromatic), 7.31–7.27 (m, 4H, aromatic), 7.24 (s, 1H, aromatic), 7.22 (s, 1H, aromatic), 6.88–6.79 (m, 3H, aromatic), 5.12 (s, 2H, OCH_2_Ph), 3.93 (s, 3H, OCH_3_), 3.52 (s, 2H, NCH_2_Ph), 3.22 (dd, *J*_1_ = 17.6 Hz, *J*_2_ = 8.0 Hz, 1H), 2.90 (m, 2H), 2.67 (dt, *J*_1_ = 14.4 Hz, *J*_2_ = 3.2 Hz, 2H), 1.98 (m, 2H), 1.89 (m, 1H), 1.69 (m, 2H), 1.49 (m, 1H), 1.40–1.26 (m, 3H); 13C-NMR (100 MHz, CDCl_3_, Figure S65) δ 207.6, 164.2, 164.0, 161.9, 161.8, 161.7, 161.6, 159.4, 159.3, 156.0, 149.3, 148.2, 137.9, 130.81, 130.75, 130.72, 130.66, 129.3 (two carbons), 129.2, 128.2 (two carbons), 127.0, 119.7, 119.6, 119.54, 119.50, 111.55, 111.51, 111.34, 111.30, 107.7, 106.6, 104.2, 103.9, 103.7, 64.34, 64.30, 63.3, 56.2, 53.7, 53.6, 45.4, 38.6, 34.3, 33.4, 32.8, 31.6; *m/z* calcd. for C_30_H_32_F_2_NO_3_^+^ [M + H]^+^ 492.2345; found 492.2353. The purity of the compound was further confirmed by RP-HPLC: *R*_t_ = 20.25 min (95%; Figure S66).

#### 3.2.27. 2-[(1-Benzylpiperidin-4-yl)methyl]-6-[(2,5-difluorobenzyl)oxy-5-methoxy-2,3-dihydroinden-1-one (**8r**)

A solution of compound **7** (50 mg, 0.14 mmol) and K_2_CO_3_ (38 mg, 0.27 mmol) in anhydrous DMF (5 mL) was treated with 2,5-difluorobenzyl bromide (21 μL, 0.16 mmol), and the resulting mixture was stirred at RT overnight. The reaction mixture was then diluted with H_2_O, and extracted with EtOAc (3×). The combined organic layers were washed with H_2_O (3×) and brine (3×), dried over anhydrous MgSO_4_, and filtered. The filtrate was concentrated under reduced pressure and purified by column chromatography (SiO_2_ gel, pure CH_2_Cl_2_ to CH_2_Cl_2_:MeOH/19:1; R_f_ 0.17 in CH_2_Cl_2_:MeOH/19:1) to yield compound **8r** (67 mg, 85%) as an off-white solid: ^1^H-NMR (400 MHz, CDCl_3_, Figure S67) δ 7.32–7.28 (m, 4H, aromatic), 7.24 (m, 2H, aromatic), 7.19 (s, 1H, aromatic), 7.02 (td, *J*_1_ = 8.8 Hz, *J*_2_ = 4.0 Hz, 1H, aromatic), 6.97–6.91 (m, 1H, aromatic), 6.87 (s, 1H, aromatic), 5.16 (s, 2H, OCH_2_Ph), 3.95 (s, 3H, OCH_3_), 3.54 (s, 2H, NCH_2_Ph), 3.22 (dd, *J*_1_ = 17.6 Hz, *J*_2_ = 8.0 Hz, 1H), 2.92 (m, 2H), 2.68 (dt, *J*_1_ = 14.0 Hz, *J*_2_ = 3.6 Hz, 2H), 2.05 (m, 2H), 1.88 (m, 1H), 1.70 (m, 2H), 1.51 (m, 1H), 1.40–1.24 (m, 3H); 13C-NMR (100 MHz, CDCl_3_, Figure S68) δ 207.5, 160.02, 160.00, 157.62, 157.60, 157.21, 157.19, 155.9, 154.79, 154.77, 149.4, 148.0, 137.7, 129.4 (two carbons), 129.2, 128.2 (two carbons), 127.1, 125.6, 125.5, 125.4, 125.3, 116.6, 116.5, 116.3, 116.2, 116.0, 115.9, 115.8, 115.7, 115.6, 115.43, 115.38, 107.8, 106.5, 64.2, 64.1, 63.2, 56.2, 53.60, 53.57, 45.3, 38.6, 34.2, 33.4, 32.6, 31.5; *m/z* calcd. for C_30_H_32_F_2_NO_3_^+^ [M + H]^+^ 492.2345; found 492.2350. Purity of the compound was further confirmed by RP-HPLC: *R*_t_ = 20.28 min (96%; Figure S69).

#### 3.2.28. 2-[(1-Benzylpiperidin-4-yl)methyl]-6-[(2,6-difluorobenzyl)oxy-5-methoxy-2,3-dihydroinden-1-one (**8s**)

A solution of compound **7** (50 mg, 0.14 mmol) and K_2_CO_3_ (38 mg, 0.27 mmol) in anhydrous DMF (5 mL) was treated with 2,6-difluorobenzyl bromide (34 mg, 0.16 mmol), and the resulting mixture was stirred at RT overnight. The reaction mixture was then diluted with H_2_O, and extracted with EtOAc (3×). The combined organic layers were washed with H_2_O (3×) and brine (3×), dried over anhydrous MgSO_4_, and filtered. The filtrate was concentrated under reduced pressure and purified by column chromatography (SiO_2_ gel, pure CH_2_Cl_2_ to CH_2_Cl_2_:MeOH/19:1; R_f_ 0.34 in CH_2_Cl_2_:MeOH/19:1) to yield compound **8s** (62 mg, 93%) as an off-white solid: ^1^H-NMR (400 MHz, CDCl_3_, Figure S70) δ 7.32–7.27 (m, 6H, aromatic), 7.24 (m, 1H, aromatic), 6.90 (t, *J* = 8.0 Hz, 2H, aromatic), 6.84 (s, 1H, aromatic), 5.14 (s, 2H, OCH_2_Ph), 3.88 (s, 3H, OCH_3_), 3.53 (s, 2H, NCH_2_Ph), 3.22 (dd, *J*_1_ = 17.6 Hz, *J*_2_ = 8.0 Hz, 1H), 2.91 (m, 2H), 2.68 (dt, *J*_1_ = 14.4 Hz, *J*_2_ = 4.4 Hz, 2H), 2.00 (m, 2H), 1.89 (m, 1H), 1.70 (m, 2H), 1.50 (m, 1H), 1.40–1.24 (m, 3H); 13C-NMR (100 MHz, CDCl_3_, Figure S71) δ 207.6, 163.3, 163.2, 160.8, 160.7, 156.2, 149.5, 148.4, 137.7, 131.0, 130.9, 130.8, 129.4 (two carbons), 129.2, 128.2 (two carbons), 127.1, 112.4, 112.2, 112.0, 111.6, 111.5, 111.4, 111.3, 107.8, 107.3, 63.2, 59.19, 59.15, 59.11, 56.2, 53.61, 53.58, 45.4, 38.6, 34.2, 33.4, 32.7, 31.6; *m/z* calcd. for C_30_H_32_F_2_NO_3_^+^ [M + H]^+^ 492.2345; found 492.2352. The purity of the compound was further confirmed by RP-HPLC: *R*_t_ = 19.99 min (96%; Figure S72).

#### 3.2.29. 2-[(1-Benzylpiperidin-4-yl)methyl]-6-[(4-bromo-2-fluorobenzyl)oxy-5-methoxy-2,3-dihydroinden-1-one (**8t**)

A solution of compound **7** (50 mg, 0.14 mmol) and K_2_CO_3_ (38 mg, 0.27 mmol) in anhydrous DMF (5 mL) was treated with 4-bromo-2-fluorobenzyl bromide (44 mg, 0.16 mmol), and the resulting mixture was stirred at RT overnight. The reaction mixture was then diluted with H_2_O, and extracted with EtOAc (3×). The combined organic layers were washed with H_2_O (3×) and brine (3×), dried over anhydrous MgSO_4_, and filtered. The filtrate was concentrated under reduced pressure and purified by column chromatography (SiO_2_ gel, pure CH_2_Cl_2_ to CH_2_Cl_2_:MeOH/19:1; R_f_ 0.56 in CH_2_Cl_2_:MeOH/19:1) to yield compound **8t** (69 mg, 91%) as an off-white solid: ^1^H-NMR (400 MHz, CDCl_3_, Figure S73) δ 7.37 (t, *J* = 7.6 Hz, 1H, aromatic), 7.31–7.26 (m, 6H, aromatic), 7.24 (m, 1H, aromatic), 7.19 (s, 1H, aromatic), 6.86 (s, 1H, aromatic), 5.12 (s, 2H, OCH_2_Ph), 3.93 (s, 3H, OCH_3_), 3.51 (s, 2H, NCH_2_Ph), 3.22 (dd, *J*_1_ = 17.6 Hz, *J*_2_ = 8.0 Hz, 1H), 2.90 (m, 2H), 2.68 (dt, *J*_1_ = 14.4 Hz, *J*_2_ = 3.2 Hz, 2H), 1.98 (m, 2H), 1.88 (m, 1H), 1.69 (m, 2H), 1.48 (m, 1H), 1.40–1.24 (m, 3H); ^13^C-NMR (100 MHz, CDCl_3_, Figure S74) δ 207.5, 161.4, 158.8, 155.9, 149.4, 148.0, 138.1, 130.63, 130.58, 129.3 (two carbons), 129.2, 128.1 (two carbons), 127.64, 127.60, 127.0, 123.0, 122.8, 122.2, 122.1, 119.2, 119.0, 107.8, 106.6, 64.3, 64.2, 63.3, 56.2, 53.70, 53.68, 45.4, 38.6, 34.3, 33.4, 32.9, 31.7; *m/z* calcd. for C_30_H_32_BrFNO_3_^+^ [M + H]^+^ 552.1544; found 552.1546. The purity of the compound was further confirmed by RP-HPLC: *R*_t_ = 21.25 min (96%; Figure S75).

#### 3.2.30. 2-[(1-Benzylpiperidin-4-yl)methyl]-6-[(2,4,6-trifluorobenzyl)oxy-5-methoxy-2,3-dihydroinden-1-one (**8u**)

A solution of compound **7** (50 mg, 0.14 mmol) and K_2_CO_3_ (38 mg, 0.27 mmol) in anhydrous DMF (5 mL) was treated with 2,4,6-trifluorobenzyl bromide (22 μL, 0.16 mmol) and the resulting mixture was stirred at RT overnight. The reaction mixture was then diluted with H_2_O, and extracted with EtOAc (3×). The combined organic layers were washed with H_2_O (3×) and brine (3×), dried over anhydrous MgSO_4_, and filtered. After standing at RT overnight, white solids precipitated out, which were filtered off. The filtrate was further concentrated under reduced pressure and purified by column chromatography (SiO_2_ gel, pure CH_2_Cl_2_ to CH_2_Cl_2_:MeOH/19:1; R_f_ 0.31 in CH_2_Cl_2_:MeOH/19:1) to yield compound **8u** (65 mg, 93%) as an off-white solid: ^1^H-NMR (400 MHz, CDCl_3_, Figure S76) δ 7.32-7.29 (m, 5H, aromatic), 7.24 (s, 1H, aromatic), 6.84 (s, 1H, aromatic), 6.68 (t, *J* = 8.4 Hz, 2H, aromatic), 5.08 (s, 2H, OCH_2_Ph), 3.89 (s, 3H, OCH_3_), 3.54 (s, 2H, NCH_2_Ph), 3.22 (dd, *J*_1_ = 17.6 Hz, *J*_2_ = 8.0 Hz, 1H), 2.92 (m, 2H), 2.68 (dt, *J*_1_ = 14.4 Hz, *J*_2_ = 3.2 Hz, 2H), 2.02 (m, 2H), 1.89 (m, 1H), 1.70 (m, 2H), 1.51 (m, 1H), 1.40–1.26 (m, 3H); 13C-NMR (100 MHz, CDCl_3_, Figure S77) δ 207.6, 164.6, 164.5, 164.3, 163.7, 163.6, 163.5, 163.4, 162.1, 162.0, 161.8, 161.1, 161.04, 161.00, 160.9, 152.2, 149.6, 148.2, 137.7, 129.4 (two carbons), 129.2, 128.2 (two carbons), 127.1, 108.92, 108.87, 108.72, 108.67, 108.53, 108.48, 107.9, 107.4, 100.69, 100.67, 100.6, 100.5, 100.44, 100.42, 100.39, 100.35, 100.2, 100.1, 63.2, 58.80, 58.76, 58.7, 56.2, 53.63. 53.59, 45.4, 38.6, 34.2, 33.4, 32.7, 31.6; *m/z* calcd. for C_30_H_31_F_3_NO_3_^+^ [M + H]^+^ 510.2251; found 510.2255. The purity of the compound was further confirmed by RP-HPLC: *R*_t_ = 20.30 min (95%; Figure S78).

#### 3.2.31. 2-[(1-Benzylpiperidin-4-yl)methyl]-6-[(2,3,4,5,6-pentafluorobenzyl)oxy-5-methoxy-2,3-dihydroinden-1-one (**8v**)

A solution of compound **7** (50 mg, 0.14 mmol) and K_2_CO_3_ (38 mg, 0.27 mmol) in anhydrous DMF (5 mL) was treated with 2,3,4,5-pentafluorobenzyl bromide (25 μL, 0.16 mmol), and the resulting mixture was stirred at RT overnight. The reaction mixture was then diluted with H_2_O, and extracted with EtOAc (3×). The combined organic layers were washed with H_2_O (3×) and brine (3×), dried over anhydrous MgSO_4_, and filtered. The filtrate was concentrated under reduced pressure and purified by column chromatography (SiO_2_ gel, pure CH_2_Cl_2_ to CH_2_Cl_2_:MeOH/19:1; R_f_ 0.34 in CH_2_Cl_2_:MeOH/19:1) to yield compound **8v** (61 mg, 81%) as an off-white solid: ^1^H-NMR (400 MHz, CDCl_3_, Figure S79) δ 7.32–7.24 (m, 6H, aromatic), 6.86 (s, 1H, aromatic), 5.13 (s, 2H, OCH_2_Ph), 3.90 (s, 3H, OCH_3_), 3.53 (s, 2H, NCH_2_Ph), 3.23 (dd, *J*_1_ = 17.6 Hz, *J*_2_ = 8.0 Hz, 1H), 2.91 (m, 2H), 2.69 (dt, *J*_1_ = 13.6 Hz, *J*_2_ = 4.0 Hz, 2H), 2.00 (m, 2H), 1.89 (m, 1H), 1.70 (m, 2H), 1.51 (m, 1H), 1.40–1.24 (m, 3H); 13C-NMR (100 MHz, CDCl_3_, Figure S80) δ 207.4, 156.2, 150.1, 147.8, 147.20, 147.16, 147.12, 147.08, 147.04, 147.01, 146.97, 146.93, 144.70, 144.66, 144.62, 144.58, 144.55, 144.51, 144.47, 144.43, 143.23, 143.16, 143.10, 143.05, 142.97, 140.7, 140.61, 140.56, 140.5, 140.4, 138.93, 138.90, 138.8, 138.7, 138.65, 138.60, 138.59, 137.6, 136.42, 136.38, 136.30, 136.26, 136.23, 136.13, 136.10, 129.4 (two carbons), 129.2, 128.5, 128.2 (two carbons), 127.1, 109.91, 109.87, 109.74, 109.70, 109.6, 109.5, 108.0, 107.8, 63.1, 58.7, 56.2, 53.6, 53.5, 45.3, 38.6, 34.2, 33.4, 32.6, 31.5; *m/z* calcd. for C_30_H_29_F_5_NO_3_^+^ [M + H]^+^ 546.2062; found 546.2058. The purity of the compound was further confirmed by RP-HPLC: *R*_t_ = 20.84 min (95%; Figure S81).

### 3.3. In Vitro Cholinesterase (ChE) Inhibition Assays

Experiments were performed as previously described [11,13]. Briefly, donepezil analogues (102 pm to 200 μM) were dissolved in sodium phosphate buffer ((100 μL), 0.1 M, pH 8.0) (Buffer A) and subjected to a 5-fold serial dilution. ChE (either *Ee*AChE or *Ef*BChE) was added to the solution of inhibitors (50 μL, containing 0.08 U/mL ChE (final concentration for both *Ee*AChE and *Ef*BChE) in Buffer A. The mixture of inhibitor and enzyme was incubated for 10 min before initiation with DTNB (50 μL, 0.25 mM final concentration) and acylthiocholine (acetylthiocholine for *Ee*AChE and butyrylthiochholine for *Ef*BChE) (0.5 mM final concentration) in phosphate buffer. The reaction was monitored at 412 nm taking measurements every 30 s for 10 min using a Spectra Max M5 plate reader (Molecular Devices, San Jose, CA, USA) at 25 °C. Data was corrected with the negative control (no acylthiocholine), and normalized to the positive control (no inhibitor) using the initial rates (first 5 min). All assays were performed in duplicate or triplicate. *Hs*AChE was treated in the same manner with the following exceptions: the final concentration of *Hs*AChE was 0.16 μg/mL (~0.16 U/mL), and reactions were performed at 37 °C. The data was fitted to a sigmoidal curve, and IC_50_ values were calculated using Sigmaplot 14.0 (Systat Software, San Jose, CA, USA). The IC_50_ curves for *Ee*AChE and *Ef*BChE inhibition are presented in Table 1 and Figures S82 and S83 (for *Ee*AChE) and Figures S84 and S85 (for *Ef*BChE). The IC_50_ curves for *Hs*AChE inhibition are presented in Table 2 and Figure S86.

### 3.4. BACE1 Inhibition

Inhibition of BACE1 was tested using the commercial kit (cat CS0010-1KT, Millipore-Sigma, St. Louis, MO, USA) following the directions accompanying the kit. All compounds were tested in duplicate at a single concentration (200 μM) in order to confirm any activity. All compounds that showed BACE1 inhibitory activity were then tested in a concentration-dependent manner. Dilutions were originally performed in DMSO, and 2 μL added to the reaction in order to account for any moderation of activity from the vehicle. Fluorescent measurements were taken after 2 h. The resulting rates were normalized to the reaction without inhibitor. In order to get an appropriate sigmoidal fit, two additional points (400 and 1000 μM) were added to the data when needed. Since the activity of the enzyme was already negligible at 200 μM, these points aid the sigmoidal nature of the curve fit. These data are presented in Table 3 and Figure S87.

### 3.5. Molecular Docking of Donepezil and Compound 8l with BACE1

To further validate the biochemical results obtained against BACE1, we modeled donepezil and compound **8l** using a known crystal structure of BACE1 with an inhibitor, sharing the vicinyl dioxygen substitution of donepezil as a model (PDB# 4FM7 [25]). Swiss Dock [29,30] was used to identify the potential binding sites of donepezil or compound **8l** with the crystal structure. Once docking calculations were completed, Chimera [31] was used to compare the potential binding sites with that of the known inhibitors. The closest alignments were selected, and they are presented in Figure 1.

## 4. Conclusions

We have synthesized 22 new donepezil analogues, **8a**–**v**, and evaluated their biochemical capabilities, along with that of the parent donepezil and its 6-*O*-desmethyl adduct **7**. Without exception, these compounds were all able to inhibit the action of *Ee*AChE and *Ef*BChE in the low-to-sub-micromolar ranges. Compound **8t**, one of the better inhibitors of *Ee*AChE and *Ef*BChE was also a very efficient inhibitor of *Hs*AChE showing the highest preference for this medically relevant enzyme. Attachment of an alkyl/aromatic group at the 6-*O*-position of the indanone ring also seems to enhance their efficacy. While their inhibitory capabilities were greater against *Ee*AChE than *Ef*BChE, the donepezil analogues **8h**–**v** with aromatic substituents displayed a much improved potency when compared to donepezil against *Ef*BChE than *Ee*AChE. The analogues **8a**–**g** with alkyl substituents showed proportional change with respect to donepezil against both *Ee*AChE and *Ef*BChE. The donepezil analogues **8c**, **8e**, **8f**, and **8l** also displayed potent BACE1 inhibitory activities, and thus appeared to be multifunctional compounds for the treatment of Alzheimer’s disease.

## Figures and Tables

**Table 1:** Inhibition (IC_50_ values in µM, with standard error) of the activity of *Ee*AChE (from *Electrophorus electricus*) and BChE (from *Equus ferus*) by donepezil and its analogues **7** and **8a**–**v**, and the selectivity index (SI) for each inhibitor based on IC_50_ values.

Cpd	*Ee*AChE	*Ef*BChE	SI
Donepezil HCl	0.12 ± 0.01	2.0 ± 0.1	17
**7**	0.41 ± 0.05	4.3 ± 0.4	11
**8a**	0.054 ± 0.003	0.57 ± 0.04	11
**8b**	0.021 ± 0.003	0.48 ± 0.03	23
**8c**	0.14 ± 0.02	2.1 ± 0.3	15
**8d**	0.059 ± 0.004	1.3 ± 0.1	22
**8e**	0.044 ± 0.003	1.3 ± 0.2	30
**8f**	0.061 ± 0.007	1.3 ± 0.2	21
**8g**	0.79 ± 0.28	5.2 ± 1.6	6.6
**8h**	0.13 ± 0.01	0.70 ± 0.05	5.4
**8i**	0.23 ± 0.03	1.0 ± 0.2	4.3
**8j**	0.13 ± 0.01	0.67 ± 0.17	5.2
**8k**	0.13 ± 0.02	0.46 ± 0.06	3.5
**8l**	0.071 ± 0.015	0.72 ± 0.10	10
**8m**	0.081 ± 0.005	0.57 ± 0.10	7.0
**8n**	0.16 ± 0.02	0.96 ± 0.15	6.0
**8o**	0.12 ± 0.02	0.76 ± 0.12	6.3
**8p**	0.032 ± 0.010	0.25 ± 0.08	7.8
**8q**	0.11 ± 0.01	0.48 ± 0.08	4.4
**8r**	0.090 ± 0.009	0.60 ± 0.15	6.7
**8s**	0.016 ± 0.001	0.44 ± 0.05	28
**8t**	0.054 ± 0.007	0.37 ± 0.05	6.9
**8u**	0.027 ± 0.004	0.20 ± 0.03	7.4
**8v**	0.17 ± 0.02	0.11 ± 0.01	0.69

**Table 2:** Inhibition (IC_50_ values in μM) of the activity of *Hs*AChE by donepezil and its analogue **8t**.

Cpd	IC_50_ (μM)	SI ^a^
Donepezil HCl	0.032 ± 0.011	3.8
**8t**	0.0018 ± 0.0006	30

^a^ Selectivity index of *Ee*AChE *versus Hs*AChE, based on IC_50_ values.

**Table 3:** Inhibition (IC50 values in μM, unless otherwise noted) of the activity of BACE1 by donepezil and its analogues **7**, and **8a**–**v**. BACE inhibitor IV was used as a control.

Cpd	IC_50_ (μM)
Donepezil HCl	1.5 ± 0.3
**7**	--
**8a**	95 ± 12
**8b**	~100
**8c**	6.1 ± 0.1
**8d**	~100
**8e**	7.9 ± 0.9
**8f**	7.9 ± 2.4
**8g**	--
**8h**	58 ± 1
**8i**	58 ± 2
**8j**	--
**8k**	--
**8l**	3.4 ± 0.1
**8m**	~100
**8n**	12 ± 3
**8o**	21 ± 4
**8p**	34 ± 9
**8q**	37 ± 5
**8r**	~100
**8s**	30 ± 8
**8t**	169 ± 2
**8u**	91 ± 14
**8v**	29 ± 6
BACE1 inhibitor IV	0.63 ± 0.18 nM

## Supplementary Materials

The Supplementary Materials include ^1^H and ^13^C-NMR spectra for the molecules synthesized, as well as HPLC traces of compounds tested for activity (Figures S1–S81). The IC_50_ curves for the inhibition of *Ee*AChE, *Hs*AChE, *Ef*BChE, and BACE1 are also provided (Figures S82–S87). The SwissDock modeling is also provided (Figure S88). These materials are available free of charge via the internet.

## Abbreviations

Aβ	amyloid-β
APP	amyloid precursor protein
BACE	β-secretase
ChE	cholinesterase
*Ee*AChE	acetylcholinesterase (from *Electrophorus electricus*)
*Ef*BChE	butyrylcholinesterase (from *Equus ferus*)
*Hs*AChE	acetylcholinesterase (from *Homo sapiens*)
IC_50_	half maximal inhibitory concentration
KOH	potassium hydroxide
MsOH	methanesulfonic acid
TBDMS	*tert*-butyldimethylsilyl
