# Supplementary material for: Multifunctional Donepezil Analogues as Cholinesterase and BACE1 Inhibitors
Source: Molecules. 2018 Dec 8;23(12):3252. doi: 10.3390/molecules23123252 (PMC6321525; doi:10.3390/molecules23123252)
Supplement: Supplementary file 1 [file molecules-23-03252-s001.pdf]

Supplementary Materials

Multifunctional donepezil analogues as cholinesterase and BACE1 inhibitors

Keith D. Green,[a] Marina Y. Fosso,[a] Sylvie Garneau-Tsodikova[a,*]

[a] University of Kentucky, College of Pharmacy, Department of Pharmaceutical Sciences, Lexington, KY, USA, 40536-0596.

*sylviegtsodikova@uky.edu

Table of Content **Page #**

Figures S1-S88 S1-S48

Figures S1-S88:

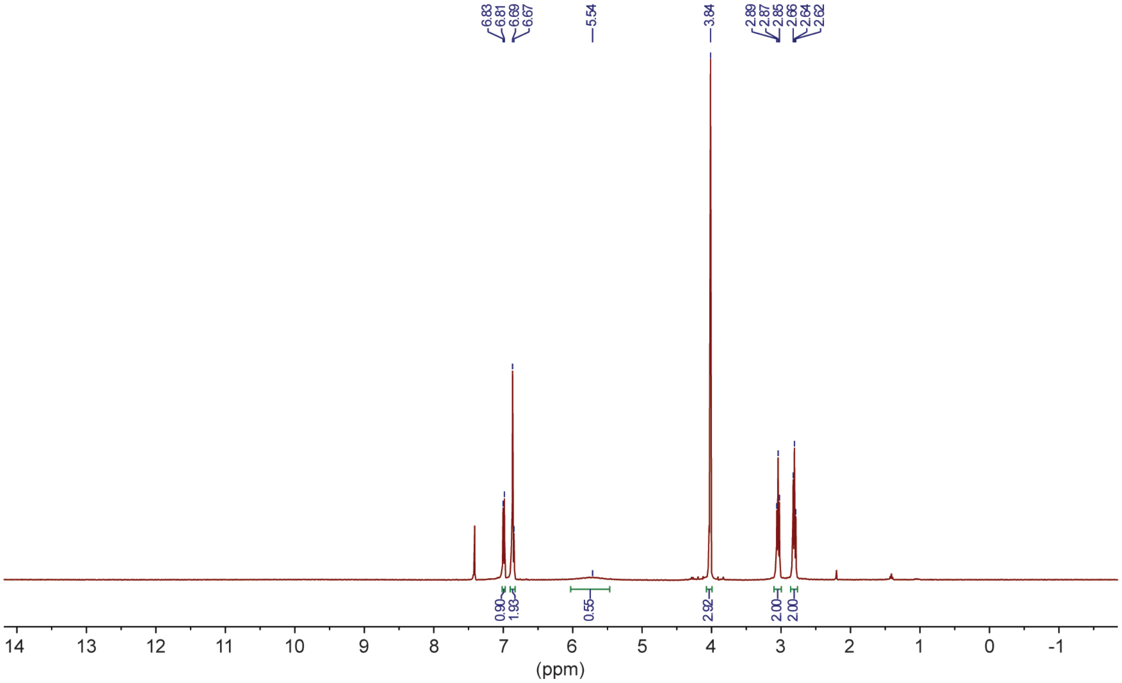

Fig. S1: ^{1}H NMR spectrum for compound **2** in $CDCl_3$ (400 MHz).

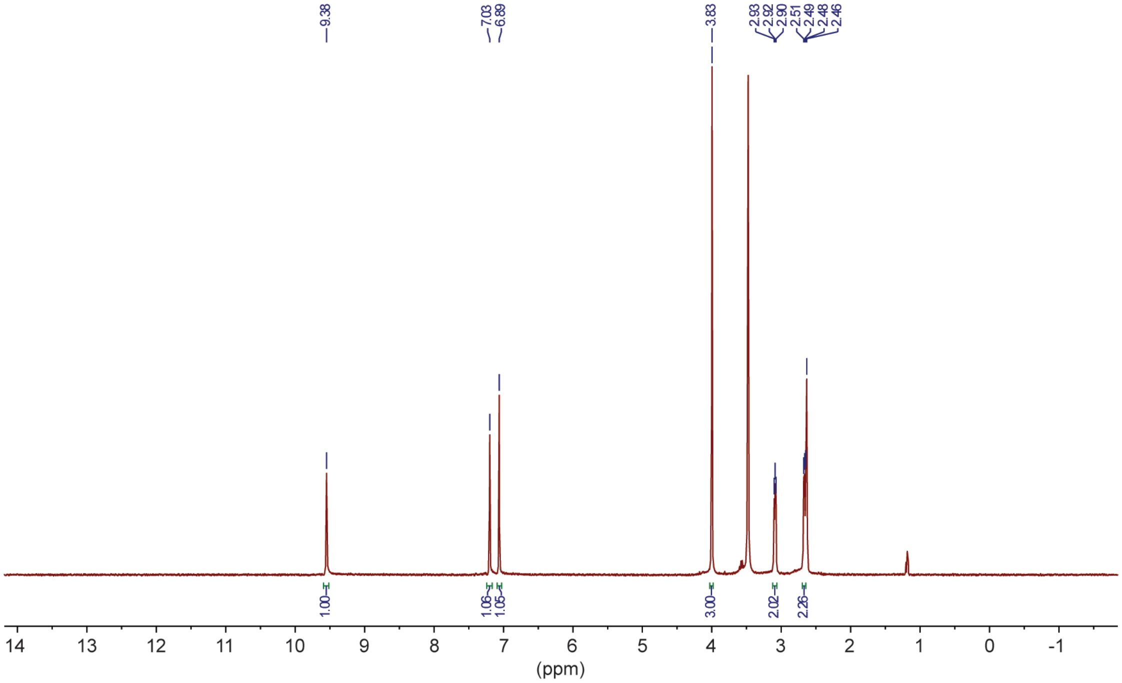

Fig. S2: ^{1}H NMR spectrum for compound **3** in $(CD_3)_2SO$ (400 MHz).

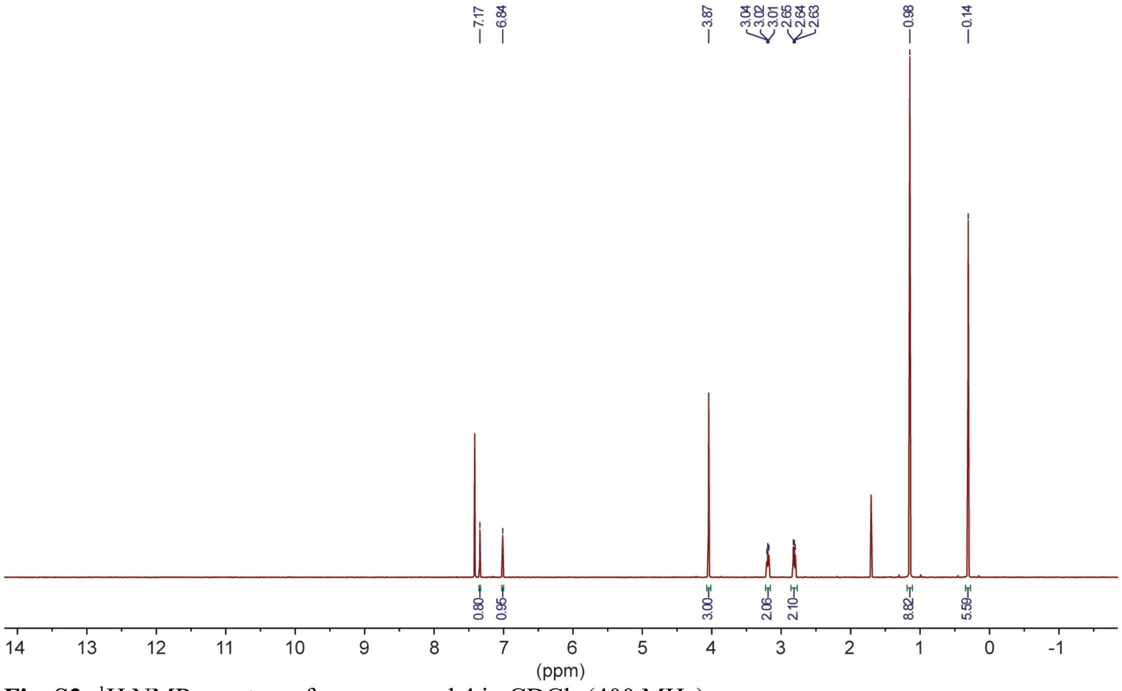

Fig. S3: ^{1}H NMR spectrum for compound **4** in $CDCl_3$ (400 MHz).

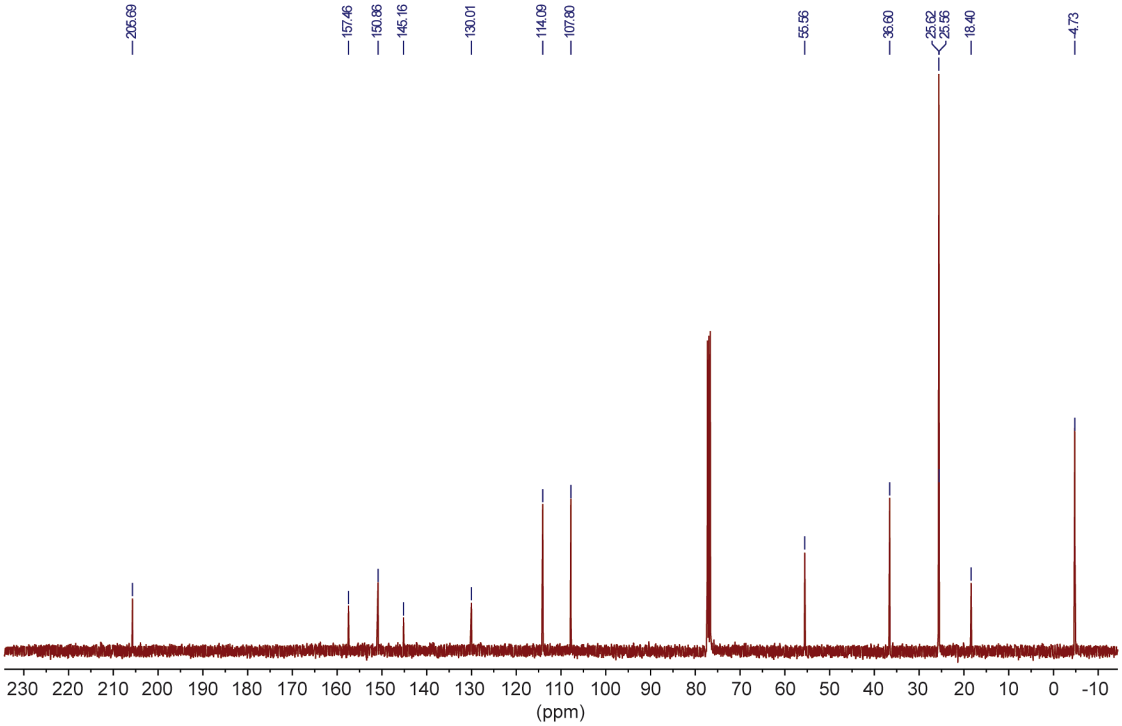

Fig. S4: ^{13}C NMR spectrum for compound **4** in $CDCl_3$ (100 MHz).

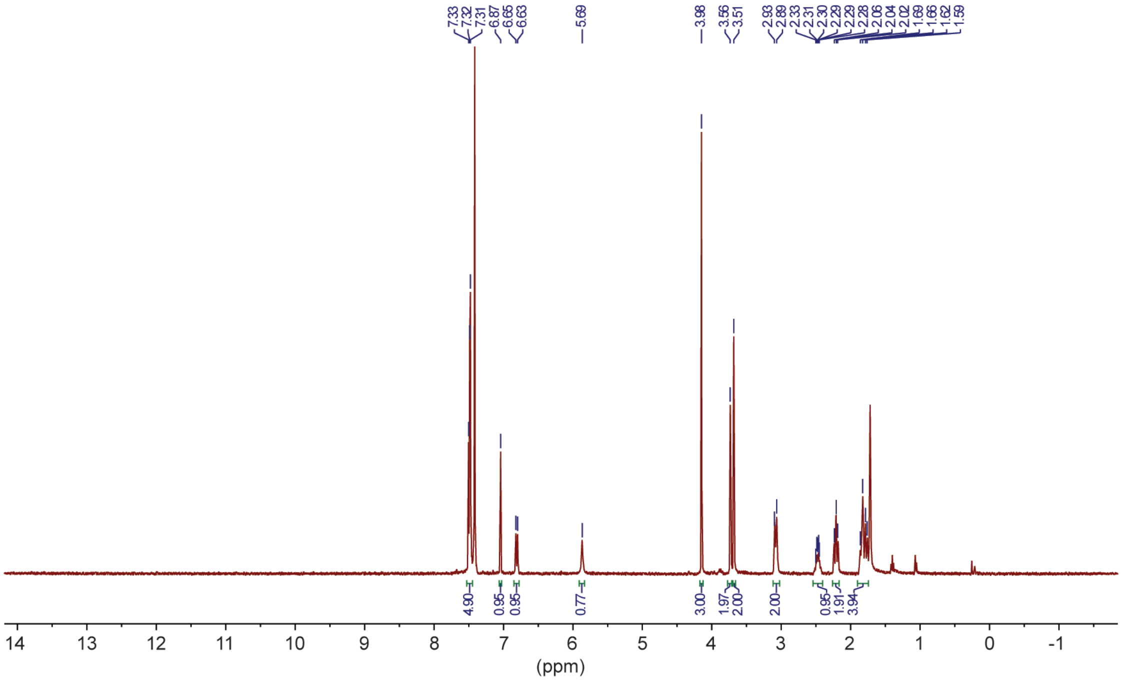

Fig. S5: ^{1}H NMR spectrum for compound **6** in $CDCl_3$ (400 MHz).

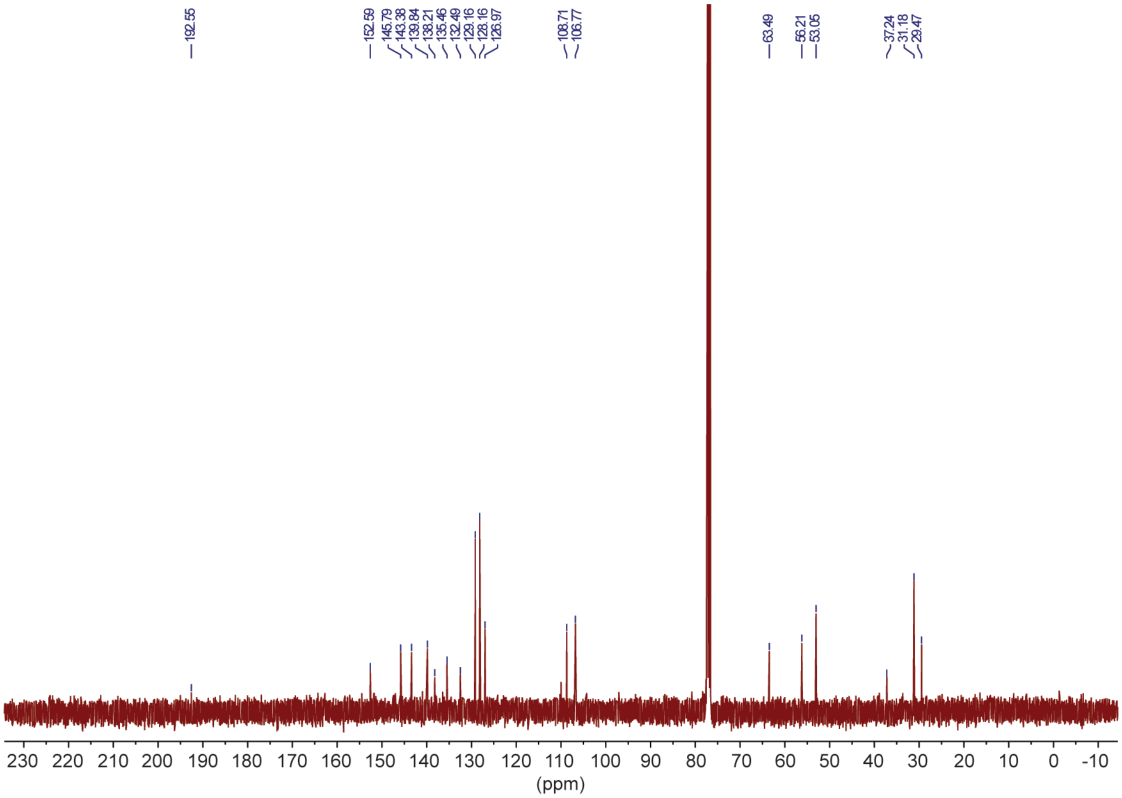

Fig. S6: ^{13}C NMR spectrum for compound **6** in $CDCl_3$ (100 MHz).

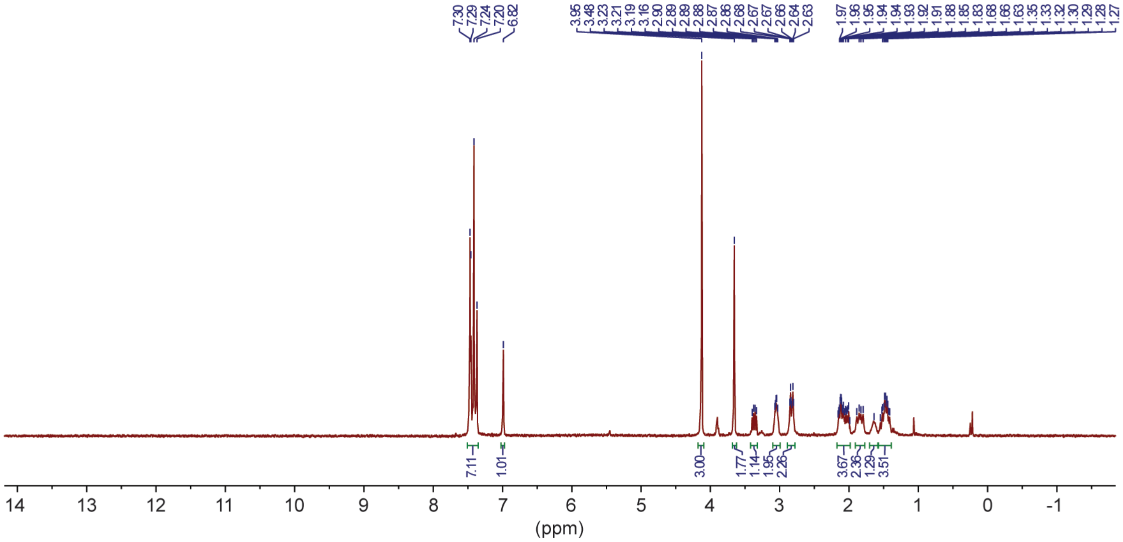

Fig. S7: ^{1}H NMR spectrum for compound **7** in $CDCl_3$ (400 MHz).

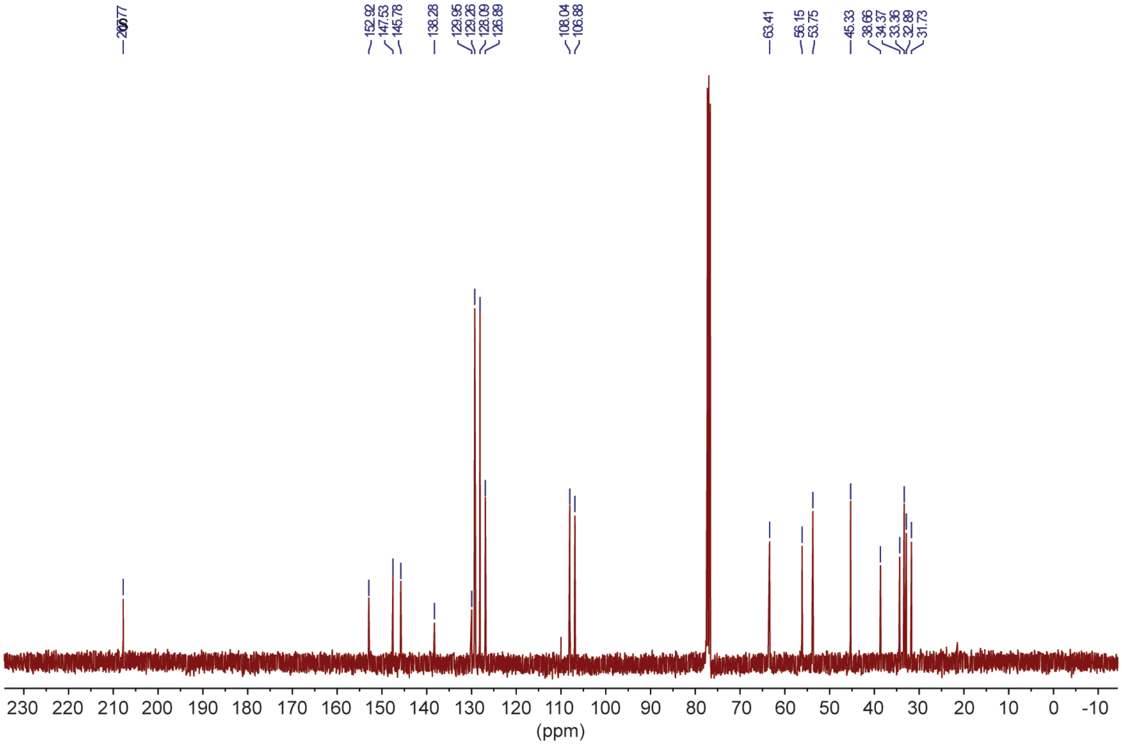

Fig. S8: ^{13}C NMR spectrum for compound **7** in $CDCl_3$ (100 MHz).

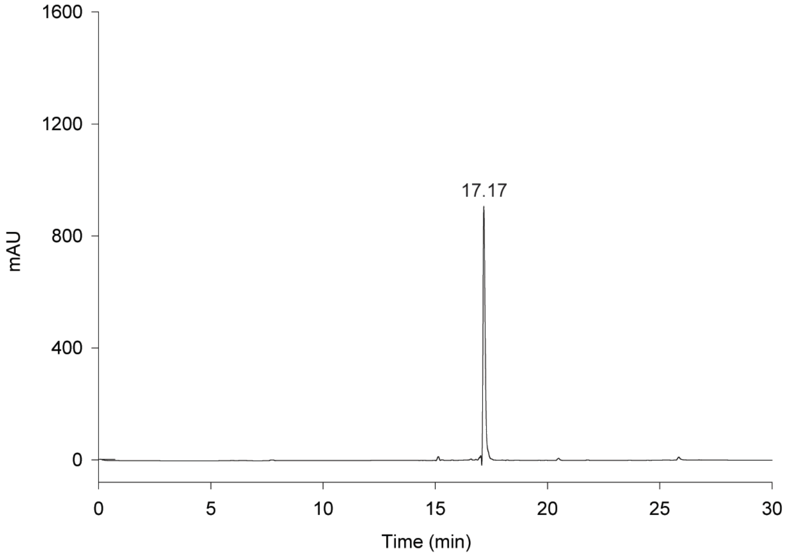

Fig. S9: HPLC trace for compound **7**. R_t = 17.17 min.

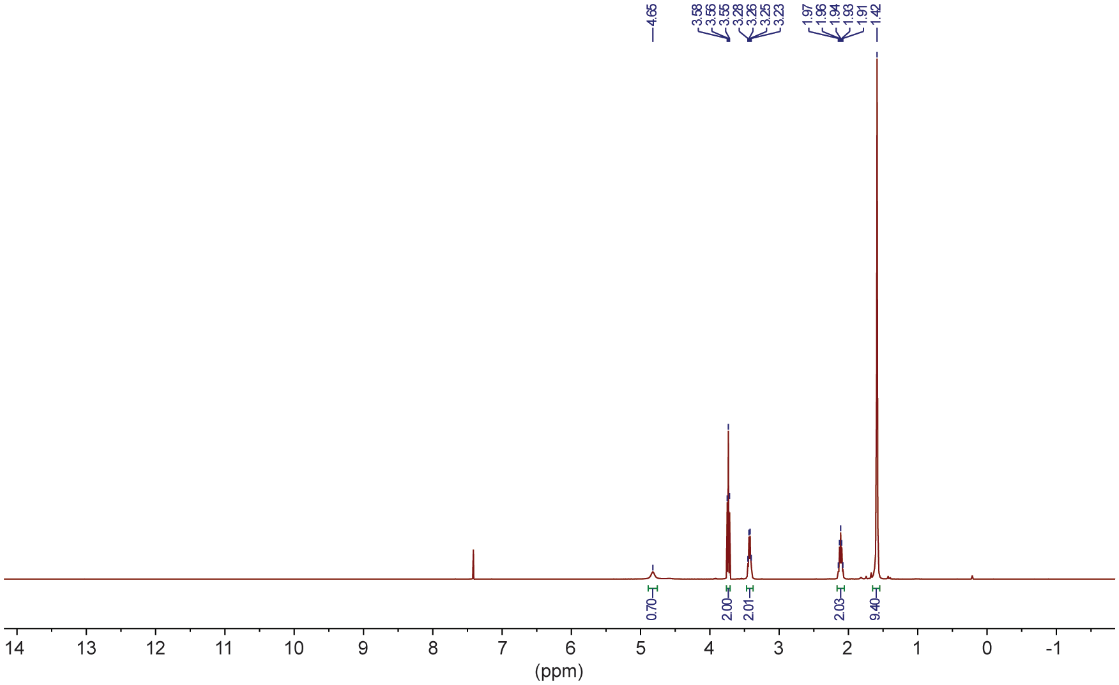

Fig. S10: ^{1}H NMR spectrum for *tert*-butyl *N*-(3-chloropropyl)carbamate in $CDCl_3$ (400 MHz).

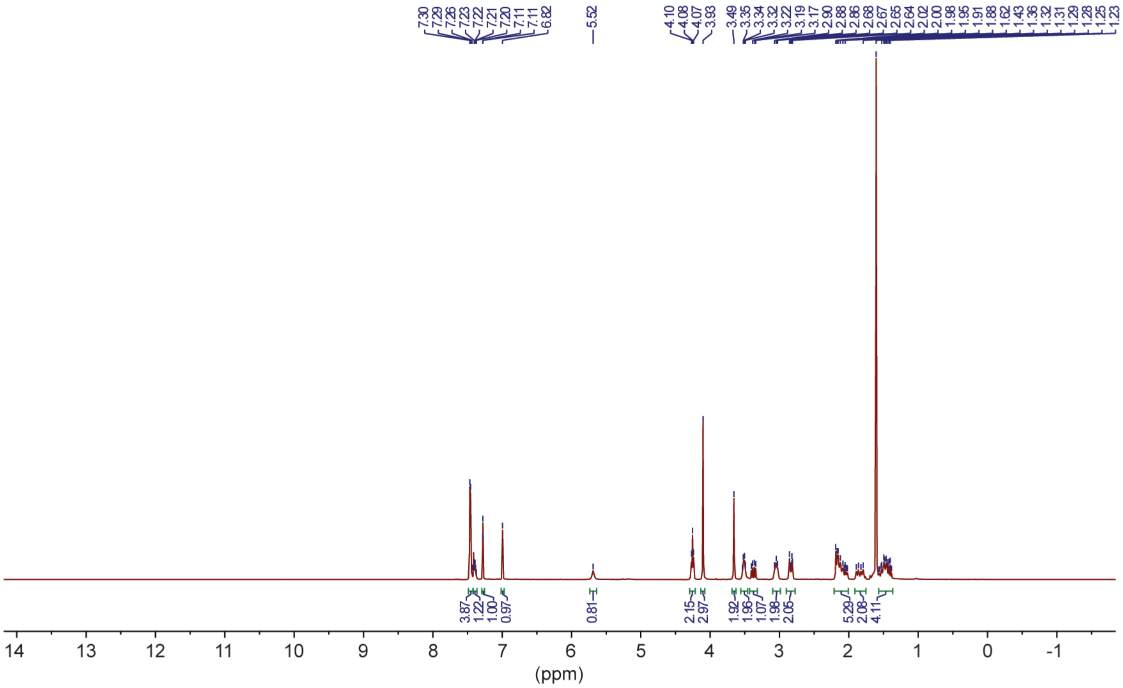

Fig. S11: ^{1}H NMR spectrum for Boc-protected compound **8a** in $CDCl_3$ (400 MHz).

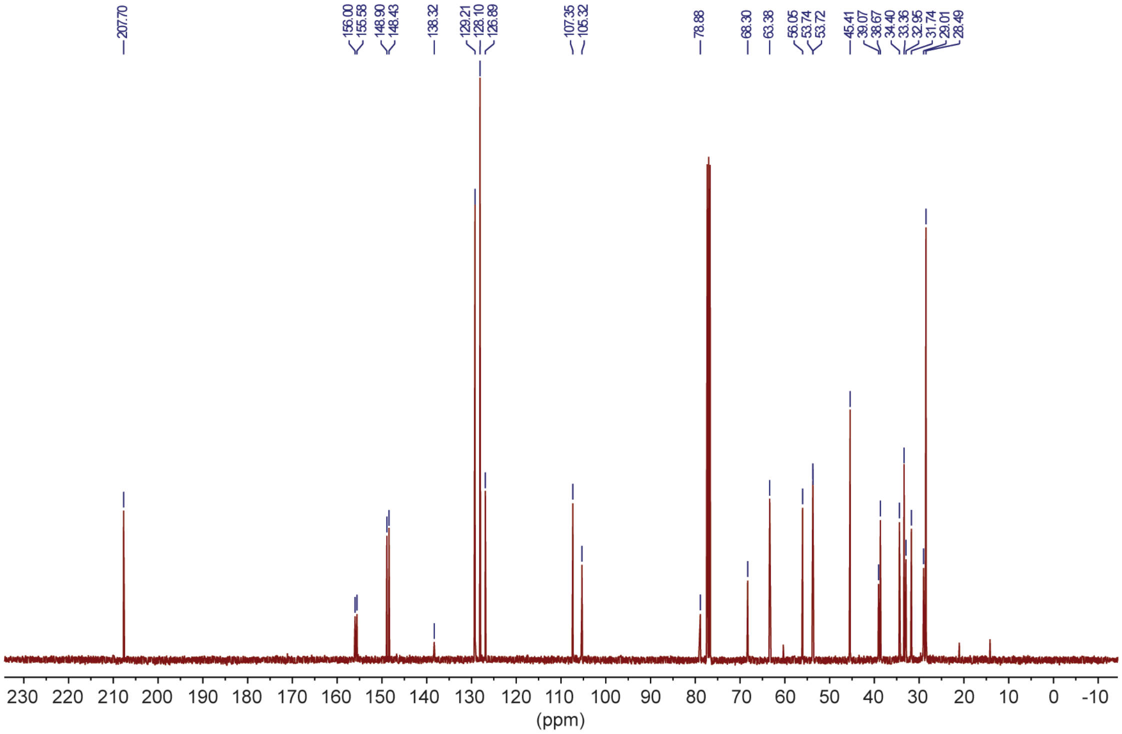

Fig. S12: ^{13}C NMR spectrum for Boc-protected compound **8a** in $CDCl_3$ (100 MHz).

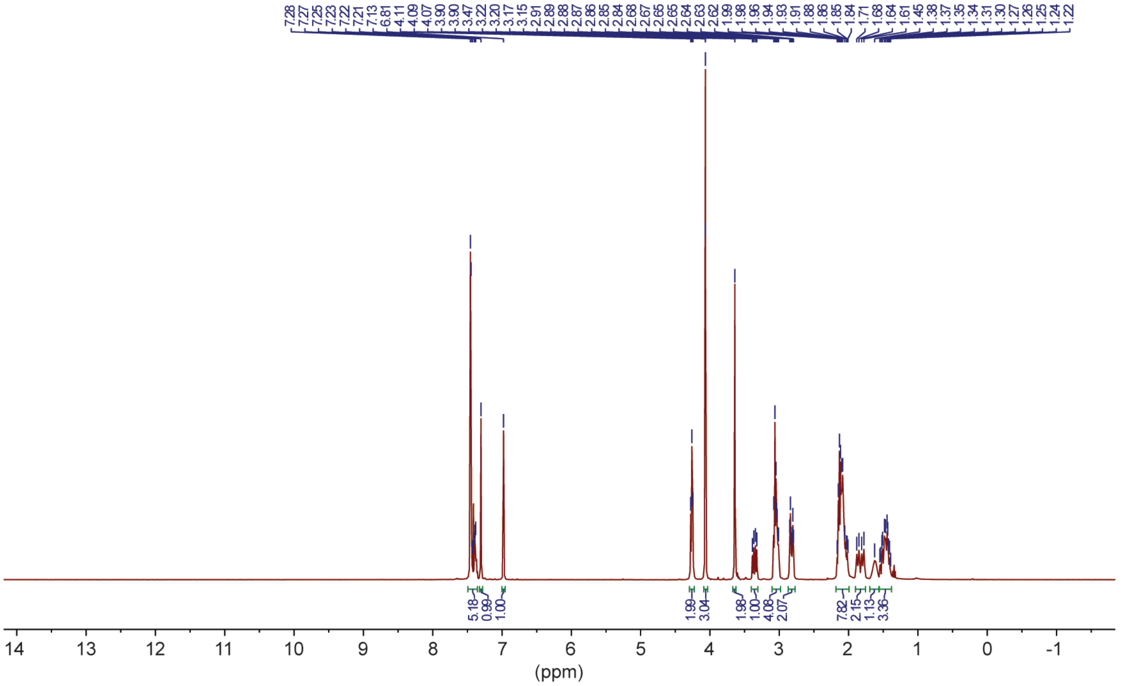

Fig. S13: ^{1}H NMR spectrum for compound **8a** in $CDCl_3$ (400 MHz).

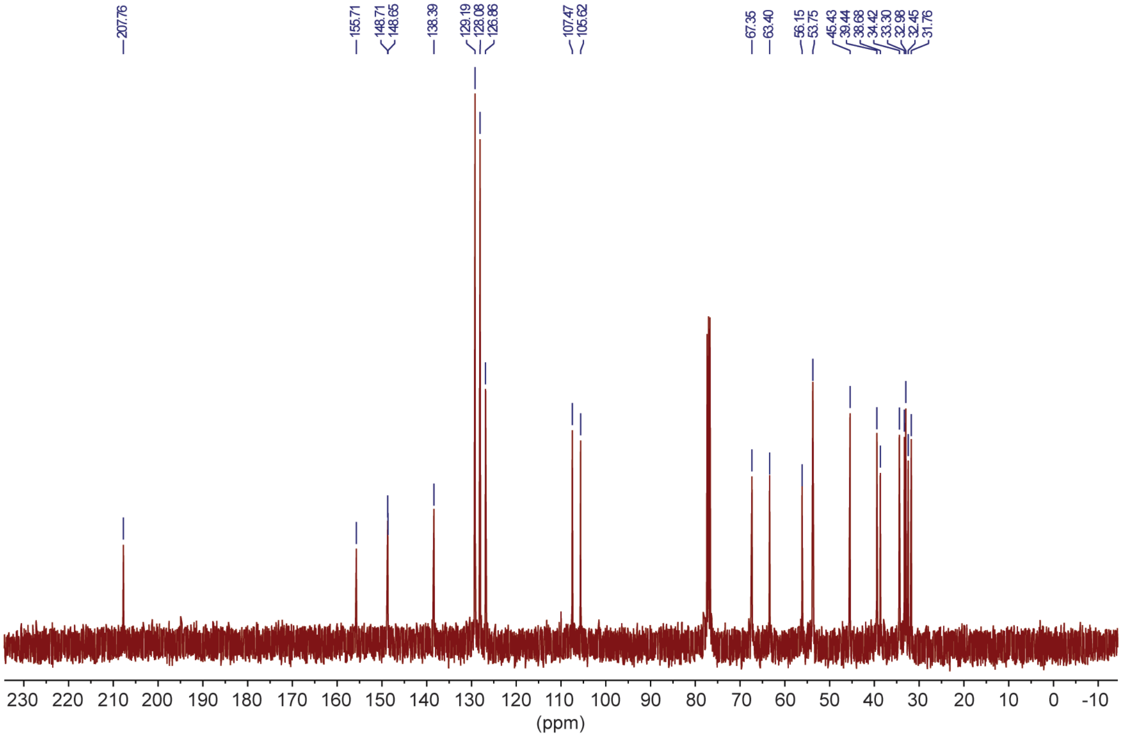

Fig. S14: ^{13}C NMR spectrum for compound **8a** in $CDCl_3$ (100 MHz).

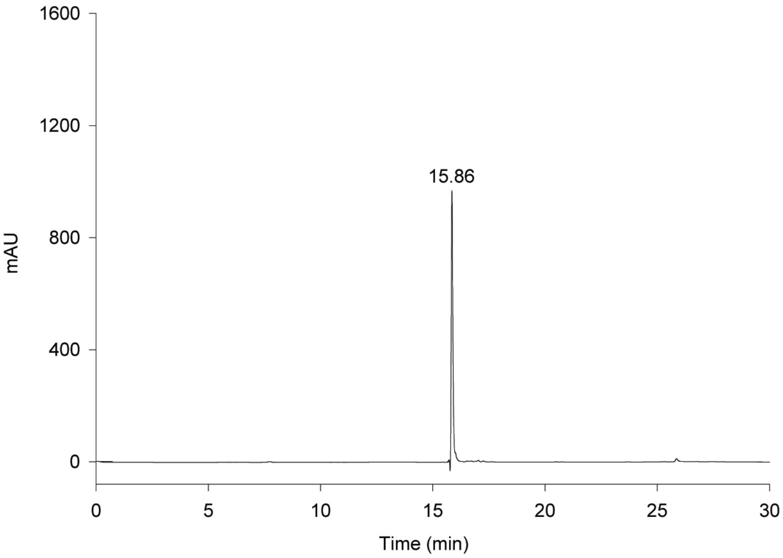

Fig. S15: HPLC trace for compound **8a**. R_t = 15.86 min.

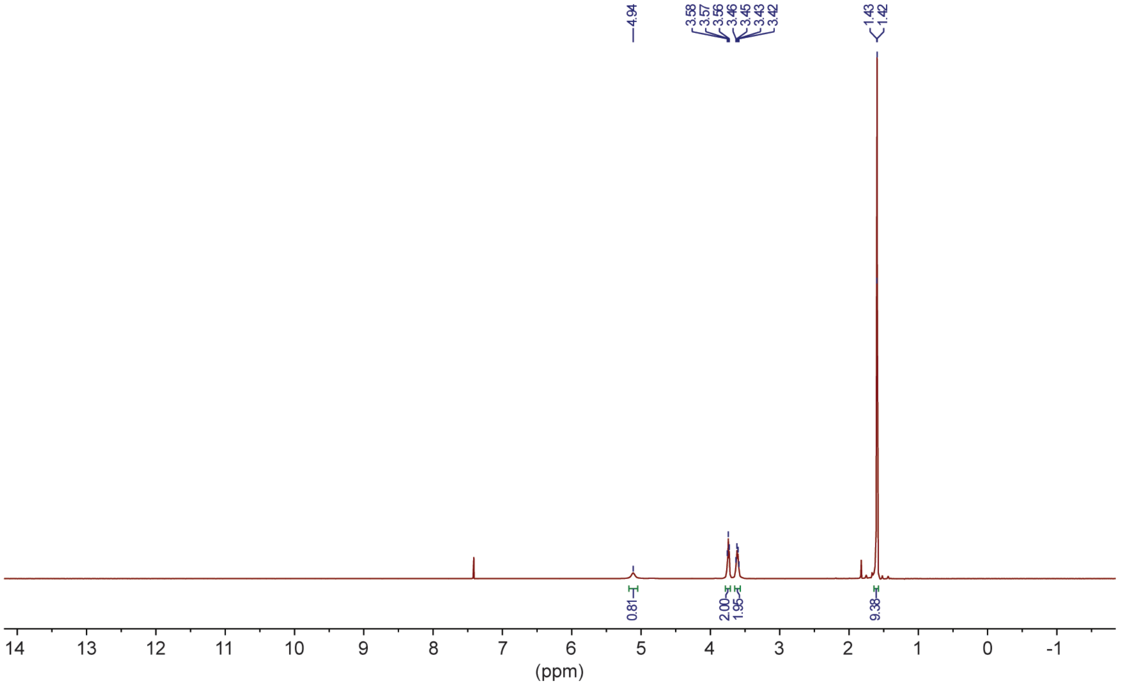

Fig. S16: ^{1}H NMR spectrum for *tert*-butyl *N*-(2-chloroethyl)carbamate in $CDCl_3$ (400 MHz).

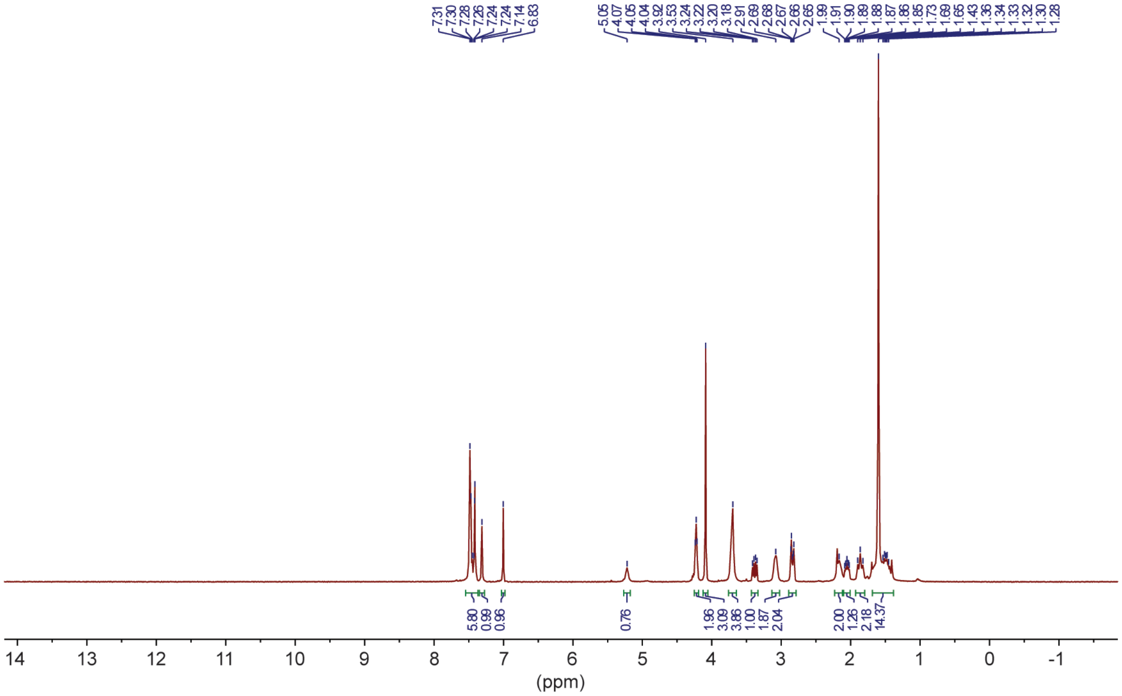

Fig. S17: ^{1}H NMR spectrum for Boc-protected compound **8b** in $CDCl_3$ (400 MHz).

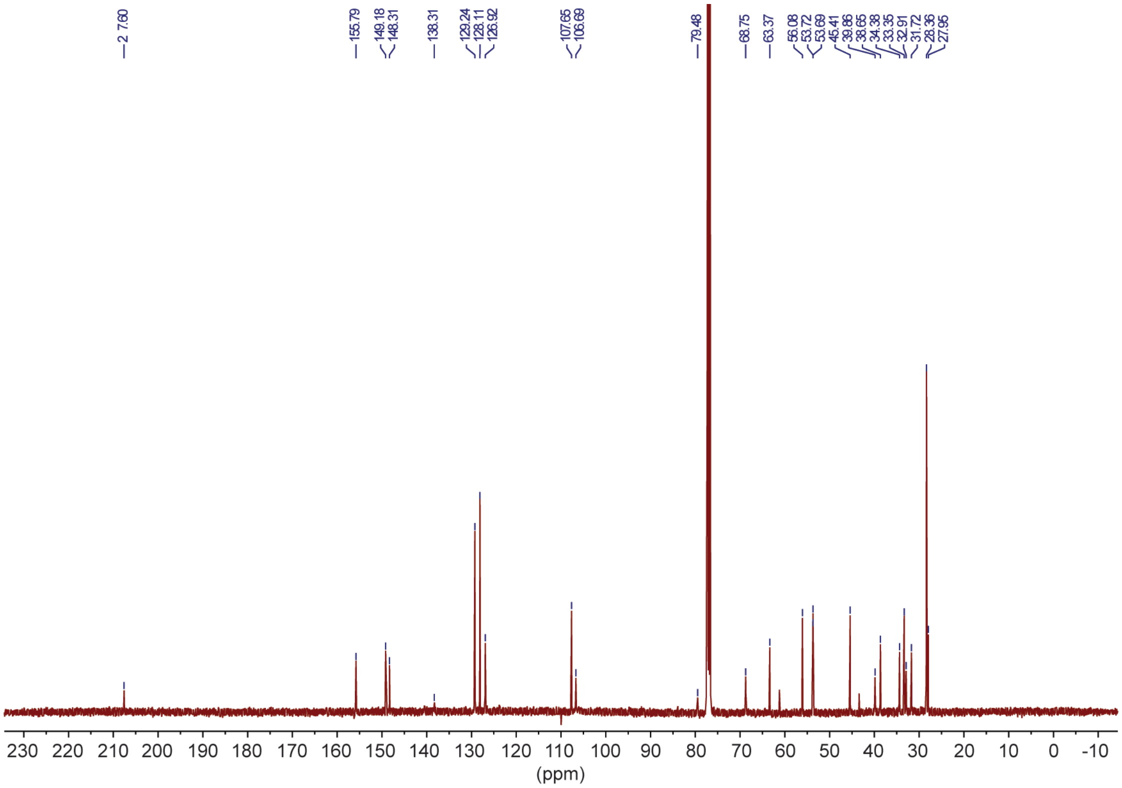

Fig. S18: ^{13}C NMR spectrum for Boc-protected compound **8b** in $CDCl_3$ (100 MHz).

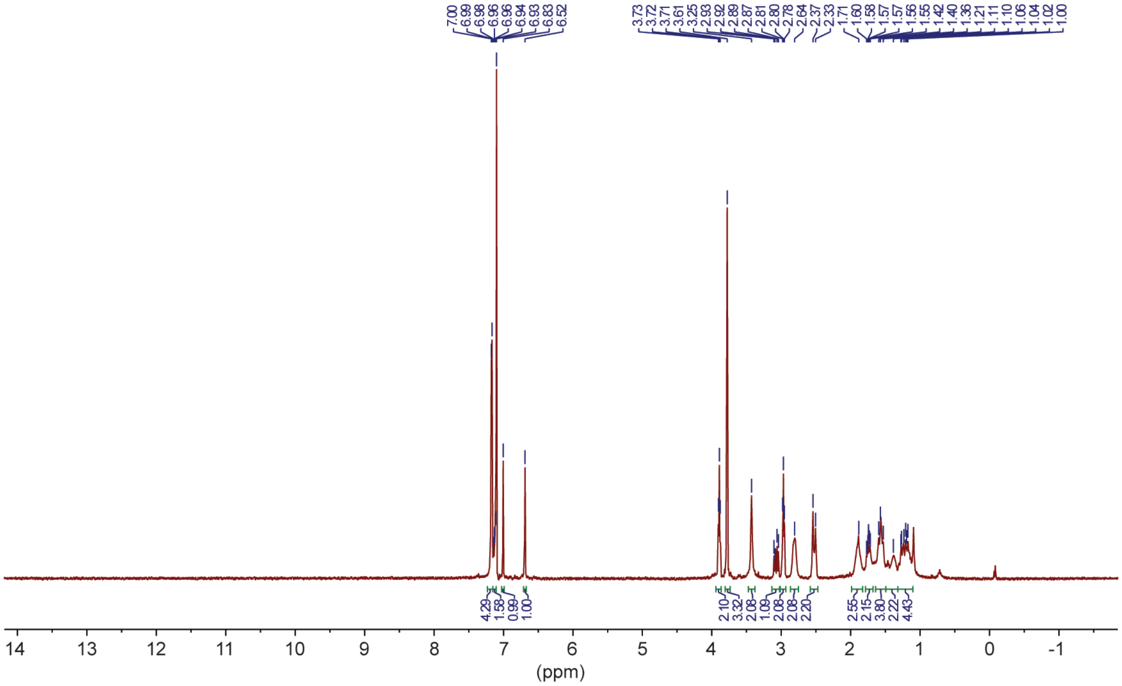

Fig. S19: ^{1}H NMR spectrum for compound **8b** in $CDCl_3$ (400 MHz).

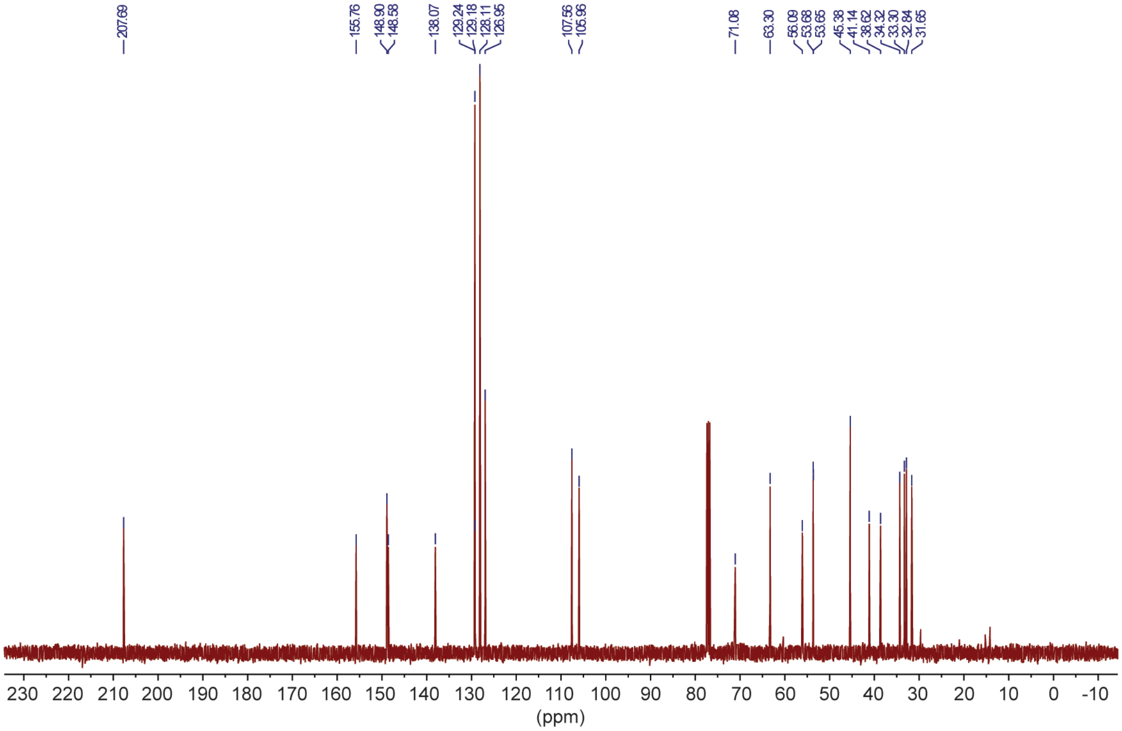

Fig. S20: ^{13}C NMR spectrum for compound **8b** in $CDCl_3$ (100 MHz).

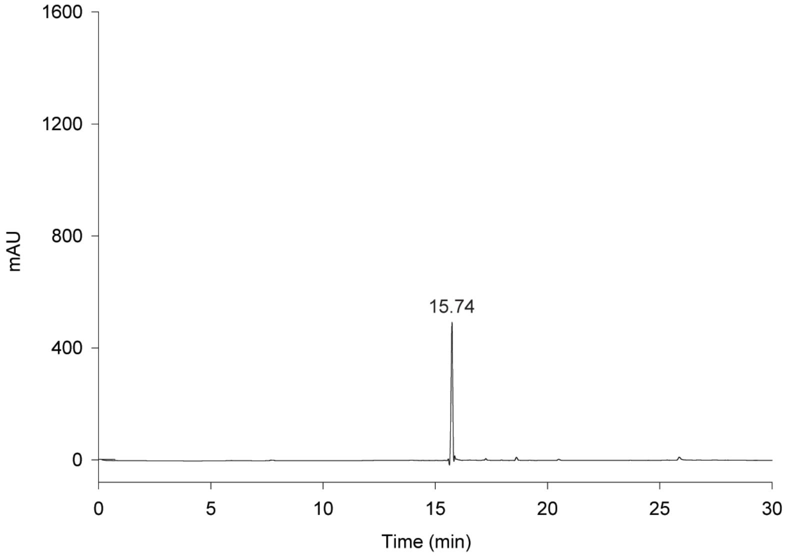

Fig. S21: HPLC trace for compound **8b**. R_t = 15.74 min.

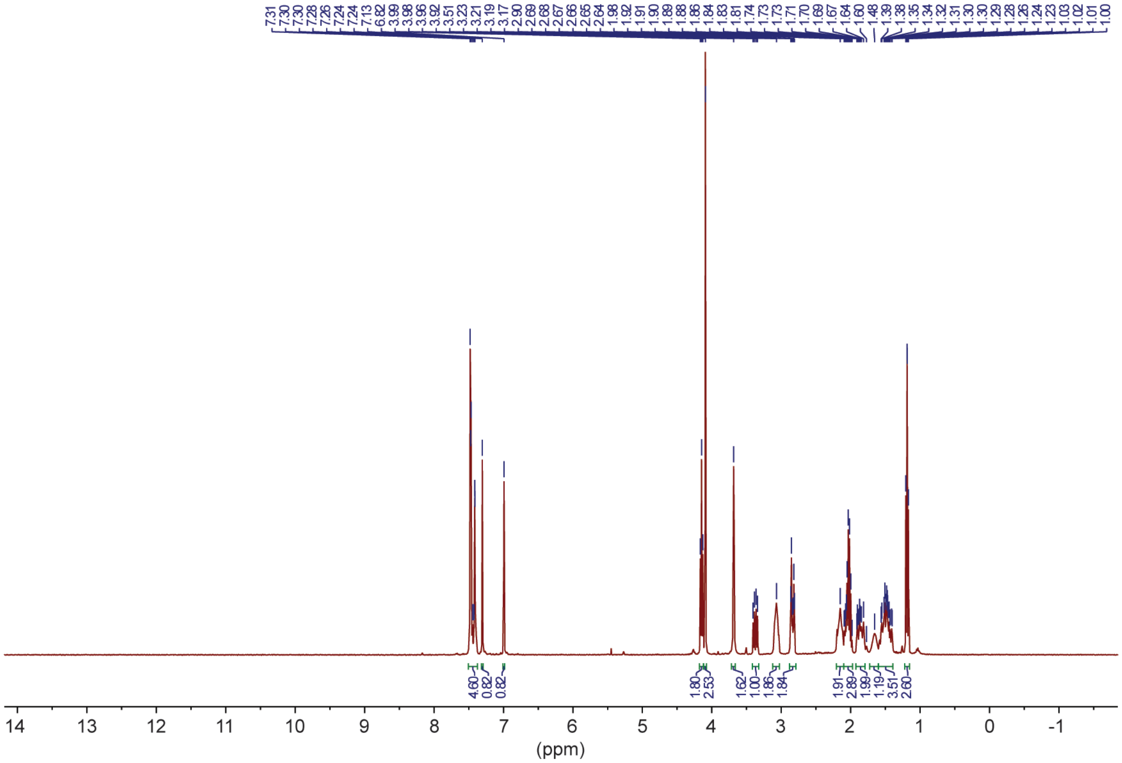

Fig. S22: ^{1}H NMR spectrum for compound **8c** in $CDCl_3$ (400 MHz).

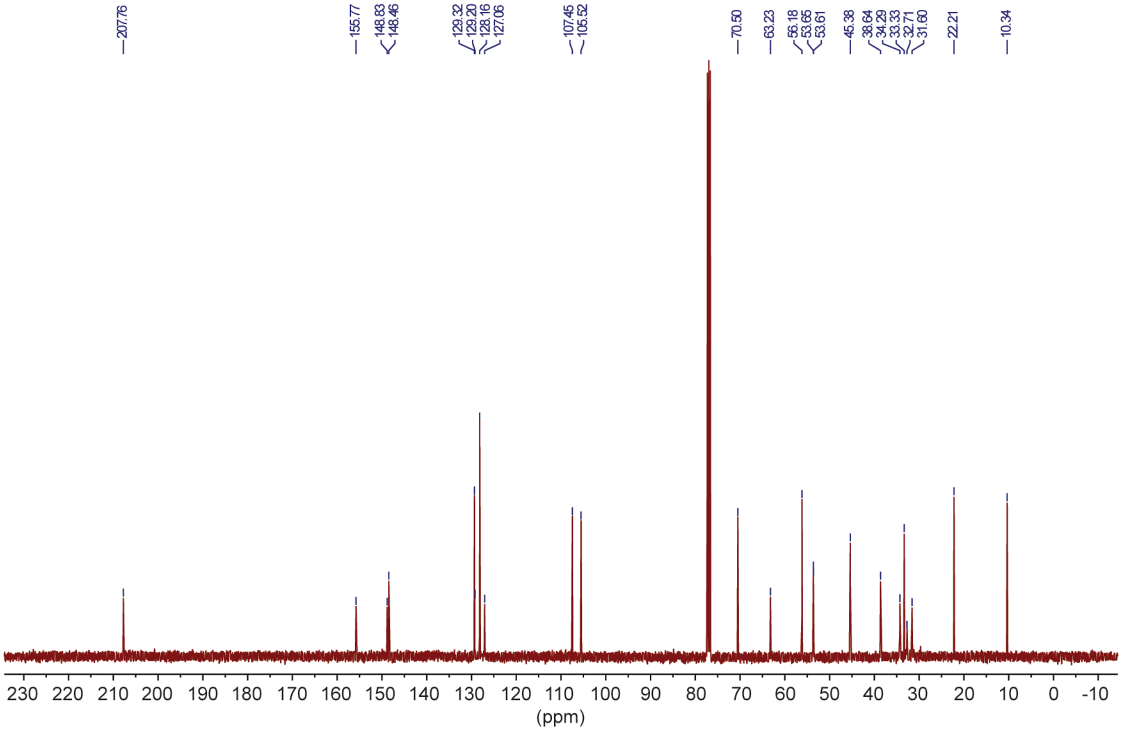

Fig. S23: ^{13}C NMR spectrum for compound **8c** in $CDCl_3$ (100 MHz).

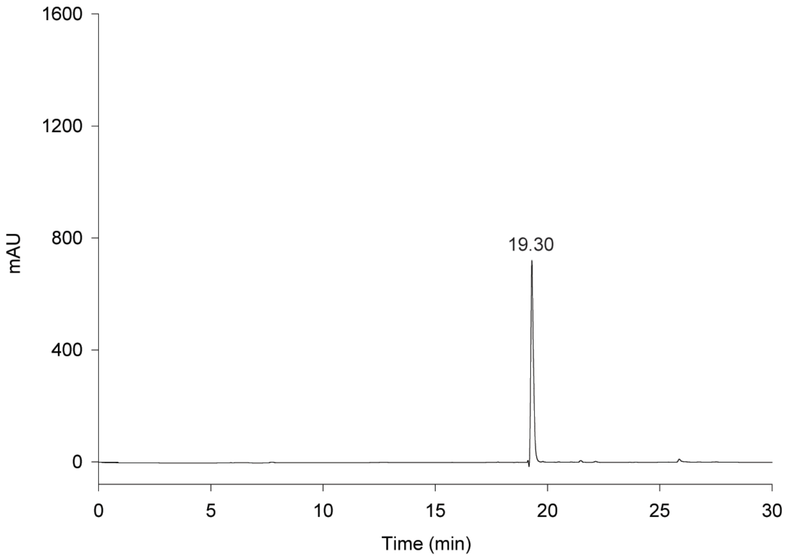

Fig. S24: HPLC trace for compound **8c**. R_t = 19.30 min.

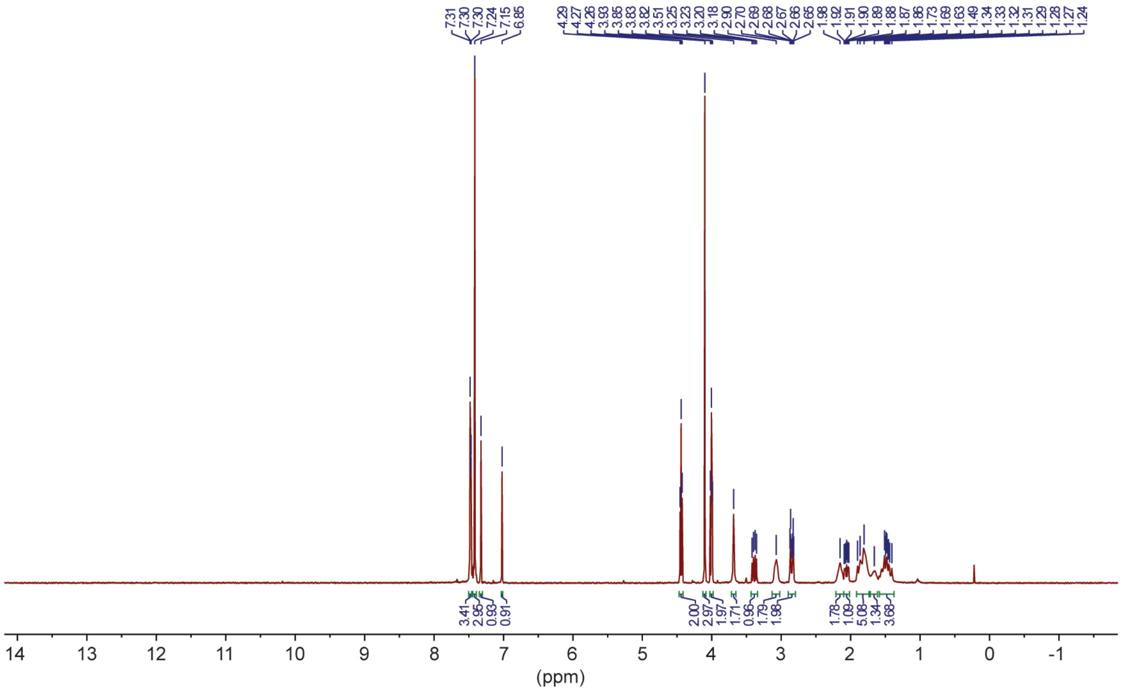

Fig. S25: ^{1}H NMR spectrum for compound **8d** in $CDCl_3$ (400 MHz).

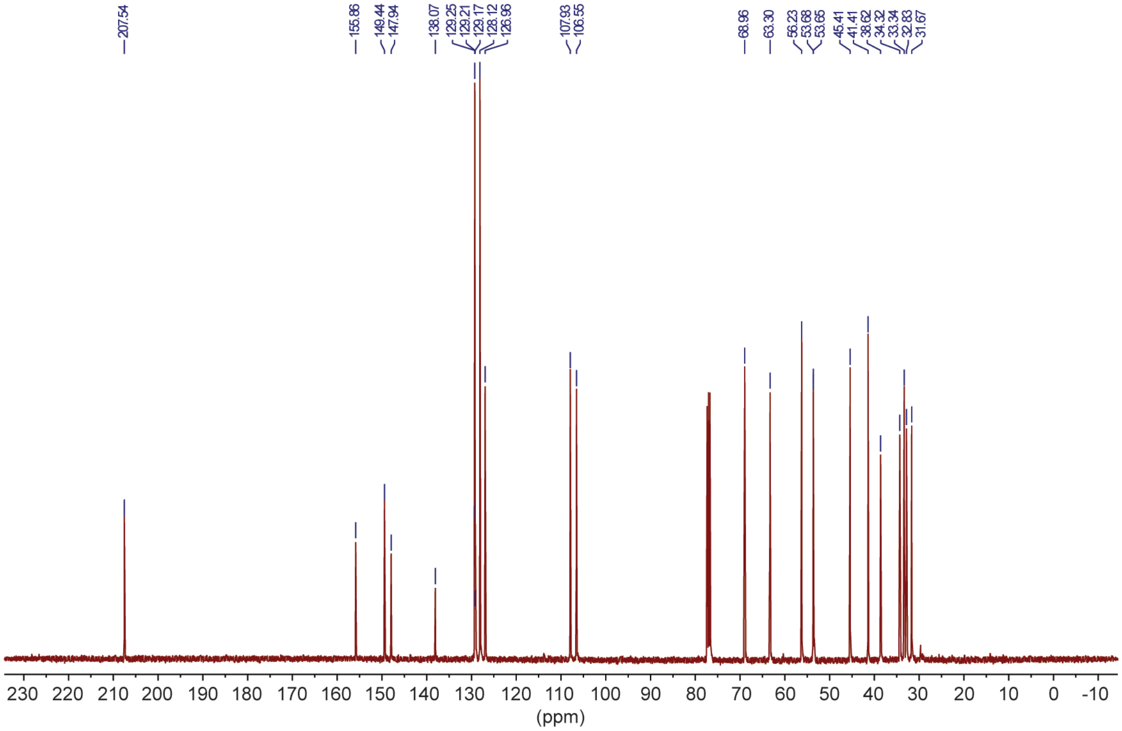

Fig. S26: ^{13}C NMR spectrum for compound **8d** in $CDCl_3$ (100 MHz).

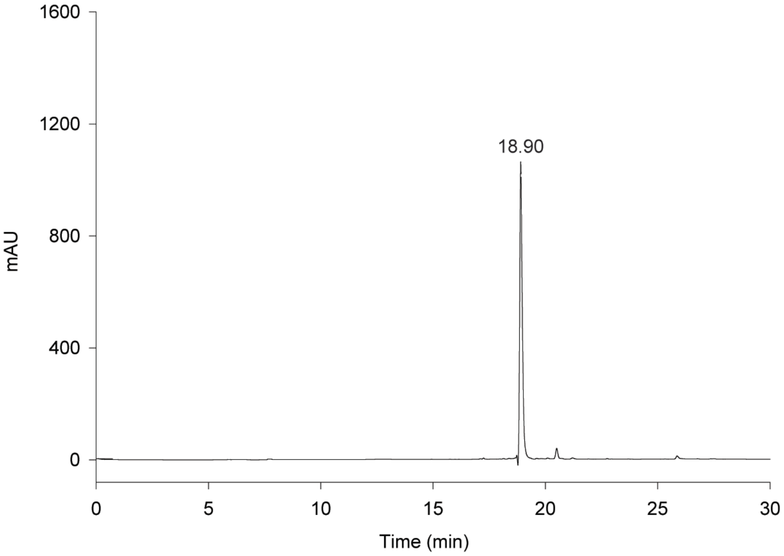

Fig. S27: HPLC trace for compound **8d**. R_t = 18.90 min.

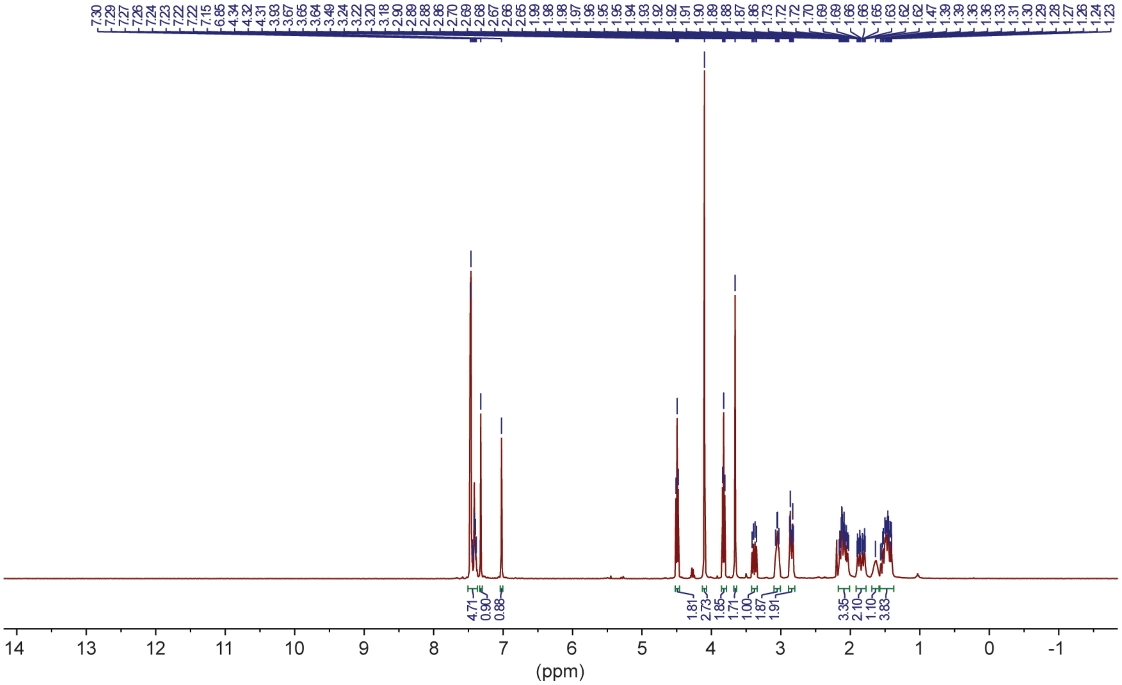

Fig. S28: ^{1}H NMR spectrum for compound **8e** in $CDCl_3$ (400 MHz).

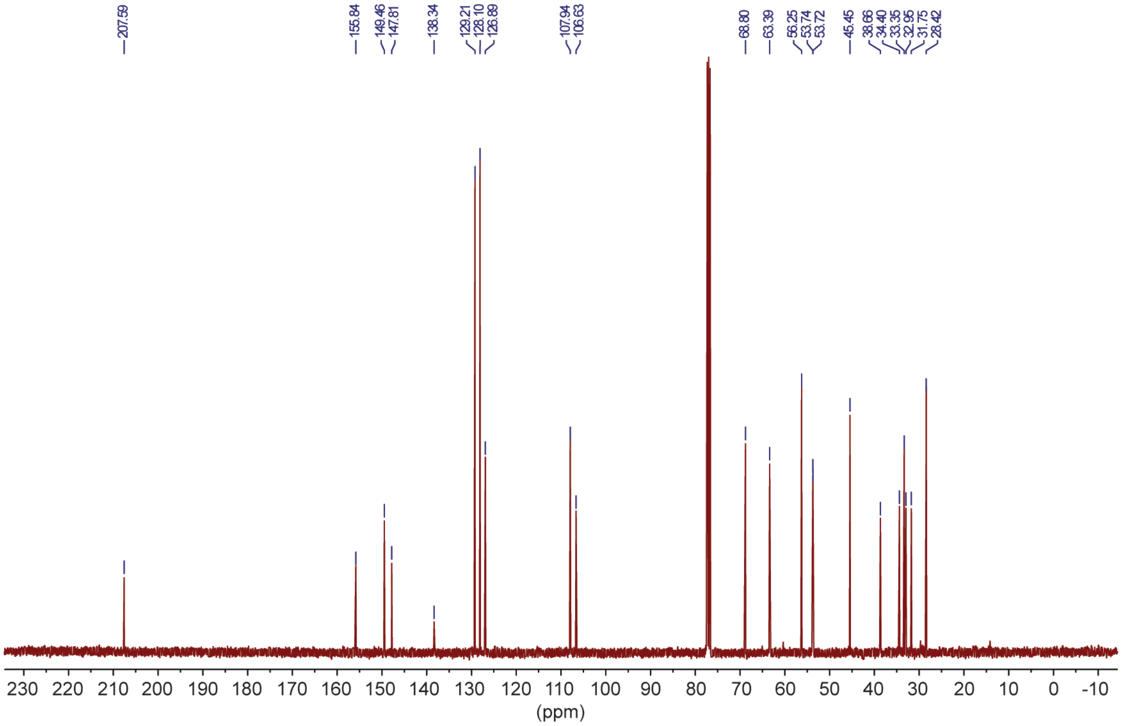

Fig. S29: ^{13}C NMR spectrum for compound **8e** in $CDCl_3$ (100 MHz).

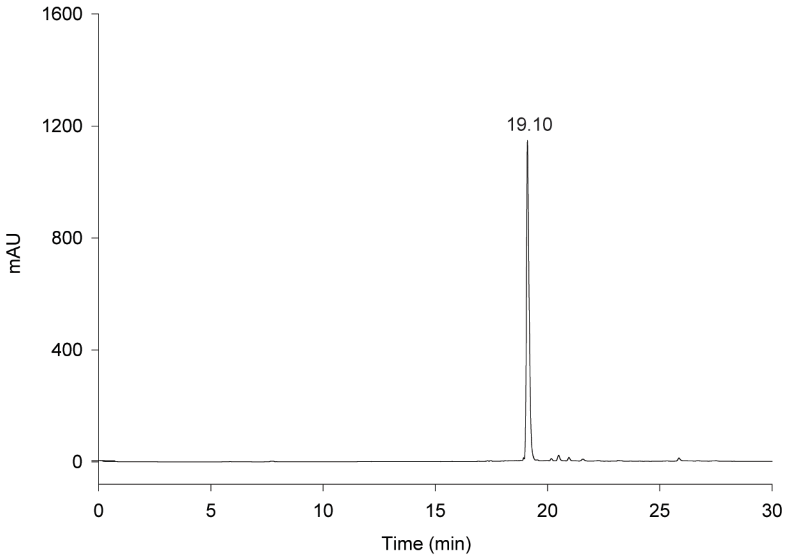

Fig. S30: HPLC trace for compound **8e**. R_t = 19.10 min.

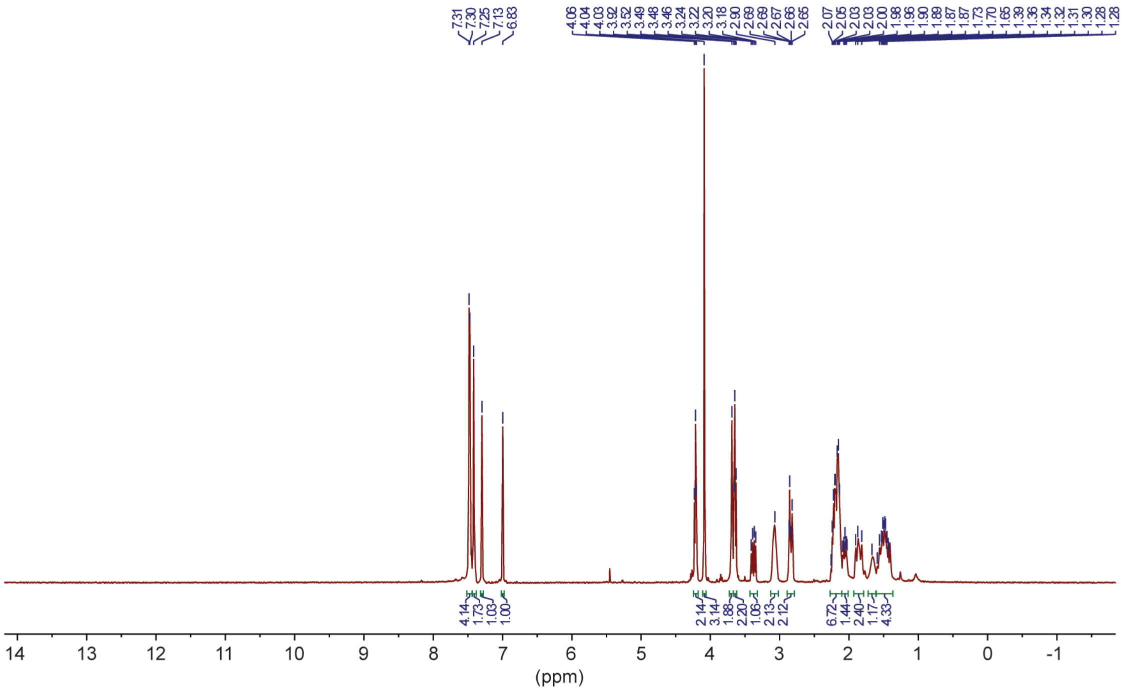

Fig. S31: ^{1}H NMR spectrum for compound **8f** in $CDCl_3$ (400 MHz).

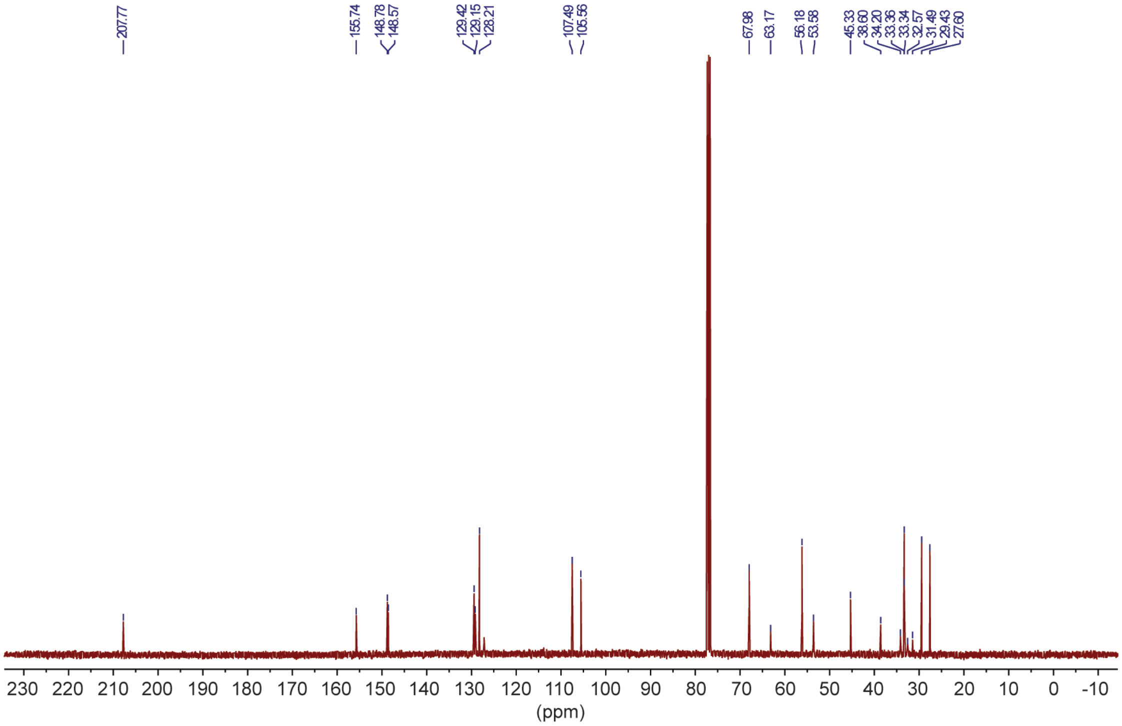

Fig. S32: ^{13}C NMR spectrum for compound **8f** in $CDCl_3$ (100 MHz).

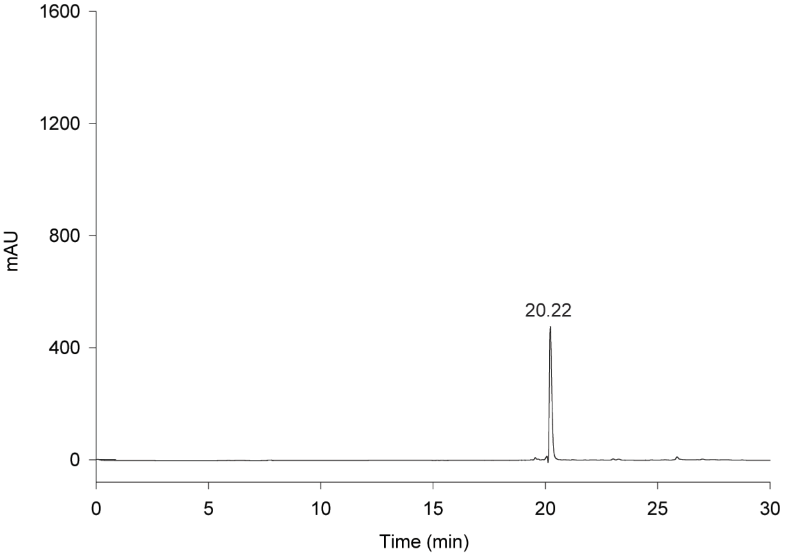

Fig. S33: HPLC trace for compound **8f**. R_t = 20.22 min.

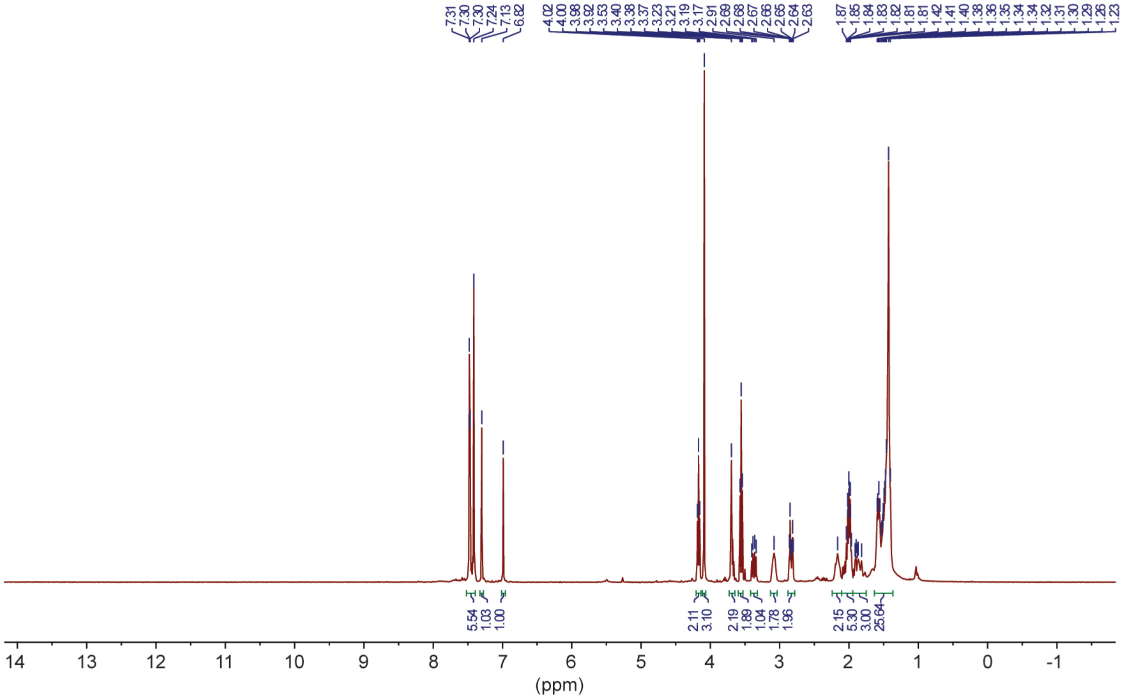

Fig. S34: ^{1}H NMR spectrum for compound **8g** in $CDCl_3$ (400 MHz).

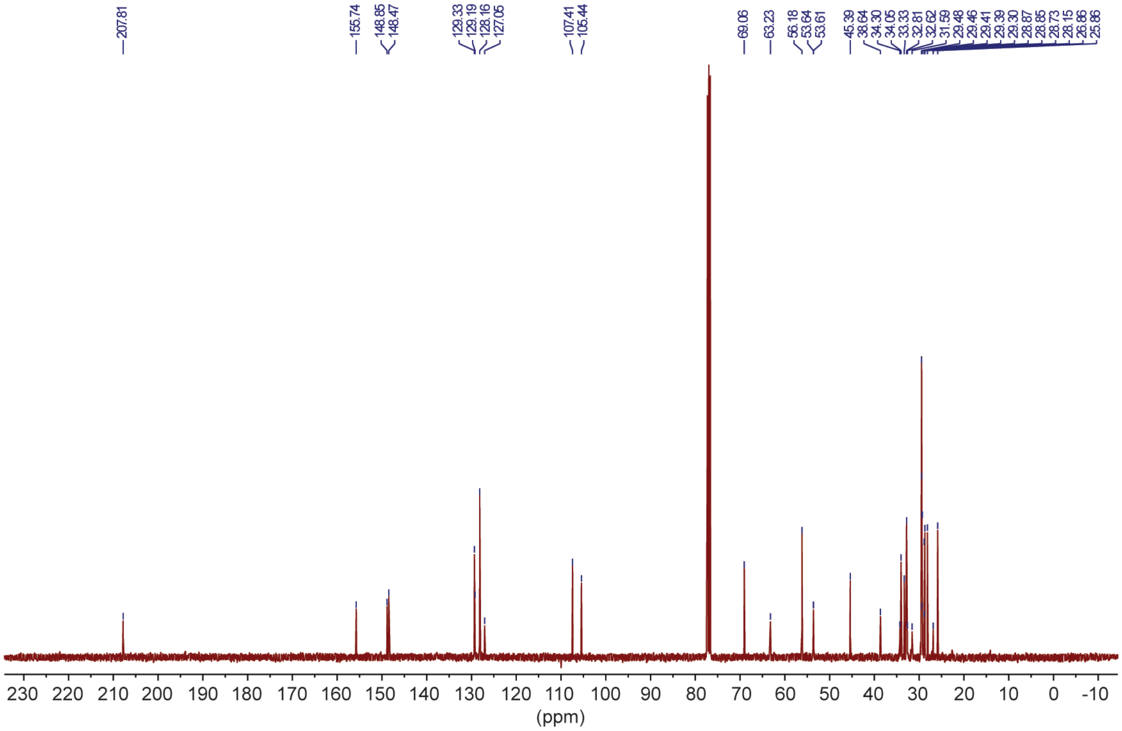

Fig. S35: ^{13}C NMR spectrum for compound **8g** in $CDCl_3$ (100 MHz).

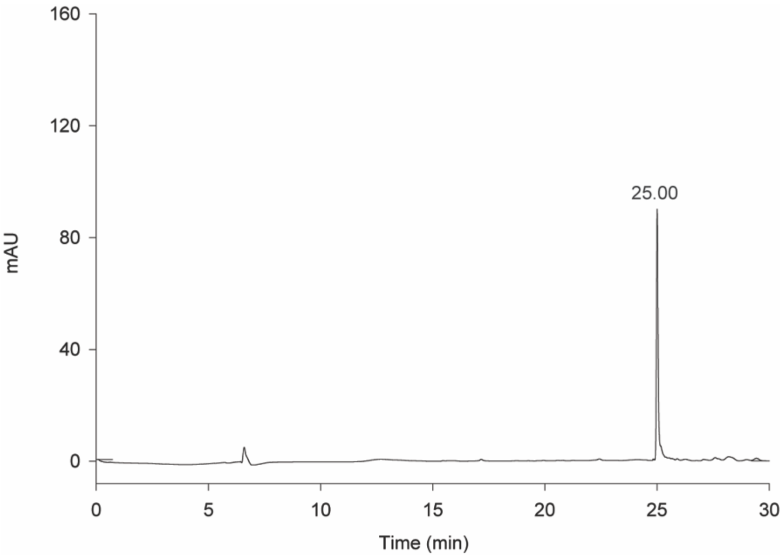

Fig. S36: HPLC trace for compound **8g**. R_t = 25.00 min.

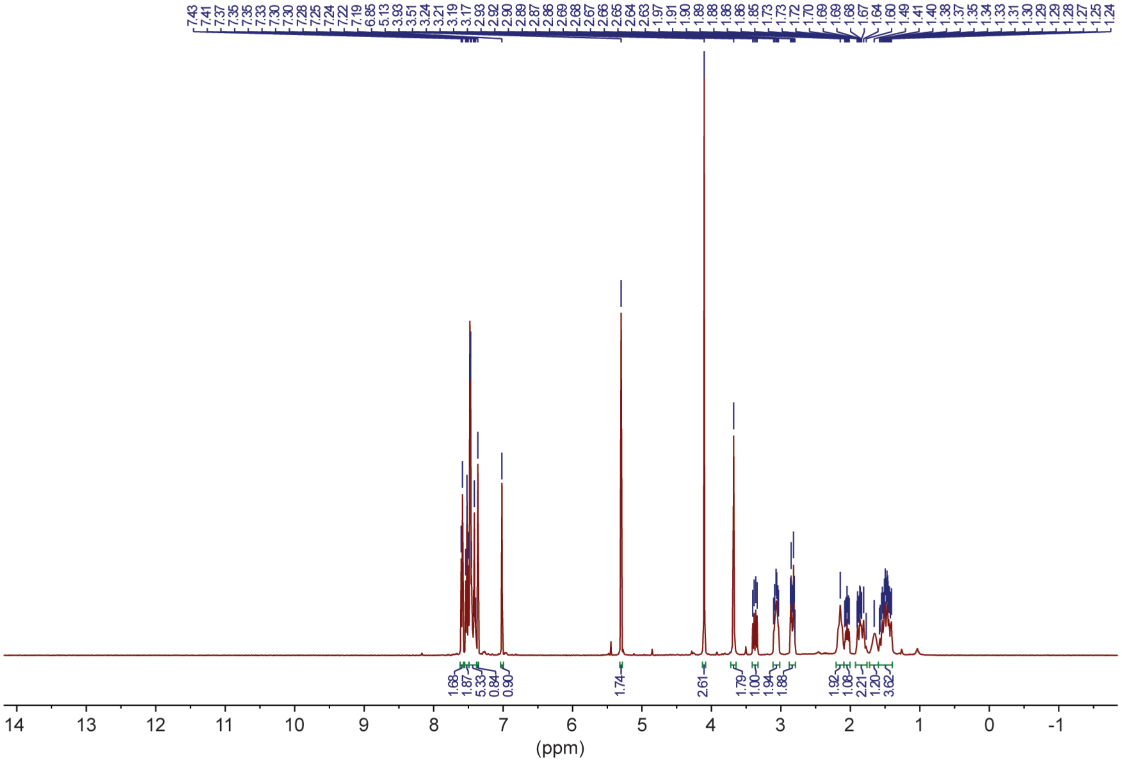

Fig. S37: ^1H NMR spectrum for compound **8h** in $CDCl_3$ (400 MHz).

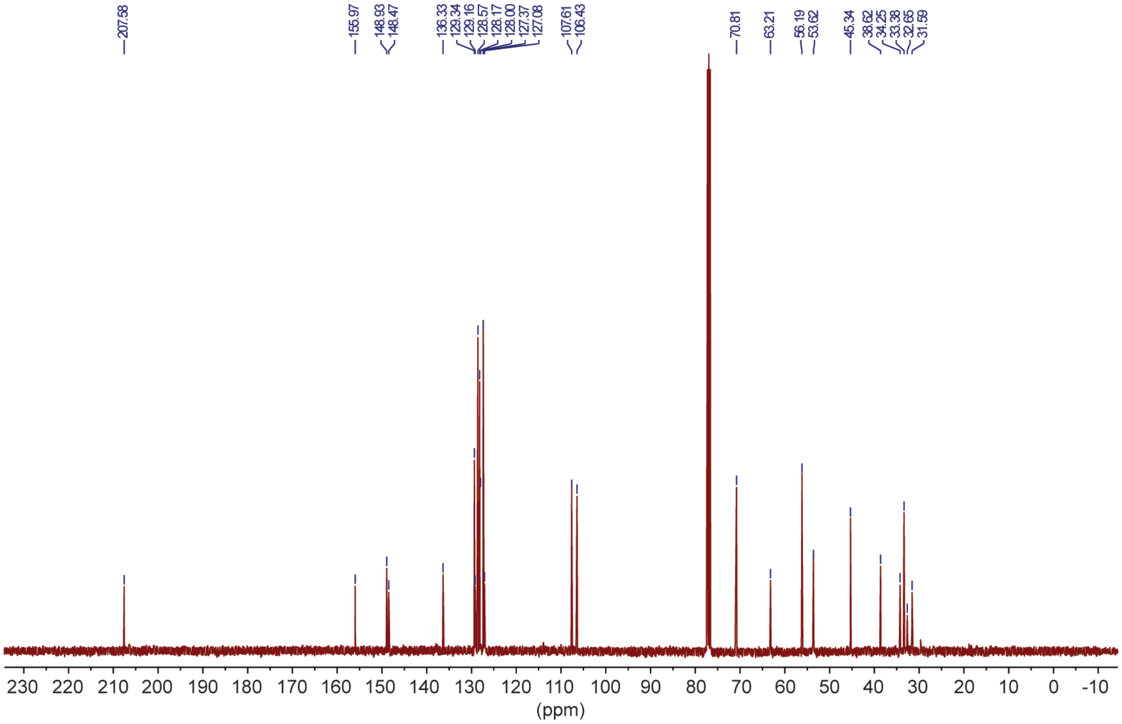

Fig. S38: ^{13}C NMR spectrum for compound **8h** in $CDCl_3$ (100 MHz).

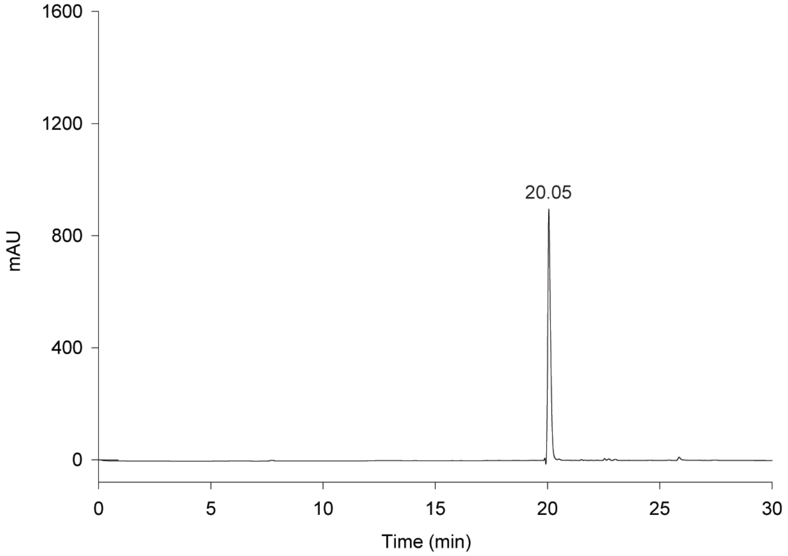

Fig. S39: HPLC trace for compound **8h**. R_t = 20.05 min.

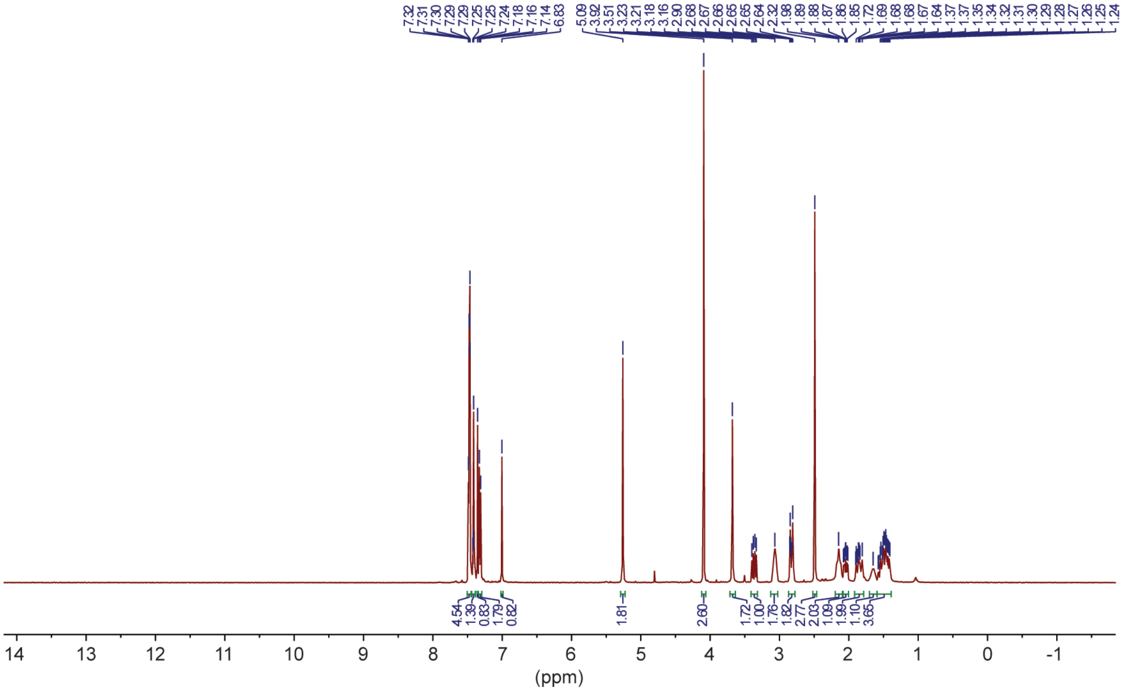

Fig. S40: ^{1}H NMR spectrum for compound **8i** in $CDCl_3$ (400 MHz).

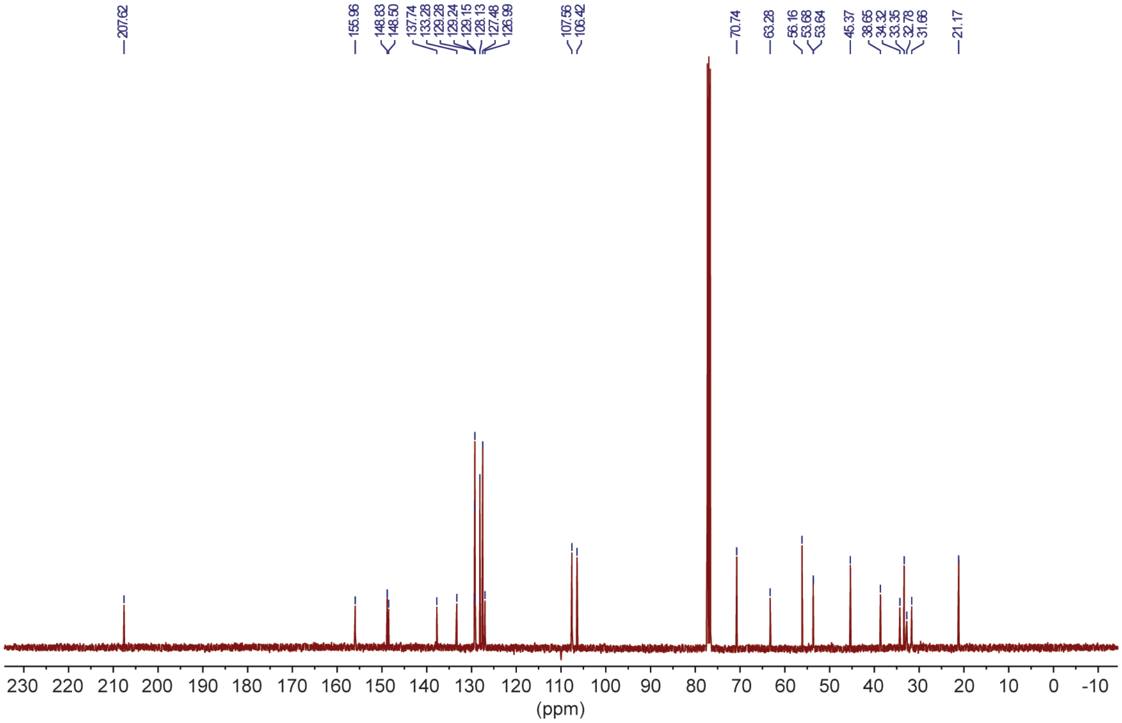

Fig. S41: ^{13}C NMR spectrum for compound **8i** in $CDCl_3$ (100 MHz).

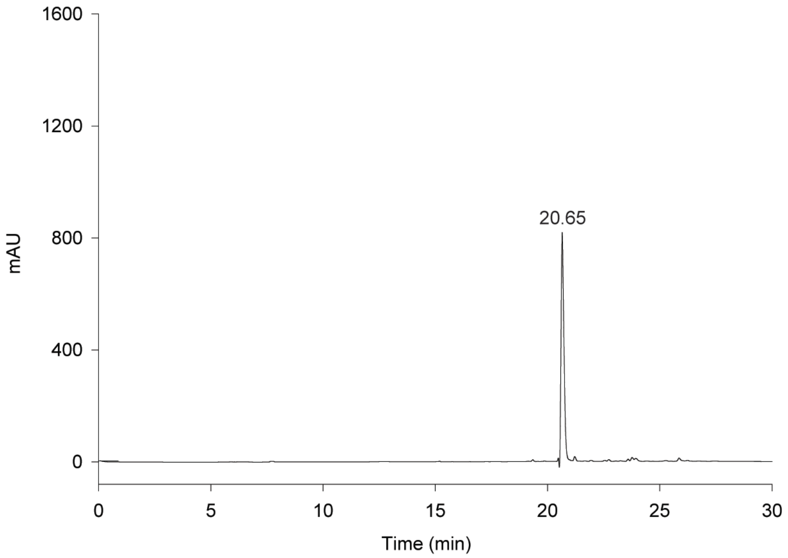

Fig. S42: HPLC trace for compound **8i**. R_t = 20.65 min.

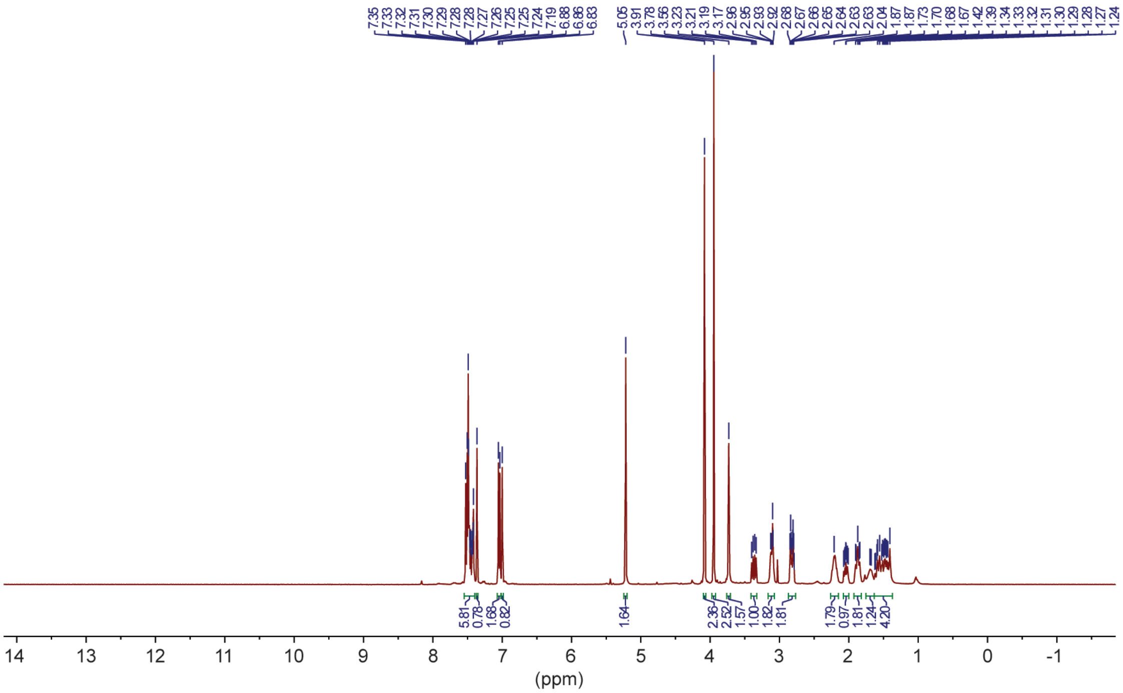

Fig. S43: ^{1}H NMR spectrum for compound **8j** in $CDCl_3$ (400 MHz).

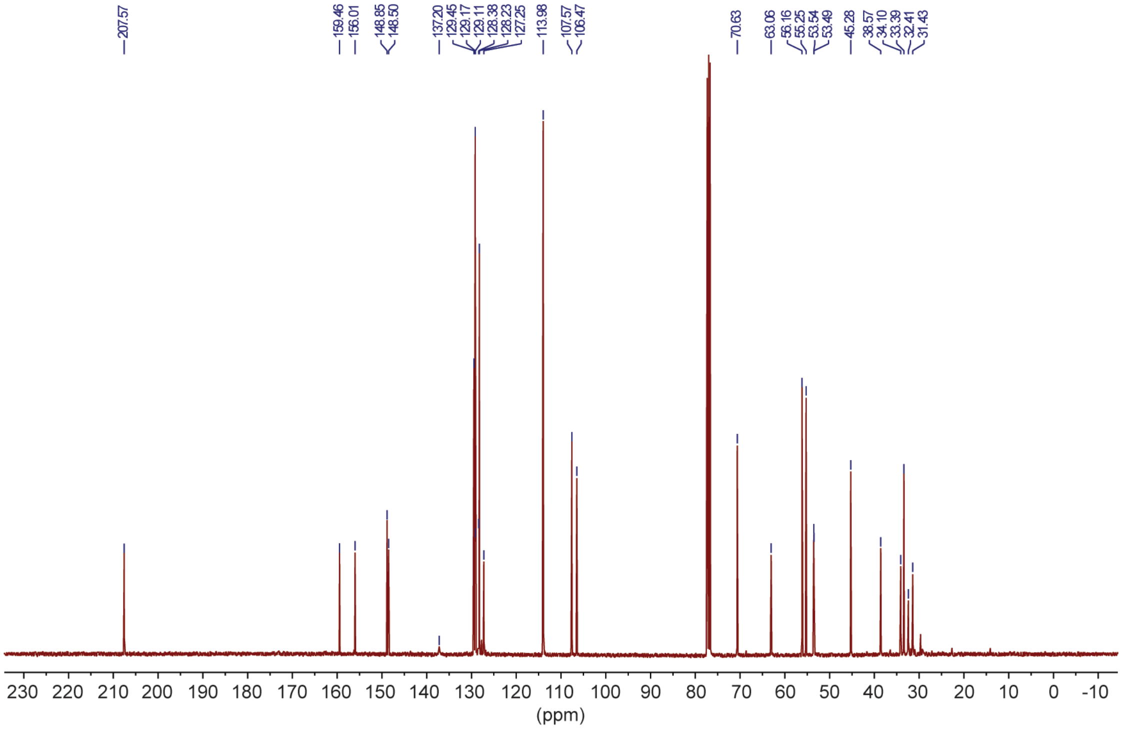

Fig. S44: ^{13}C NMR spectrum for compound **8j** in $CDCl_3$ (100 MHz).

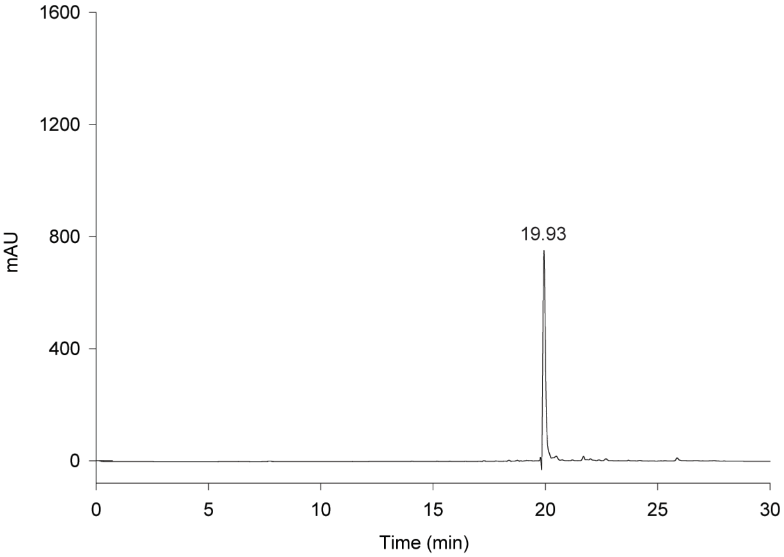

Fig. S45: HPLC trace for compound **8j**. R_t = 19.93 min.

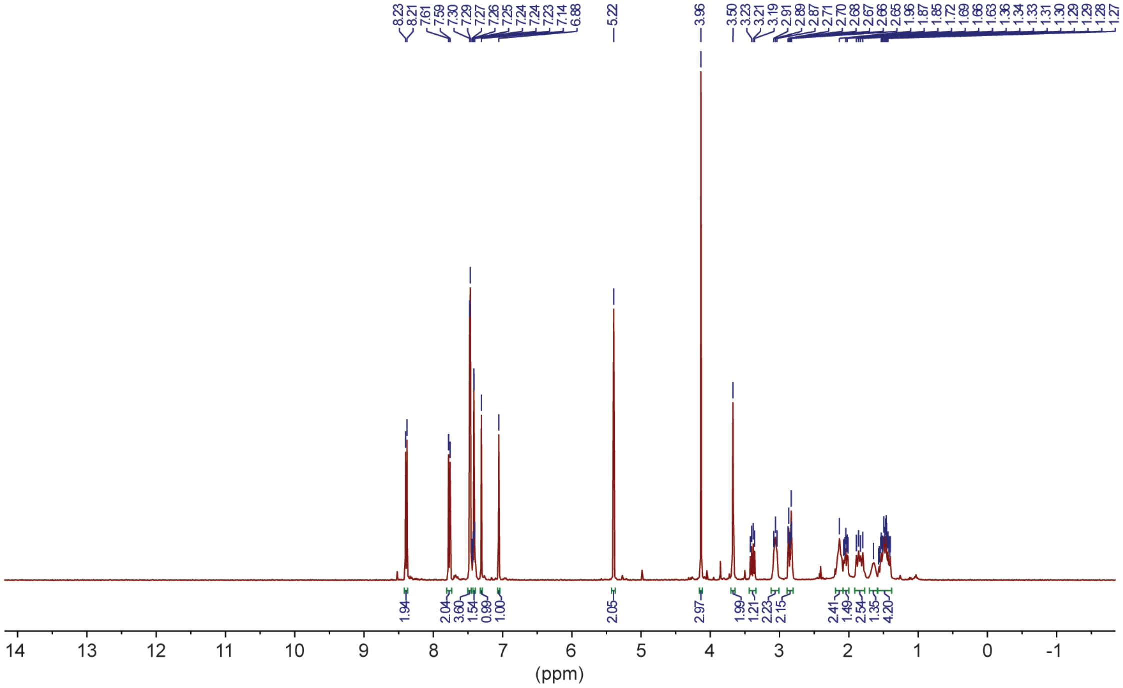

Fig. S46: ^{1}H NMR spectrum for compound **8k** in $CDCl_3$ (400 MHz).

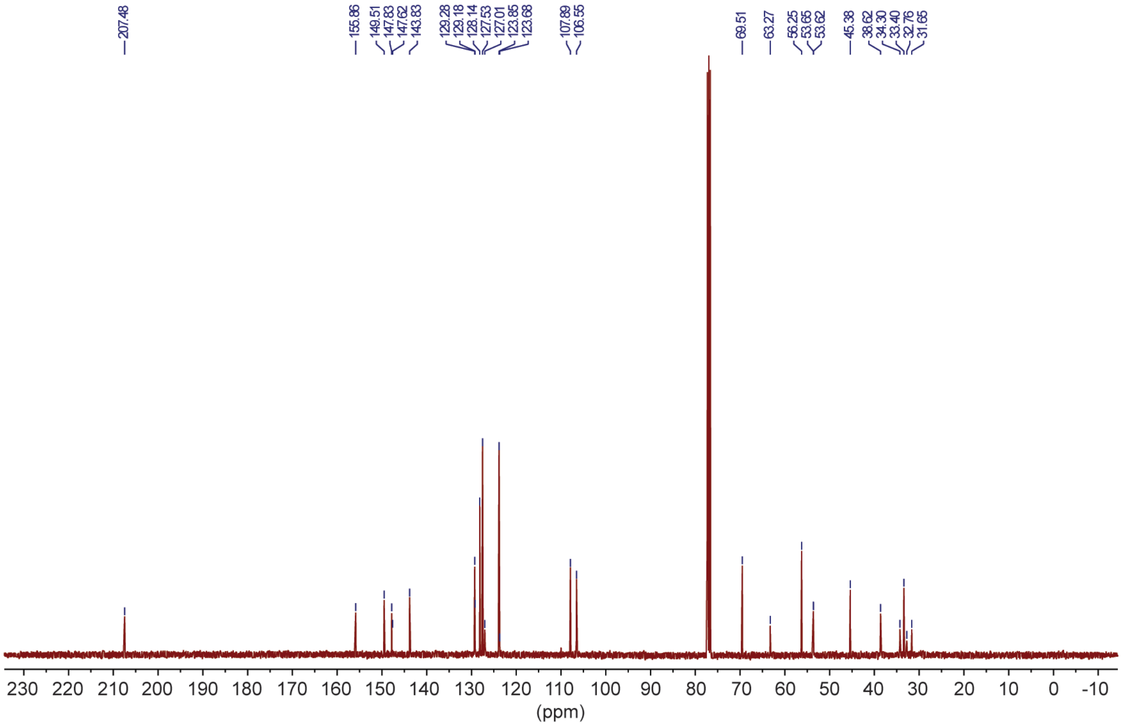

Fig. S47: ^{13}C NMR spectrum for compound **8k** in $CDCl_3$ (100 MHz).

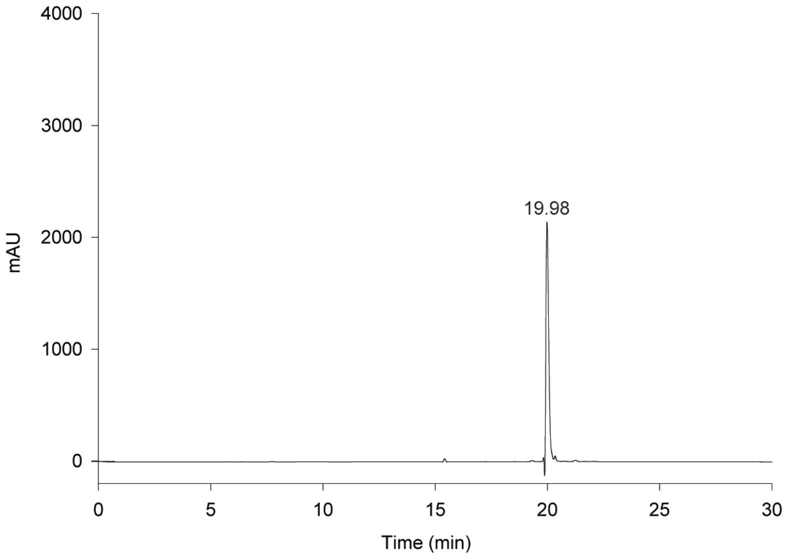

Fig. S48: HPLC trace for compound **8k**. R_t = 19.98 min.

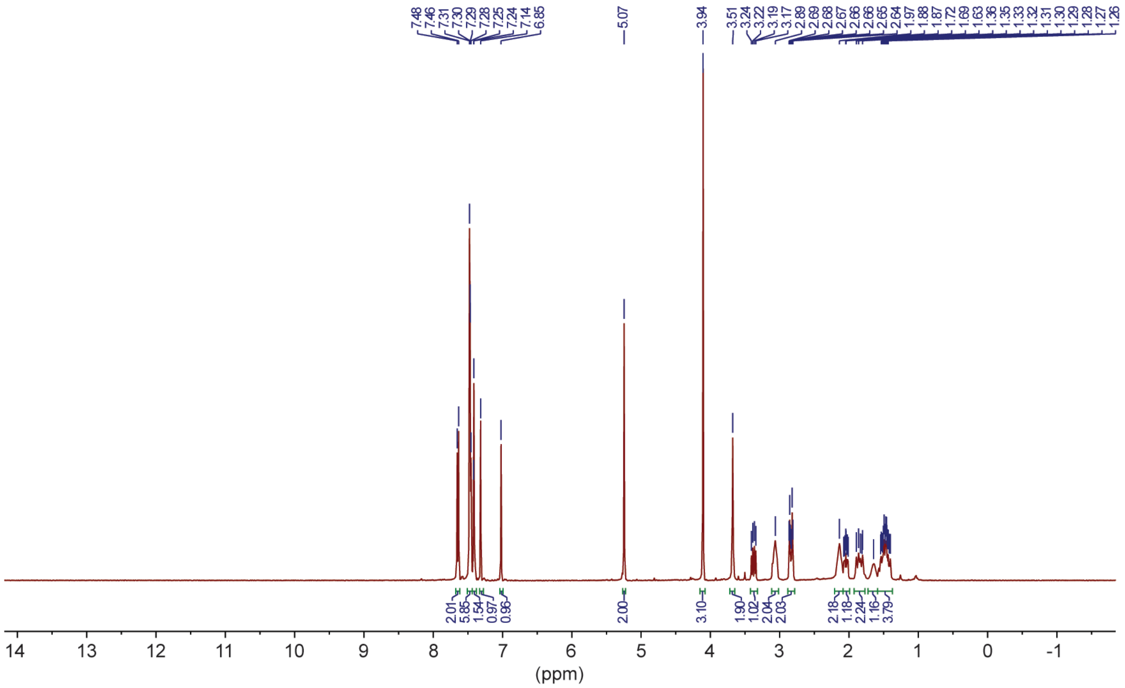

Fig. S49: ^{1}H NMR spectrum for compound **8l** in $CDCl_3$ (400 MHz).

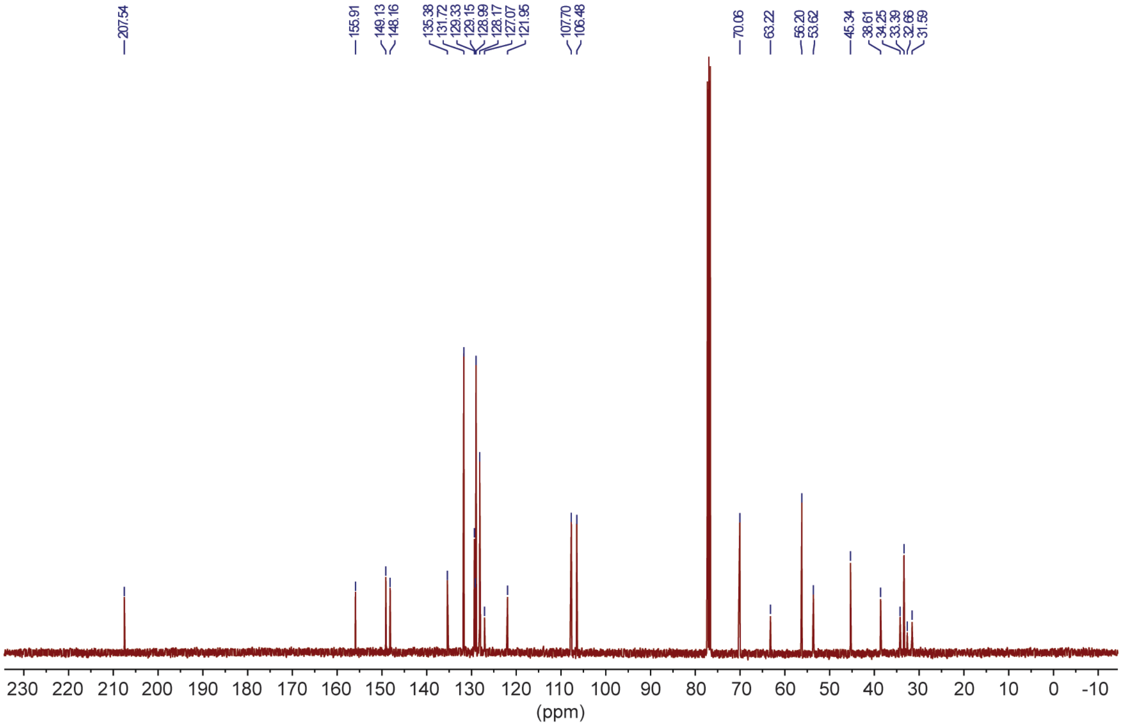

Fig. S50: ^{13}C NMR spectrum for compound **8l** in $CDCl_3$ (100 MHz).

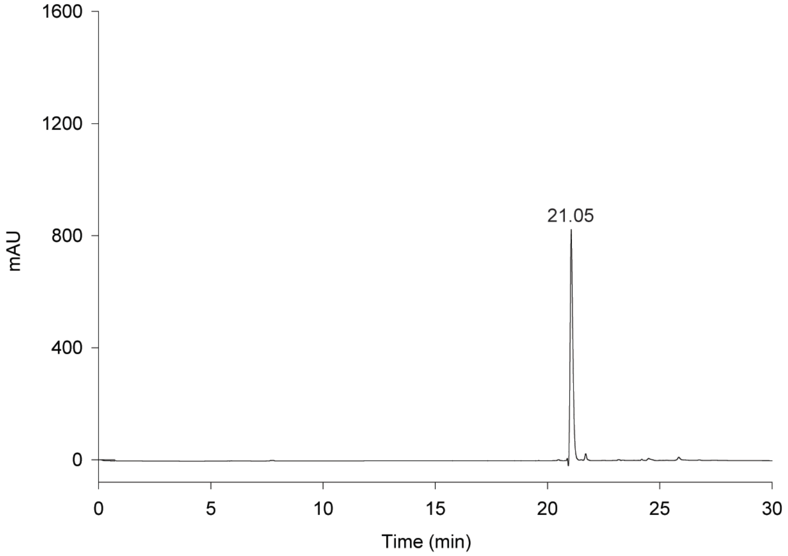

Fig. S51: HPLC trace for compound **8l**. R_t = 21.05 min.

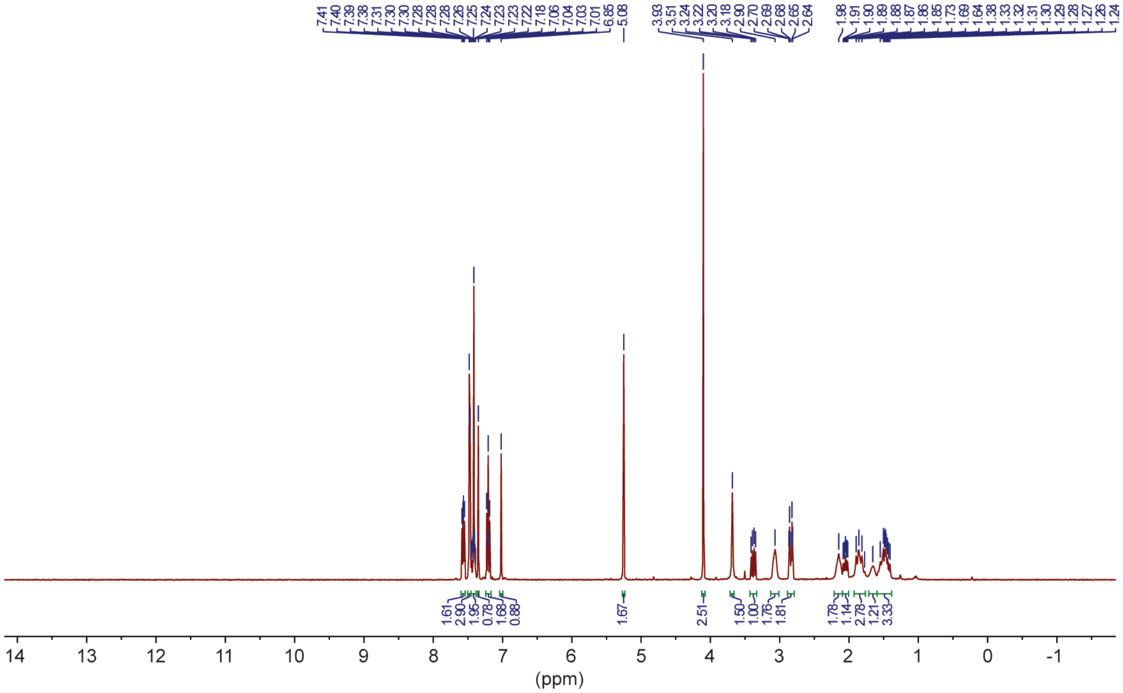

Fig. S52: ^{1}H NMR spectrum for compound **8m** in $CDCl_3$ (400 MHz).

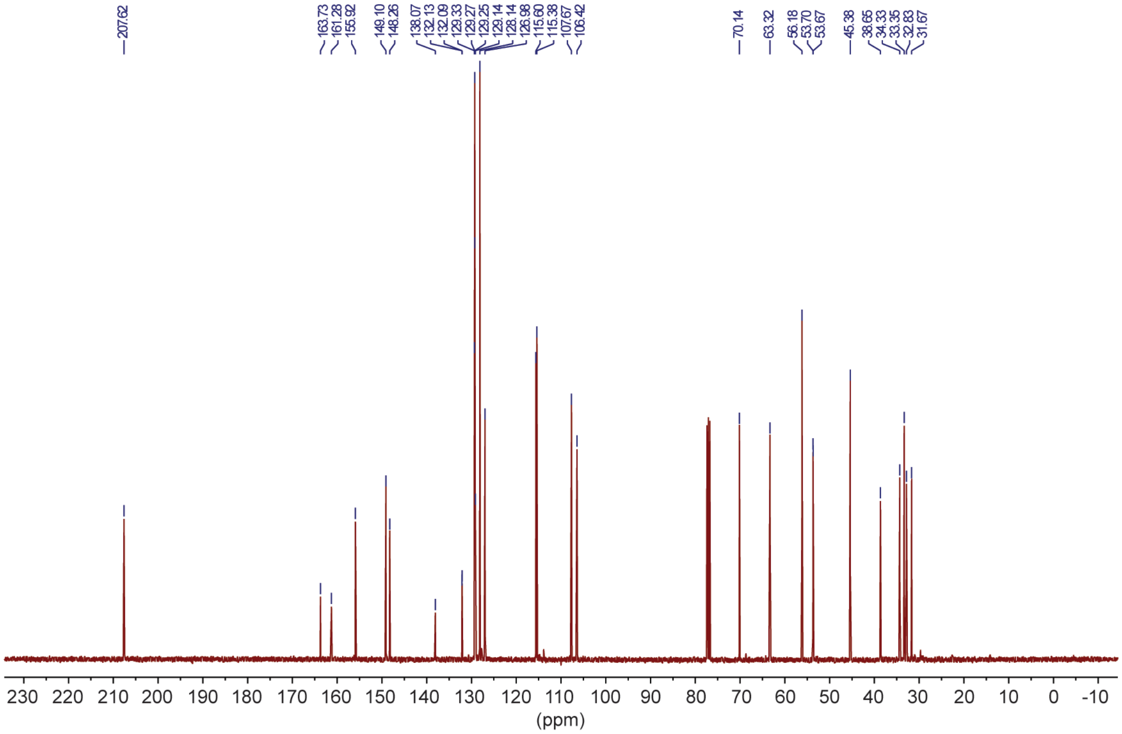

Fig. S53: ^{13}C NMR spectrum for compound **8m** in $CDCl_3$ (100 MHz).

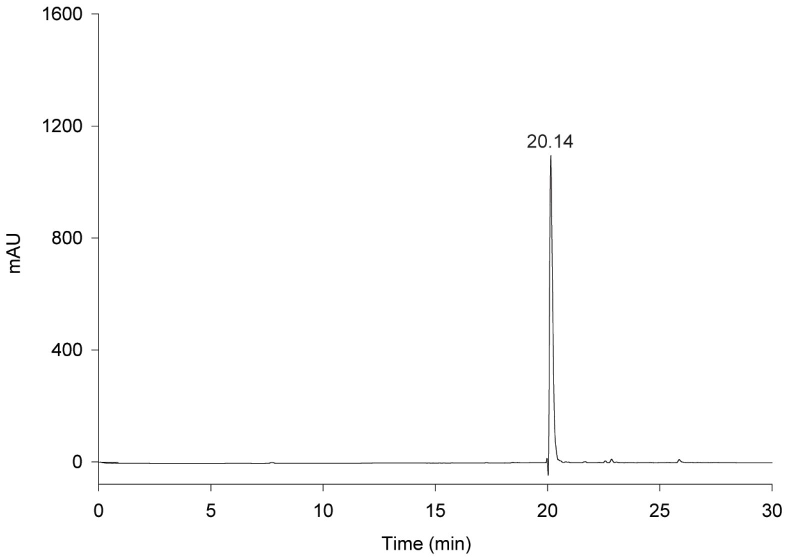

Fig. S54: HPLC trace for compound **8m**. R_t = 20.14 min.

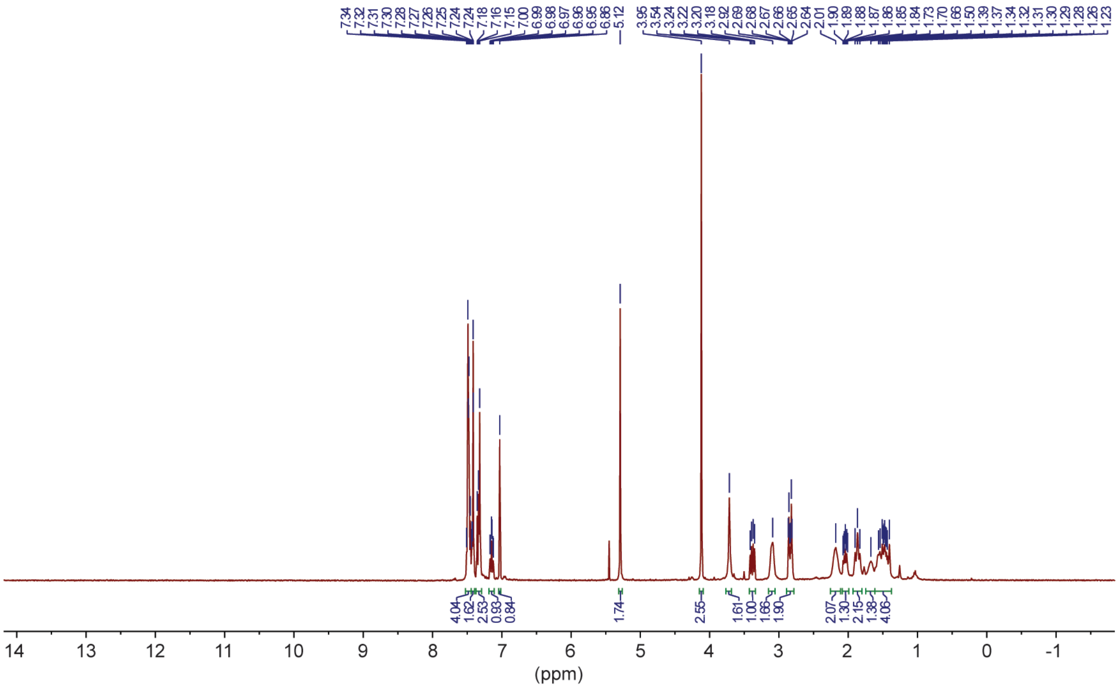

Fig. S55: ^{1}H NMR spectrum for compound **8n** in $CDCl_3$ (400 MHz).

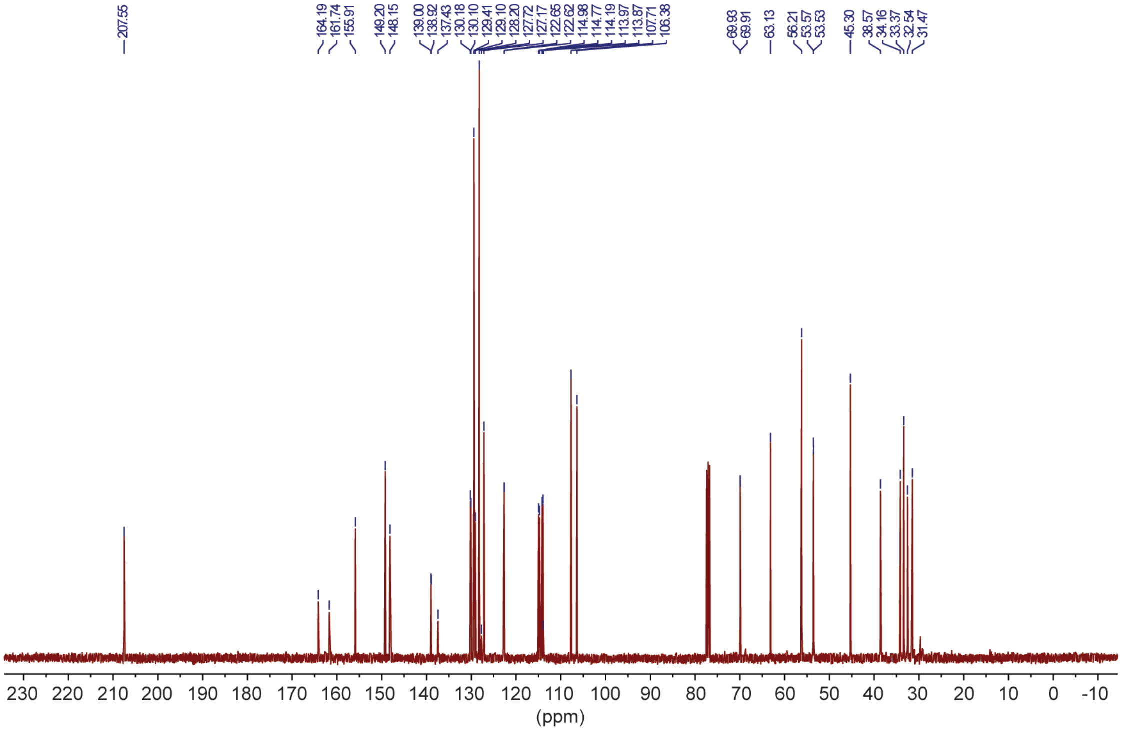

Fig. S56: ^{13}C NMR spectrum for compound **8n** in $CDCl_3$ (100 MHz).

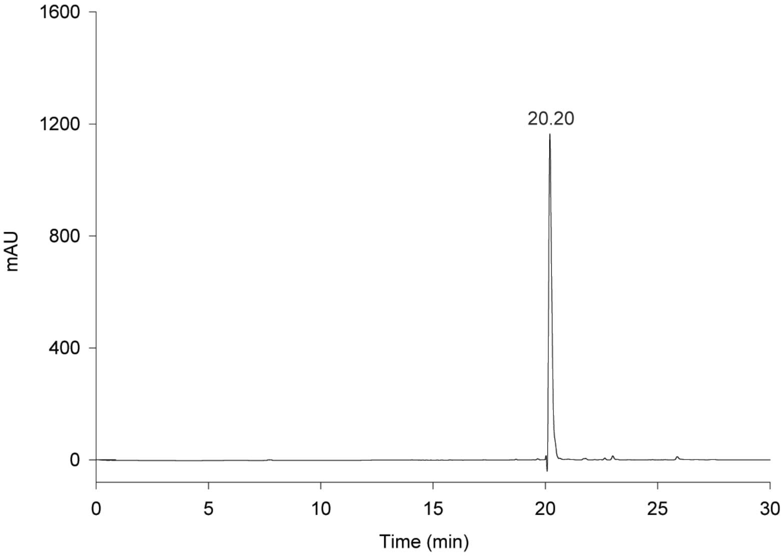

Fig. S57: HPLC trace for compound **8n**. R_t = 20.20 min.

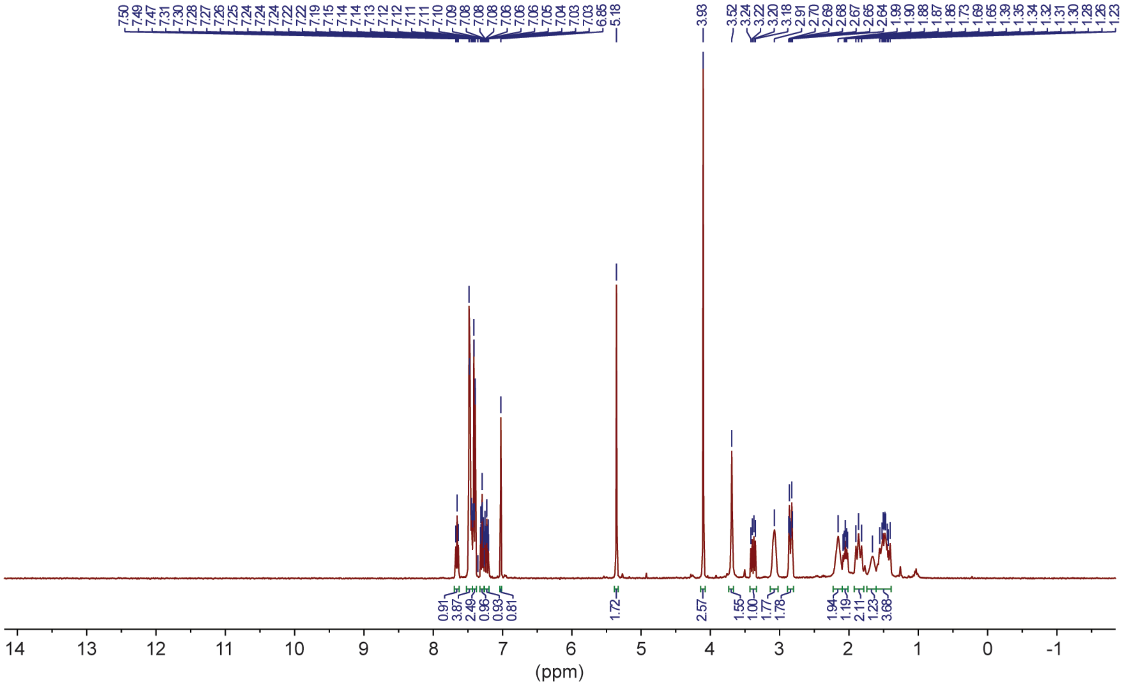

Fig. S58: ^{1}H NMR spectrum for compound **8o** in $CDCl_3$ (400 MHz).

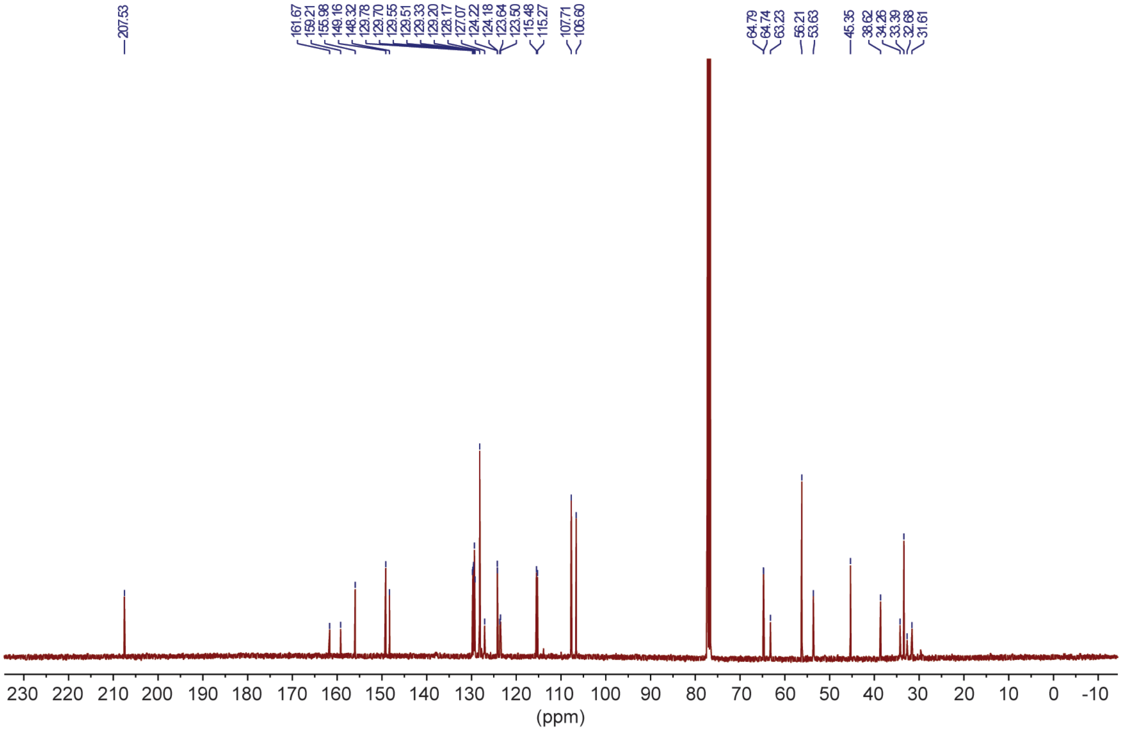

Fig. S59: ^{13}C NMR spectrum for compound **8o** in $CDCl_3$ (100 MHz).

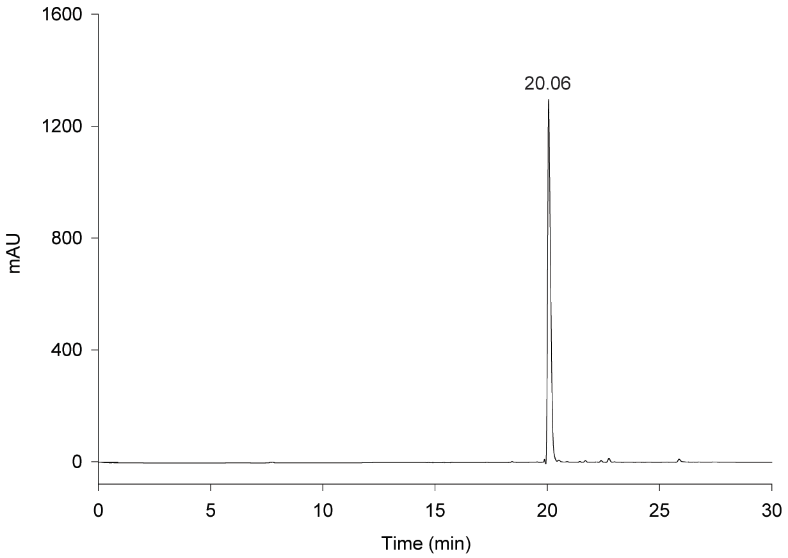

Fig. S60: HPLC trace for compound **8o**. R_t = 20.06 min.

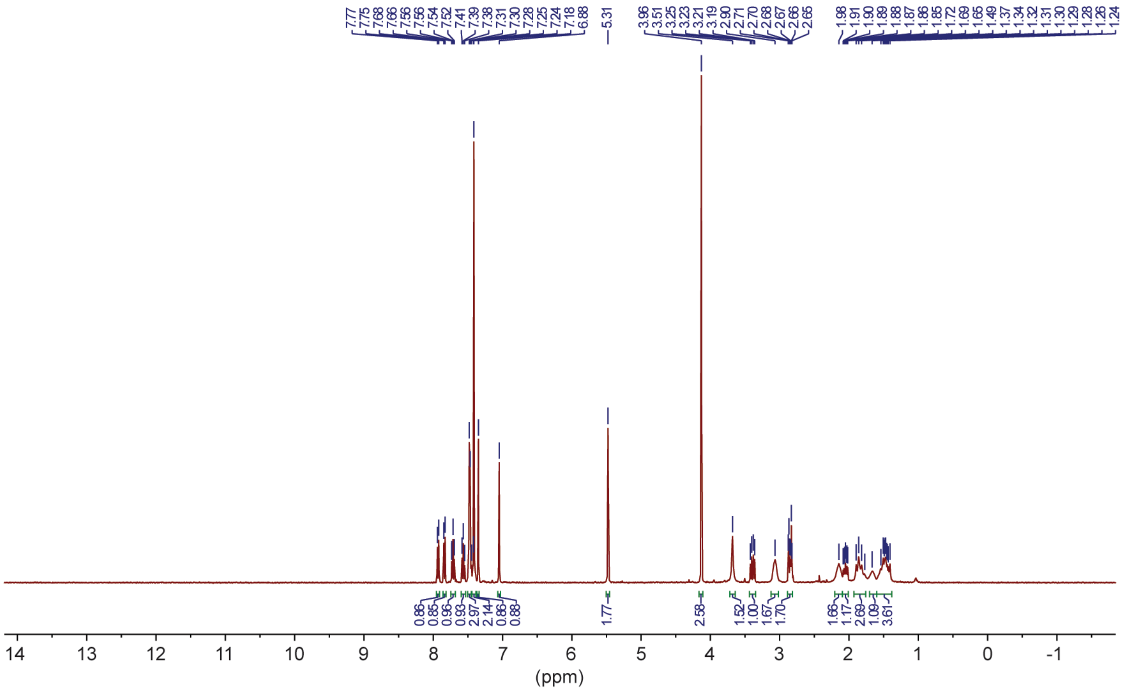

Fig. S61: ^{1}H NMR spectrum for compound **8p** in $CDCl_3$ (400 MHz).

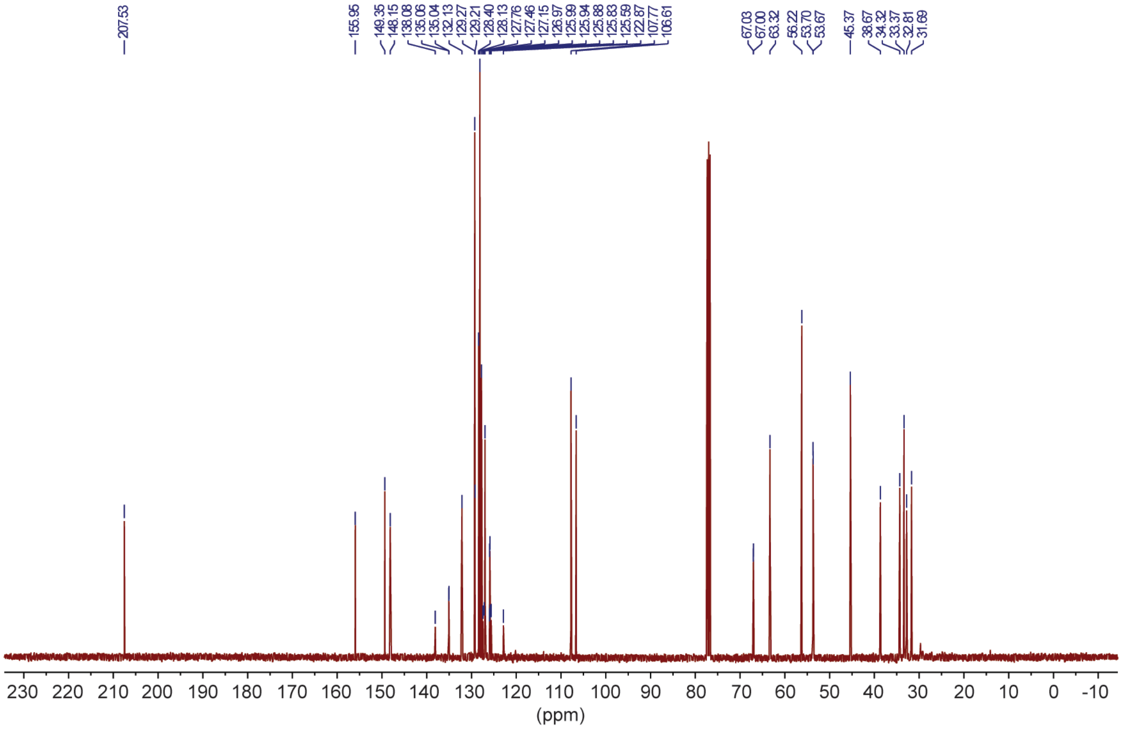

Fig. S62: ^{13}C NMR spectrum for compound **8p** in $CDCl_3$ (100 MHz).

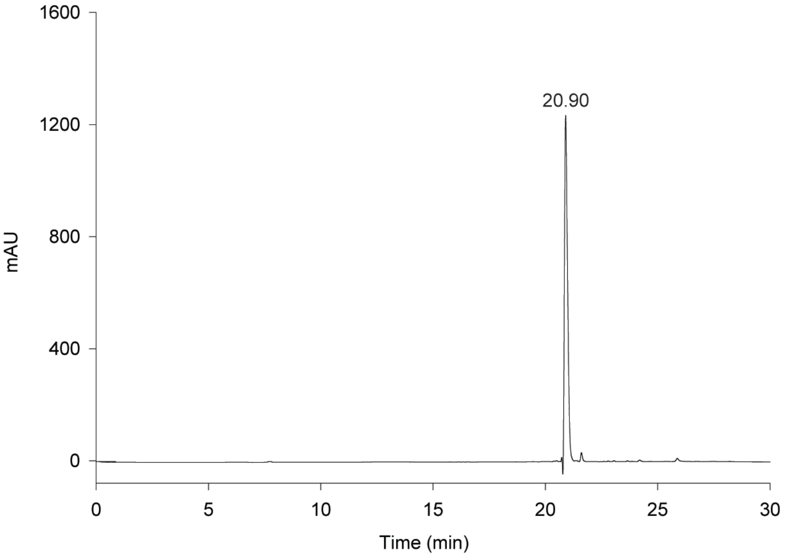

Fig. S63: HPLC trace for compound **8p**. R_t = 20.90 min.

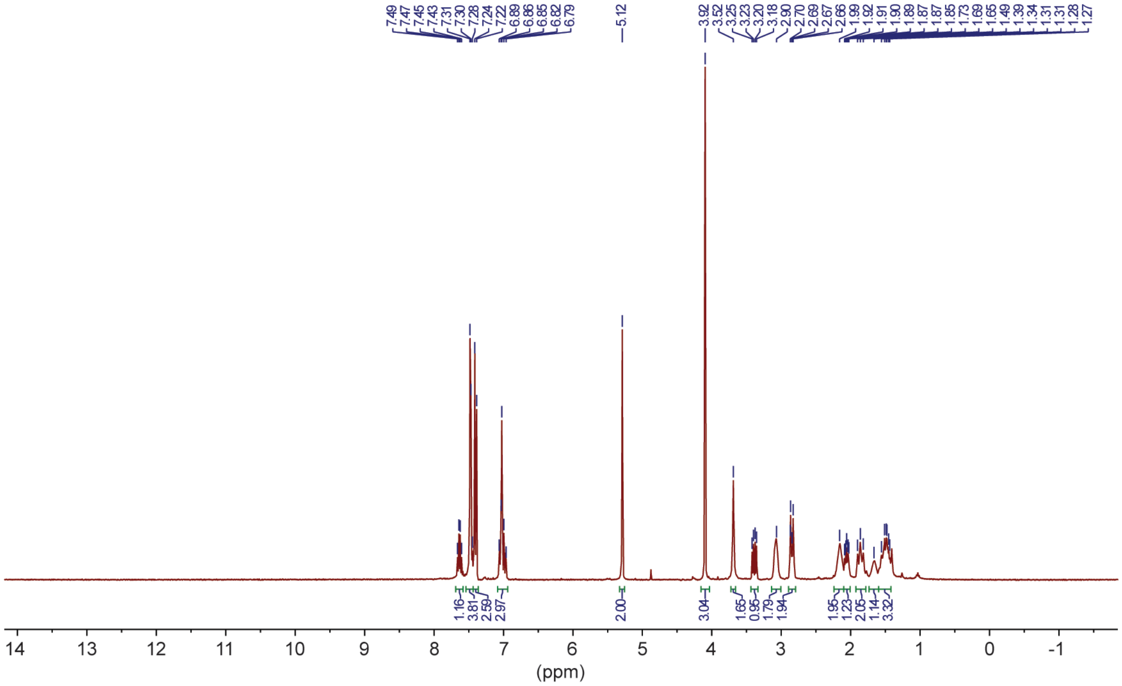

Fig. S64: ^{1}H NMR spectrum for compound **8q** in $CDCl_3$ (400 MHz).

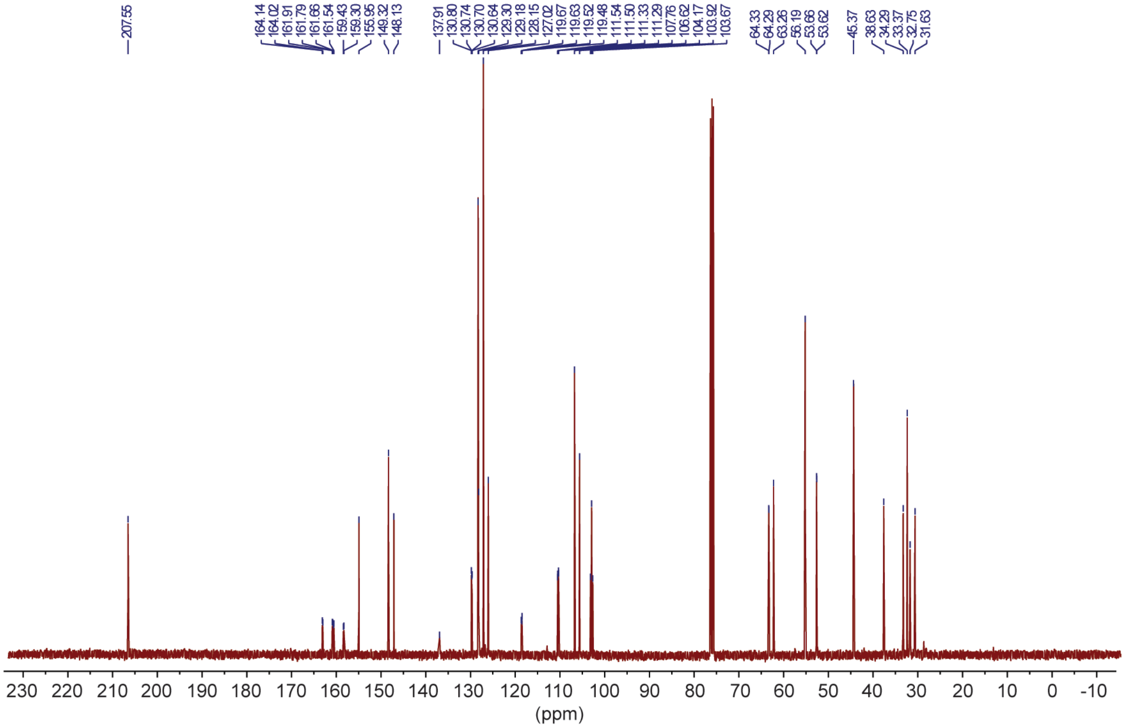

Fig. S65: ^{13}C NMR spectrum for compound **8q** in $CDCl_3$ (100 MHz).

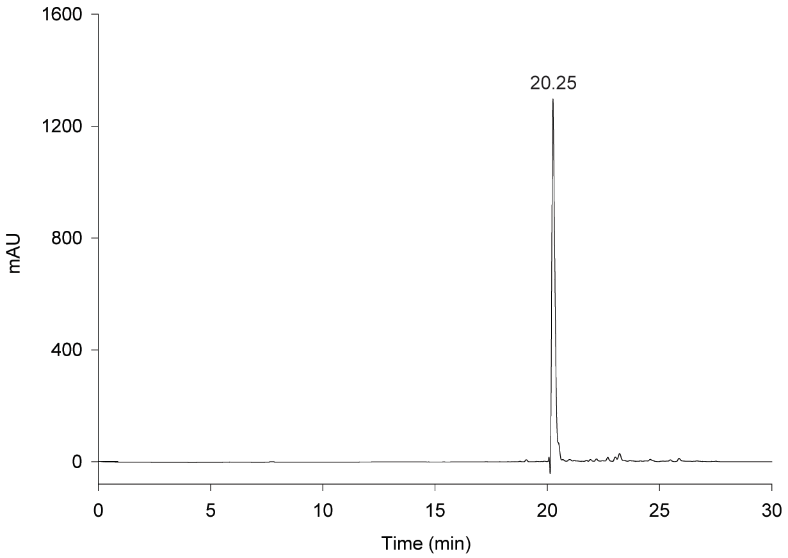

Fig. S66: HPLC trace for compound **8q**. R_t = 20.25 min.

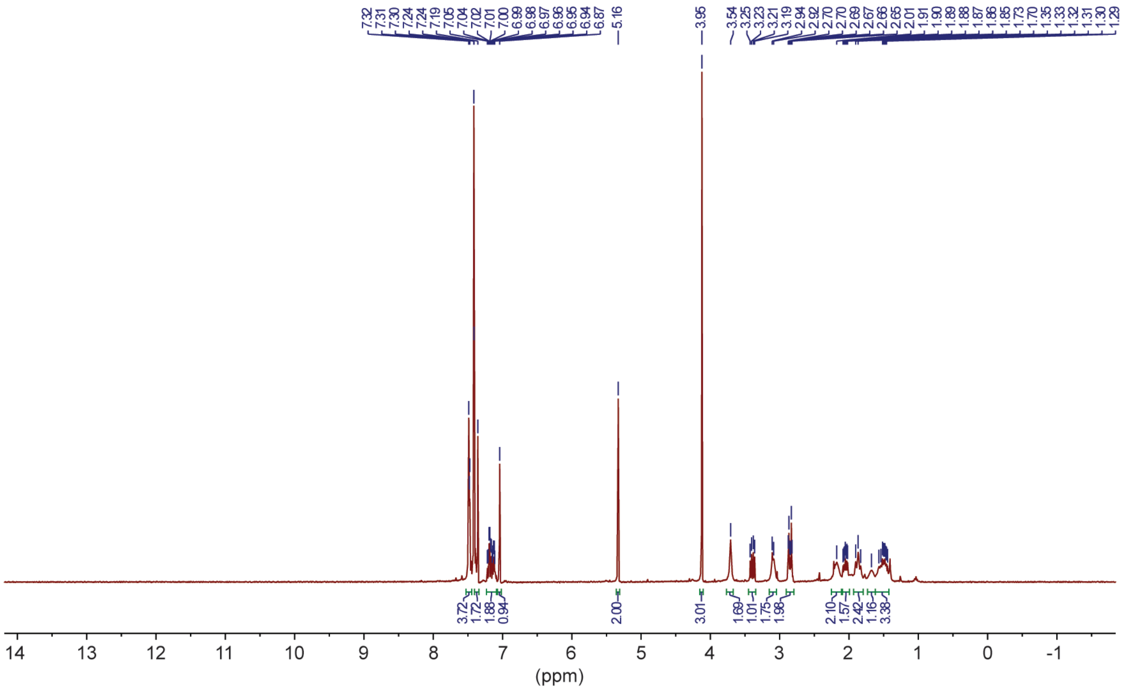

Fig. S67: ^1H NMR spectrum for compound **8r** in $CDCl_3$ (400 MHz).

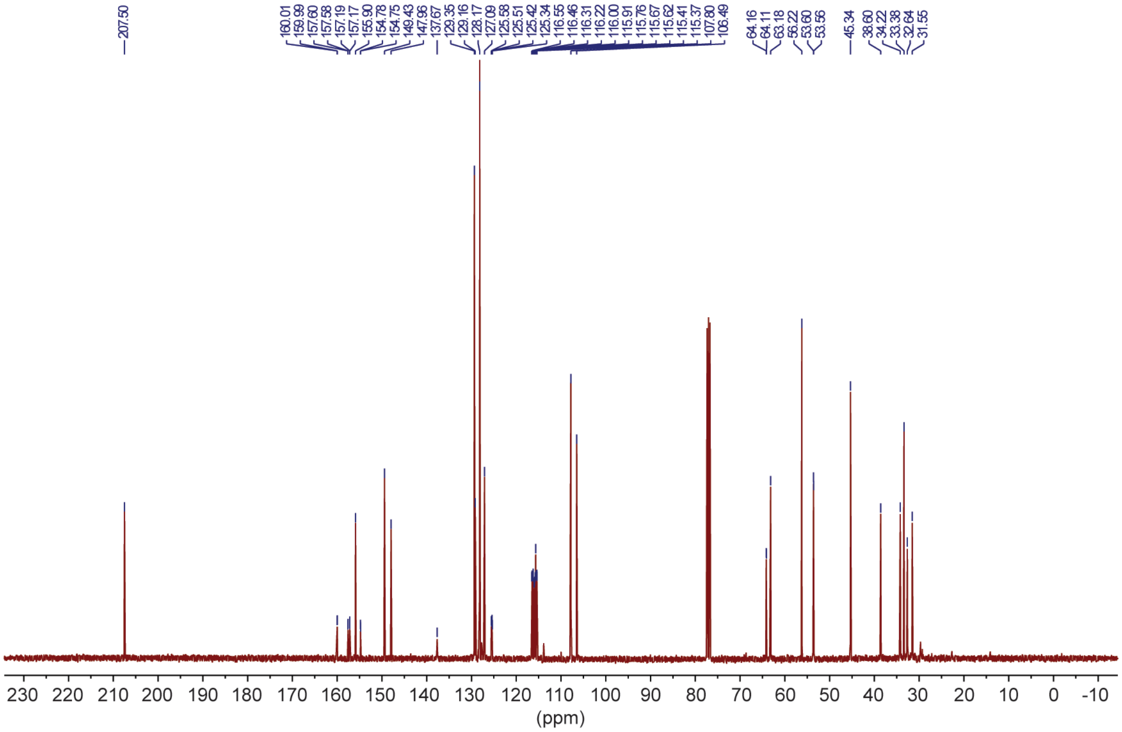

Fig. S68: ^{13}C NMR spectrum for compound **8r** in $CDCl_3$ (100 MHz).

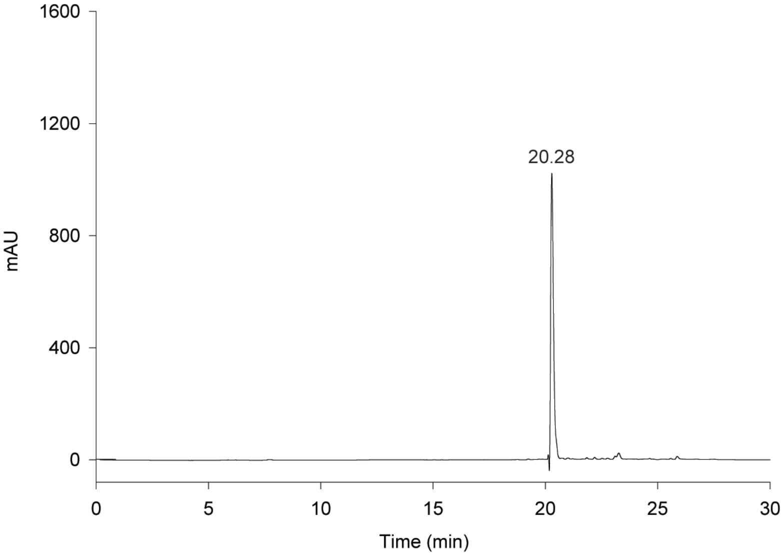

Fig. S69: HPLC trace for compound **8r**. R_t = 20.28 min.

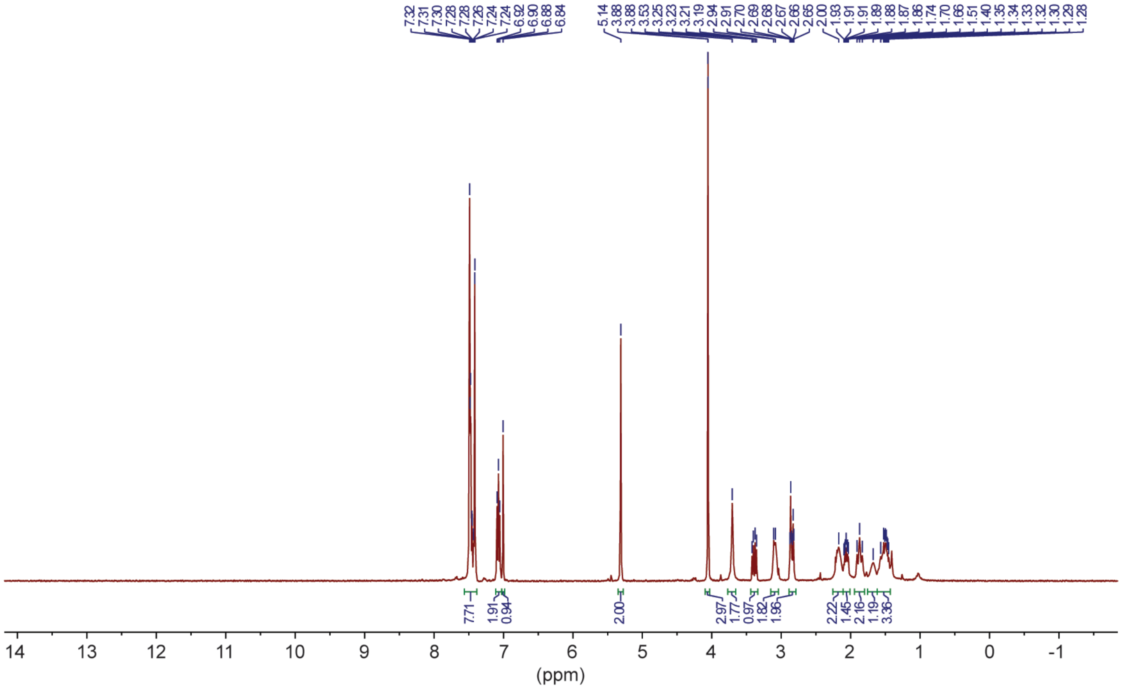

Fig. S70: ^{1}H NMR spectrum for compound **8s** in $CDCl_3$ (400 MHz).

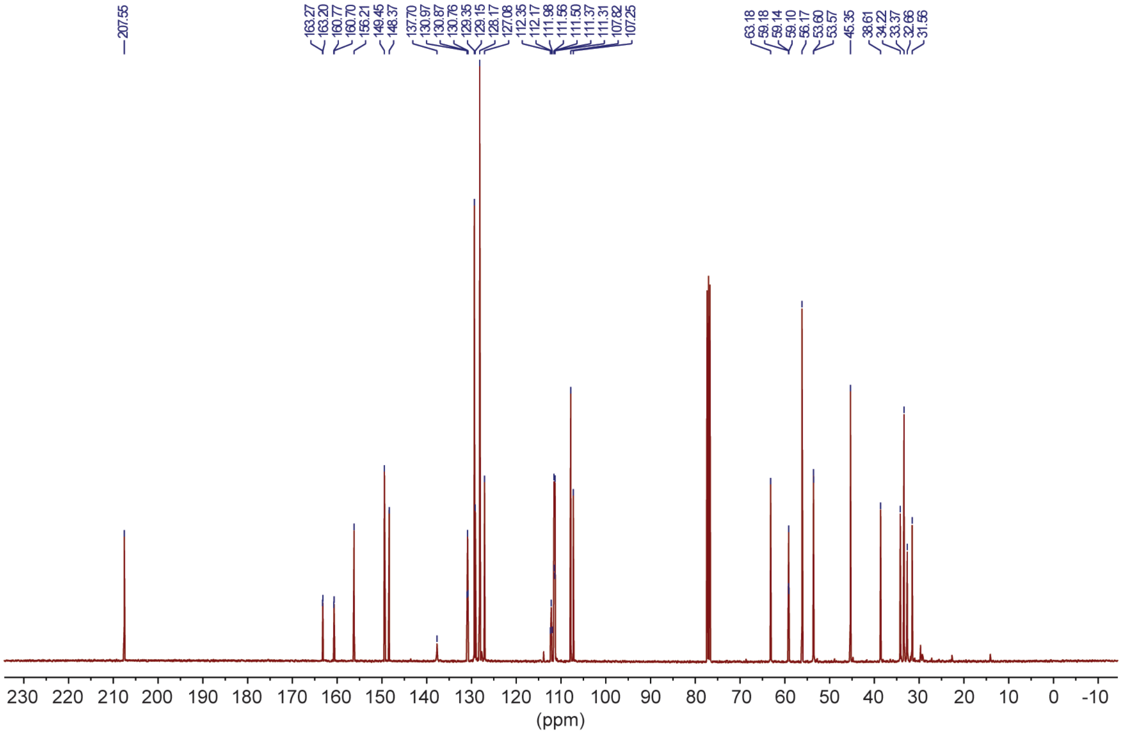

Fig. S71: ^{13}C NMR spectrum for compound **8s** in $CDCl_3$ (100 MHz).

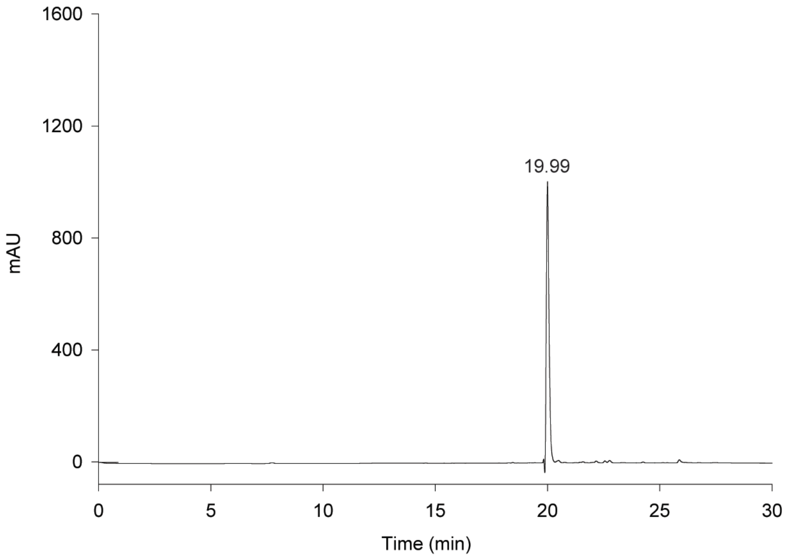

Fig. S72: HPLC trace for compound **8s**. R_t = 19.99 min.

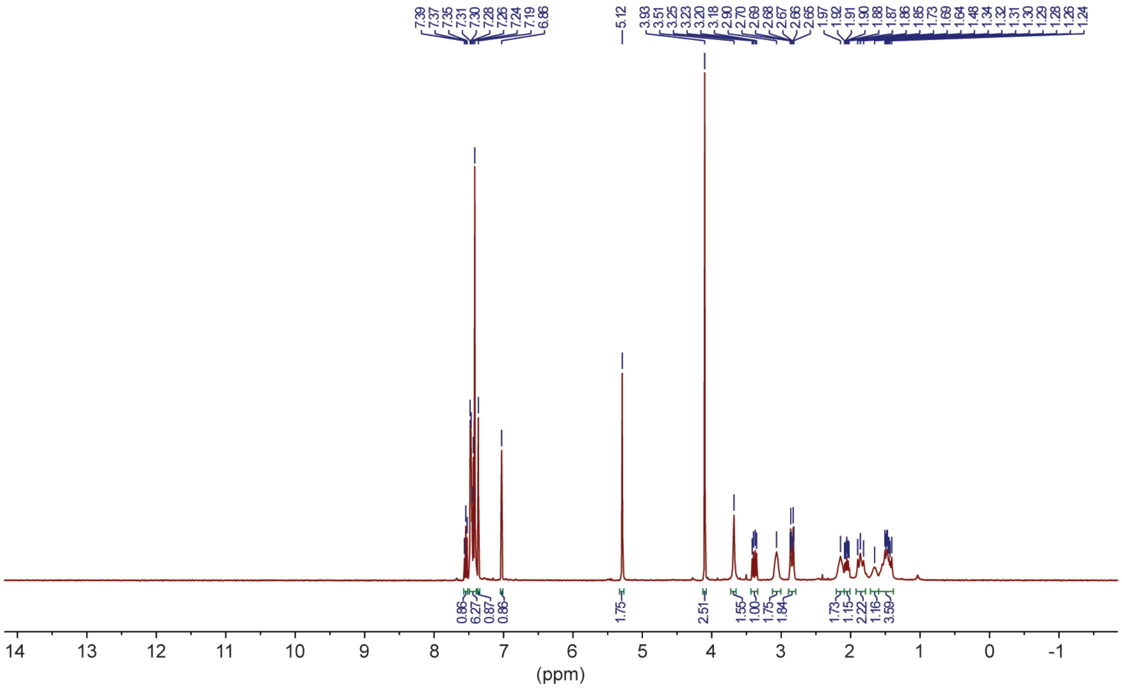

Fig. S73: ^{1}H NMR spectrum for compound **8t** in $CDCl_3$ (400 MHz).

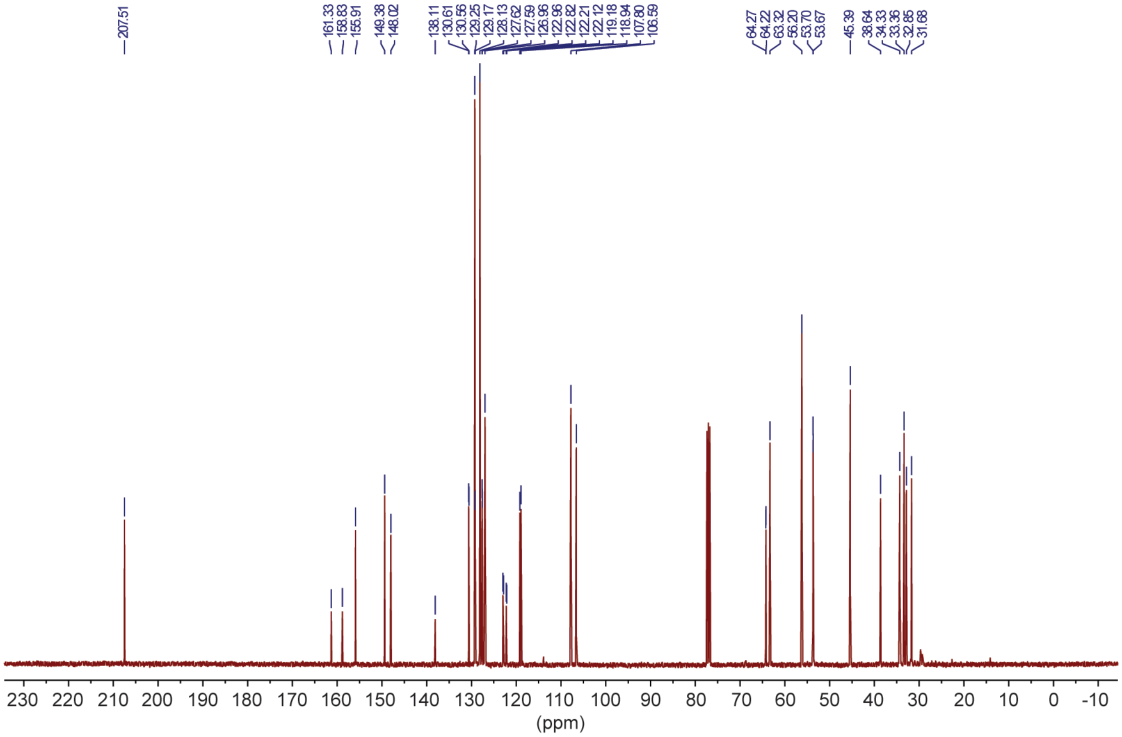

Fig. S74: ^{13}C NMR spectrum for compound **8t** in $CDCl_3$ (100 MHz).

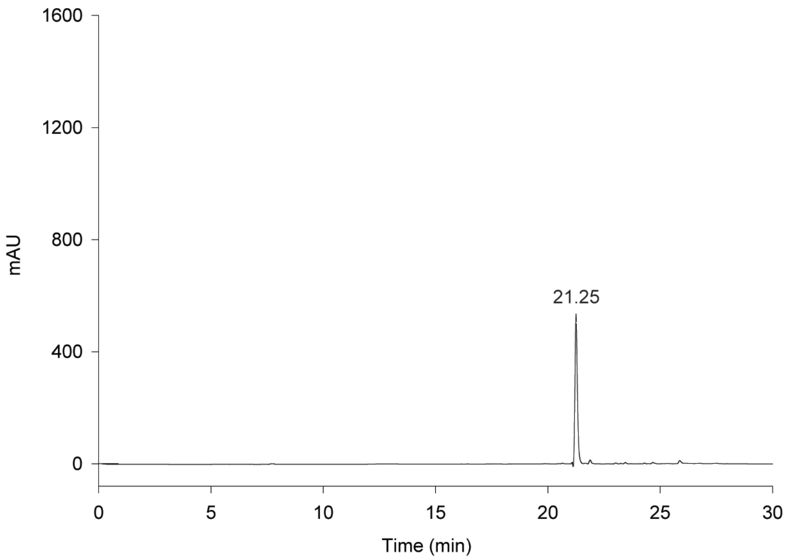

Fig. S75: HPLC trace for compound **8t**. R_t = 21.25 min.

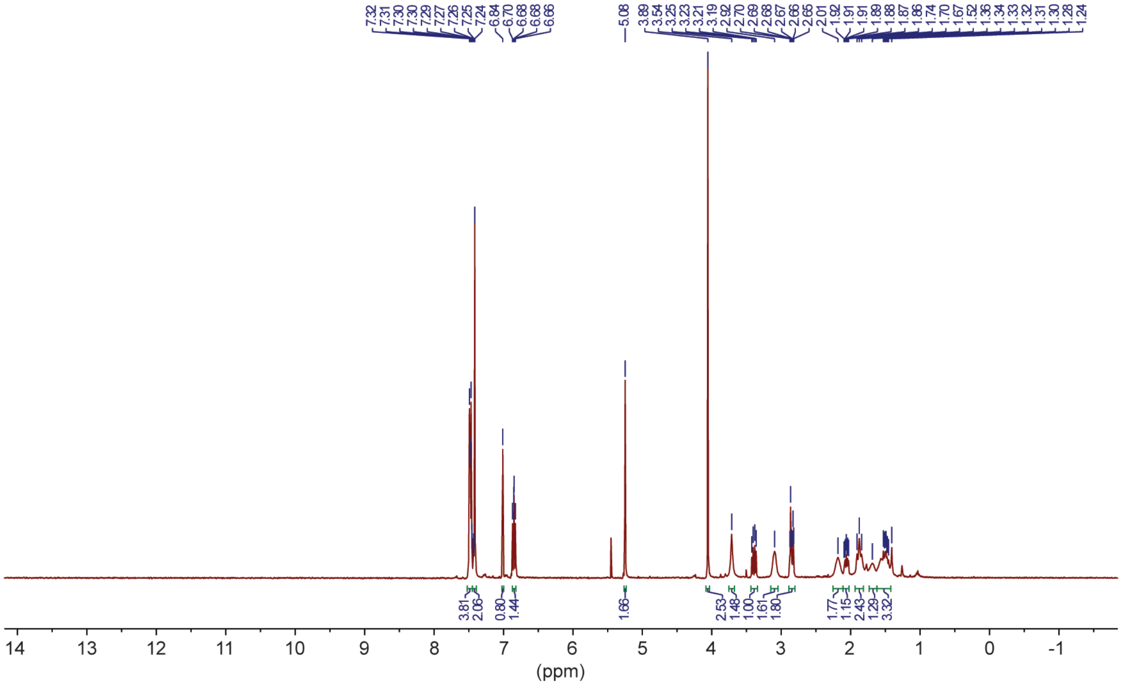

Fig. S76: ^{1}H NMR spectrum for compound **8u** in $CDCl_3$ (400 MHz).

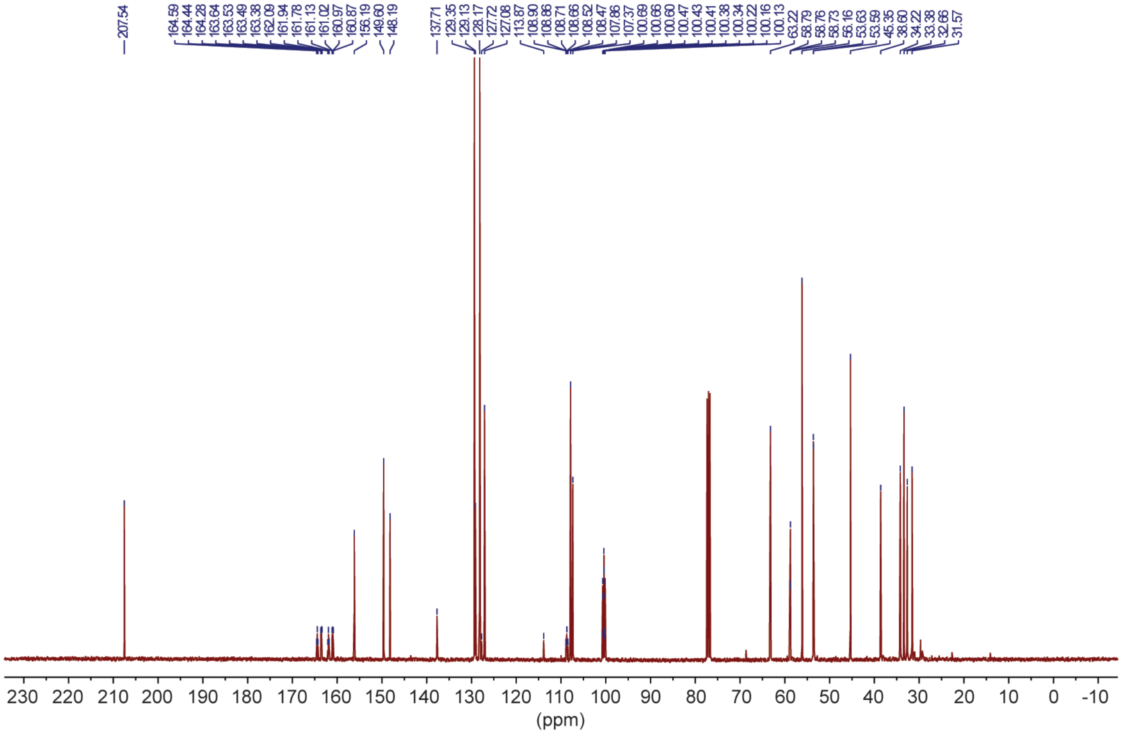

Fig. S77: ^{13}C NMR spectrum for compound **8u** in $CDCl_3$ (100 MHz).

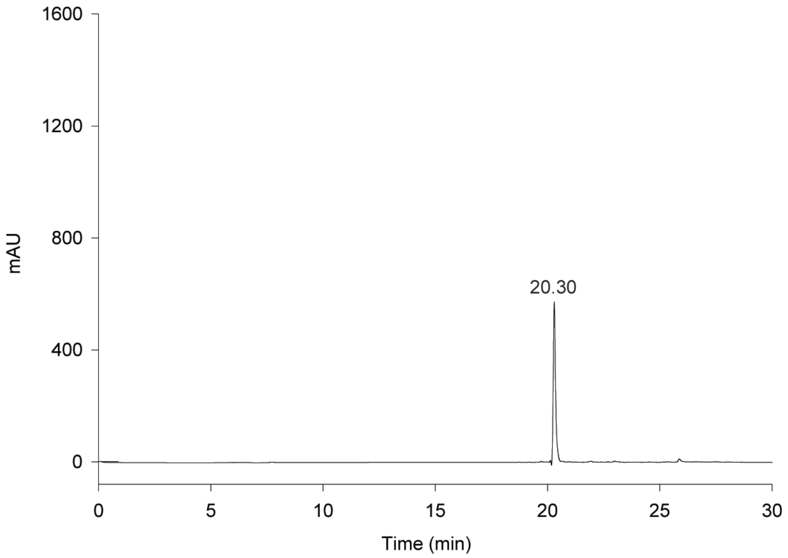

Fig. S78: HPLC trace for compound **8u**. R_t = 20.30 min.

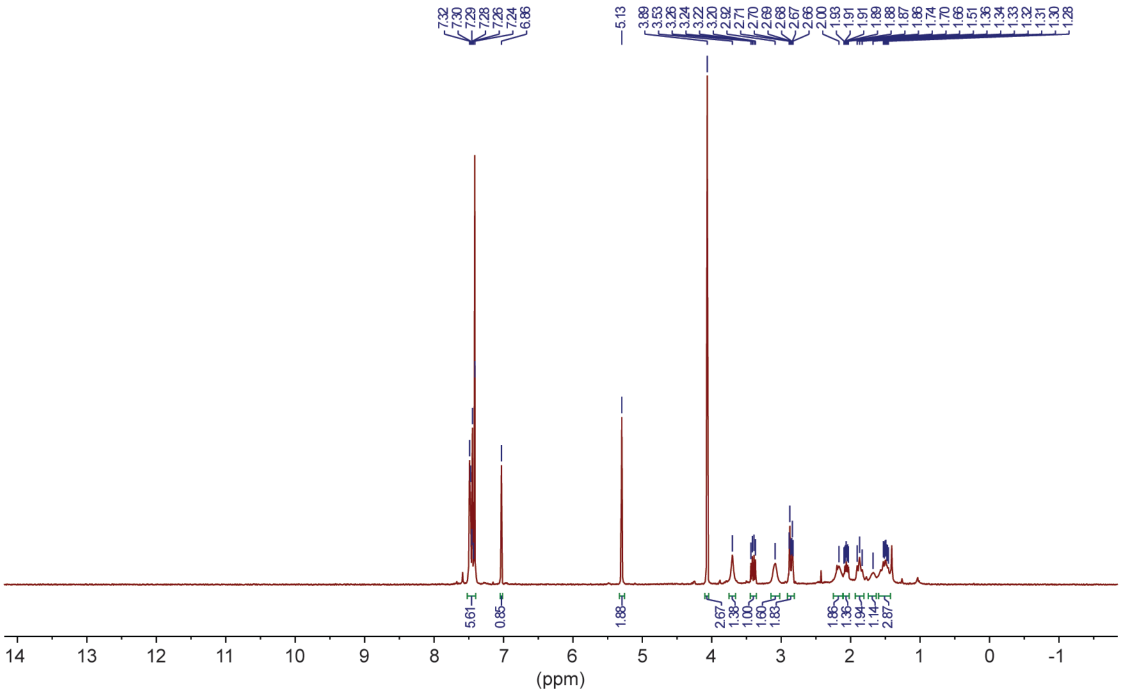

Fig. S79: ^{1}H NMR spectrum for compound **8v** in $CDCl_3$ (400 MHz).

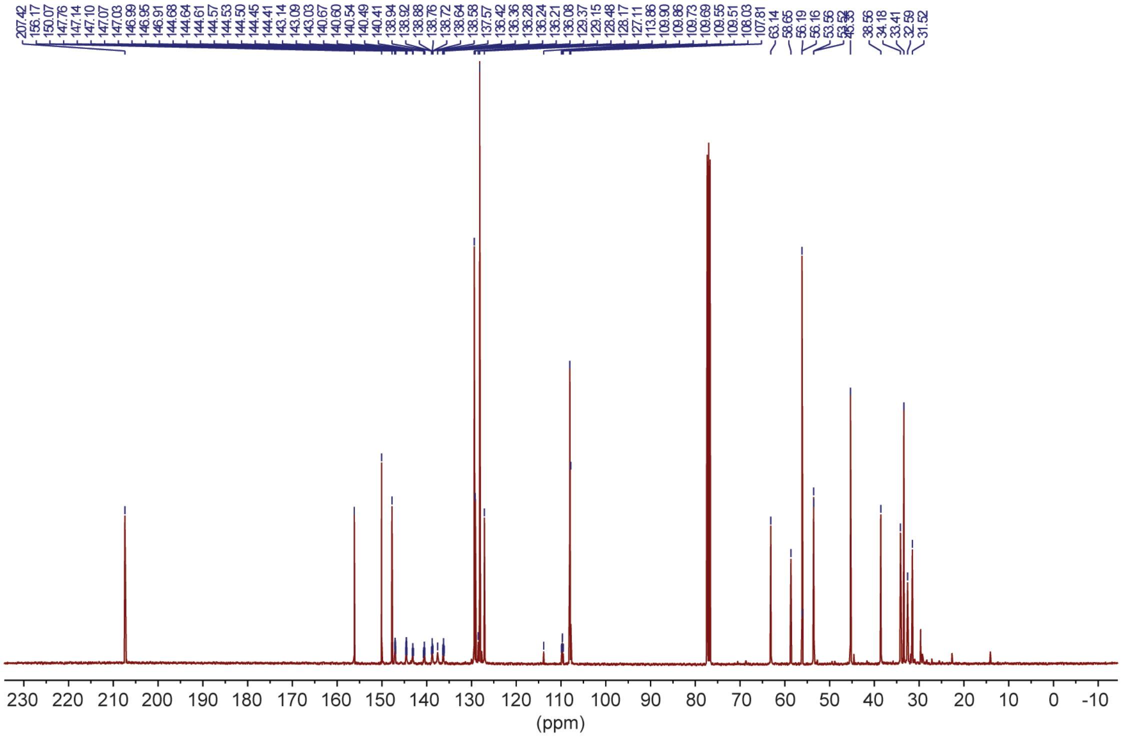

Fig. S80: ^{13}C NMR spectrum for compound **8v** in $CDCl_3$ (100 MHz).

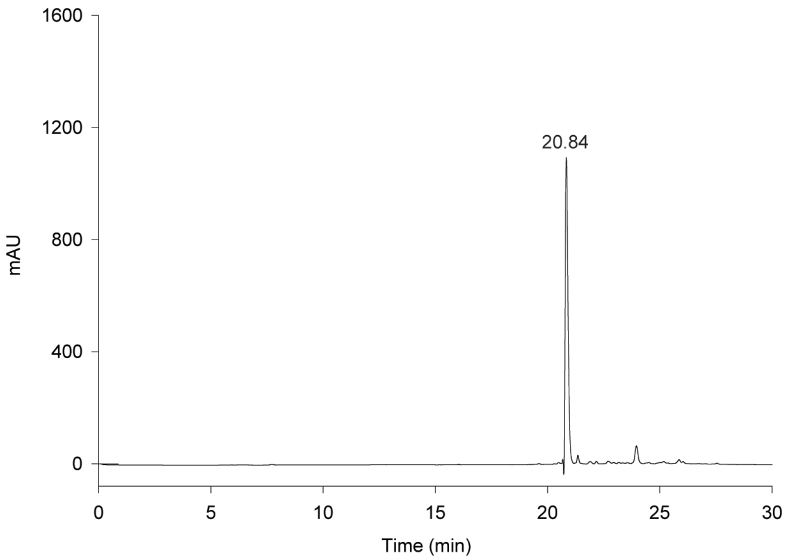

Fig. S81: HPLC trace for compound **8v**. R_t = 20.84 min.

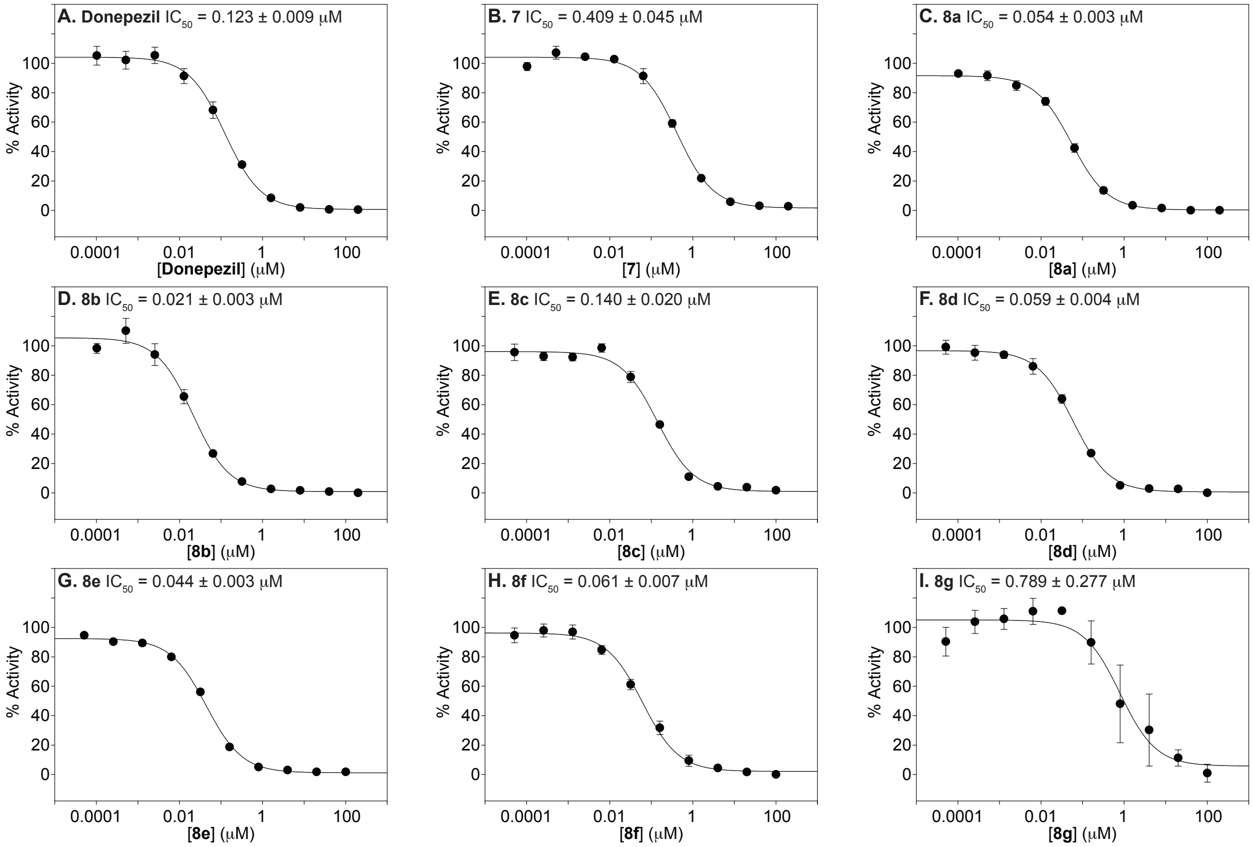

Fig. S82: IC_{50} curves for the inhibition of *Ee*AChE by donepezil and its analogues **7-8g**.

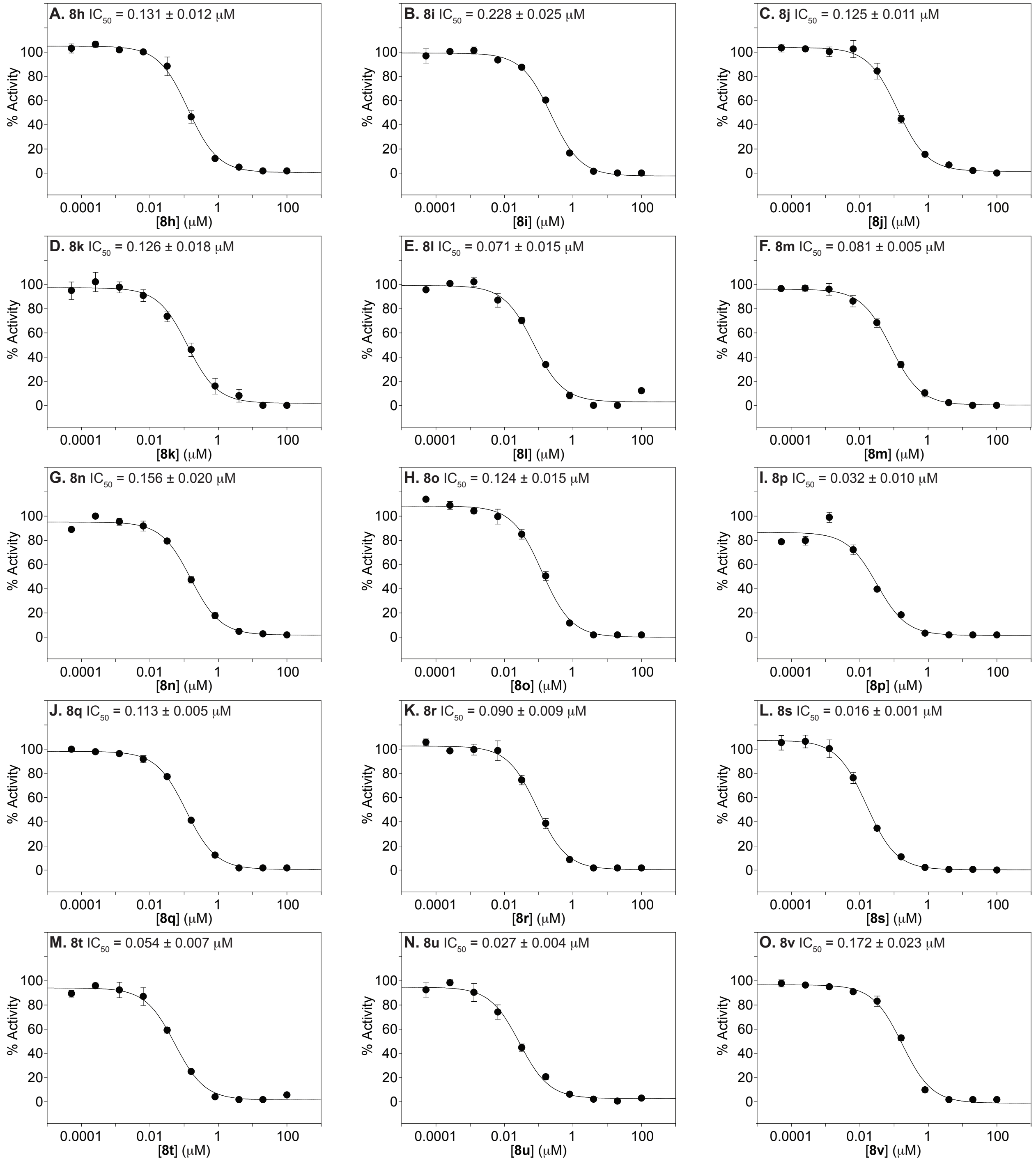

Fig. S83: IC_{50} curves for the inhibition of *Ee*AChE by donepezil analogues **8h-v**.

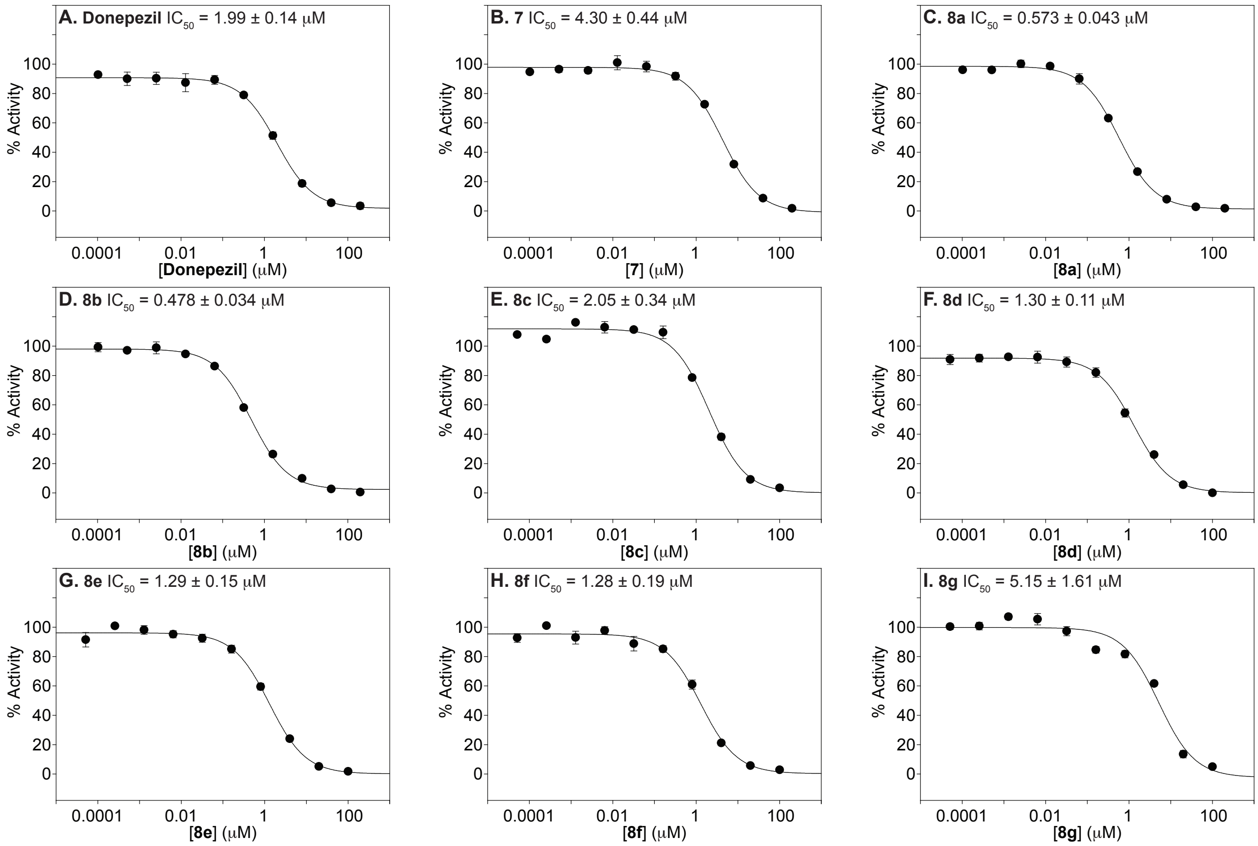

Fig. S84: IC_{50} curves for the inhibition of *Ef*BChE by donepezil and its analogues **7-8g**.

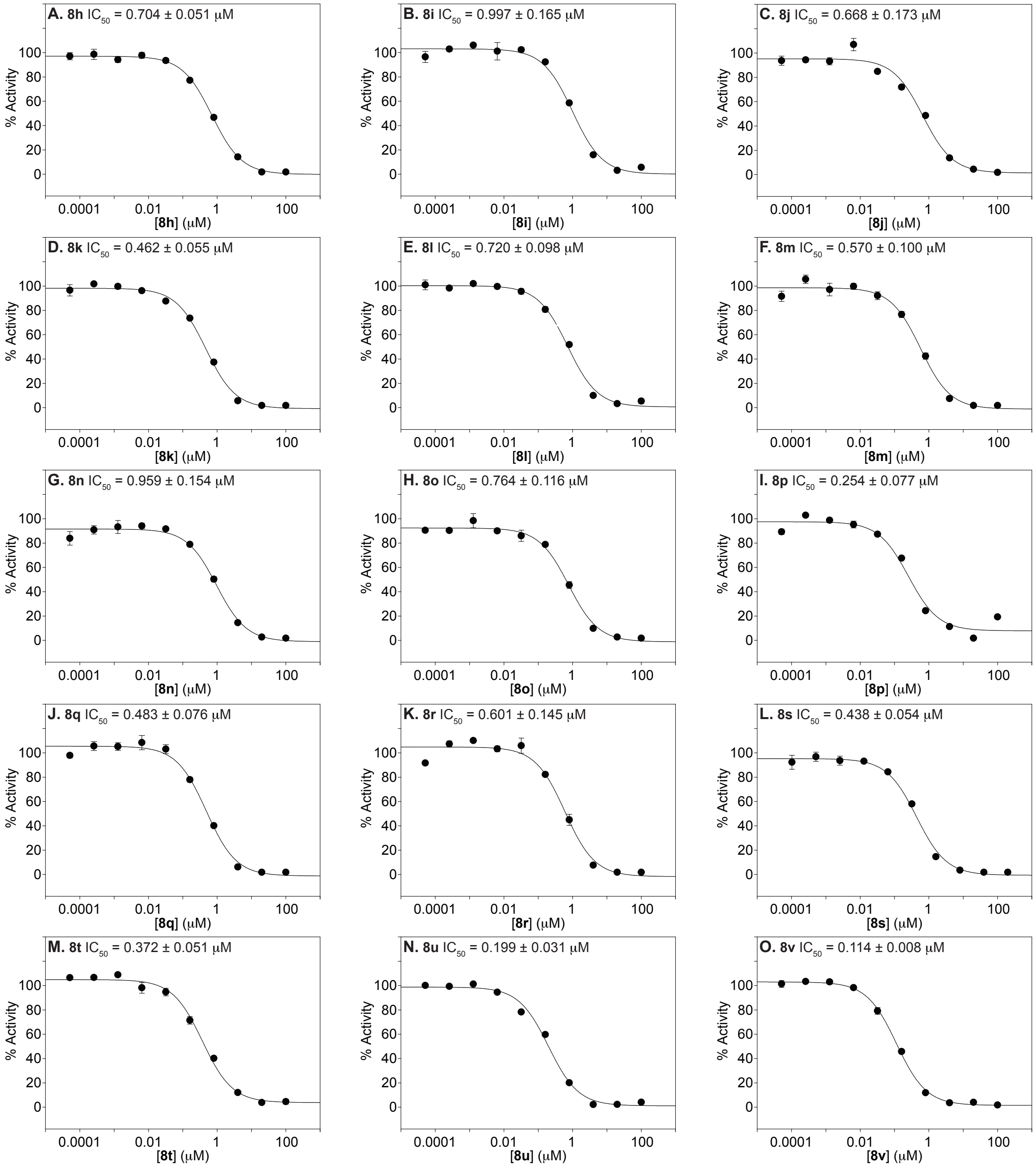

Fig. S85: IC_{50} curves for the inhibition of *Ef*BChE by donepezil analogues **8h-v**.

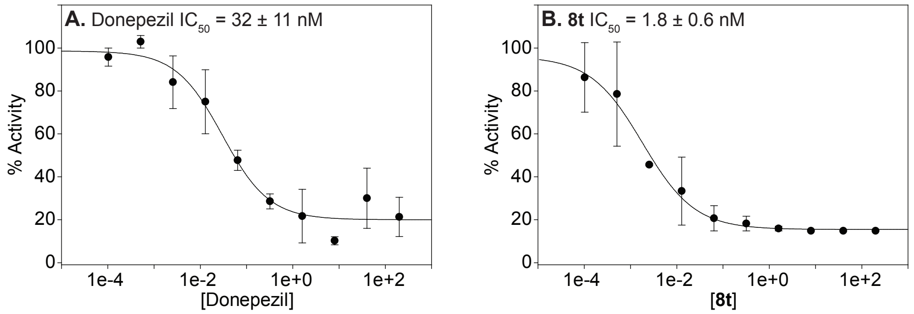

Fig. S86: IC_{50} curves for the inhibition of *Hs*AChE by donepezil and its analogue **8t**.

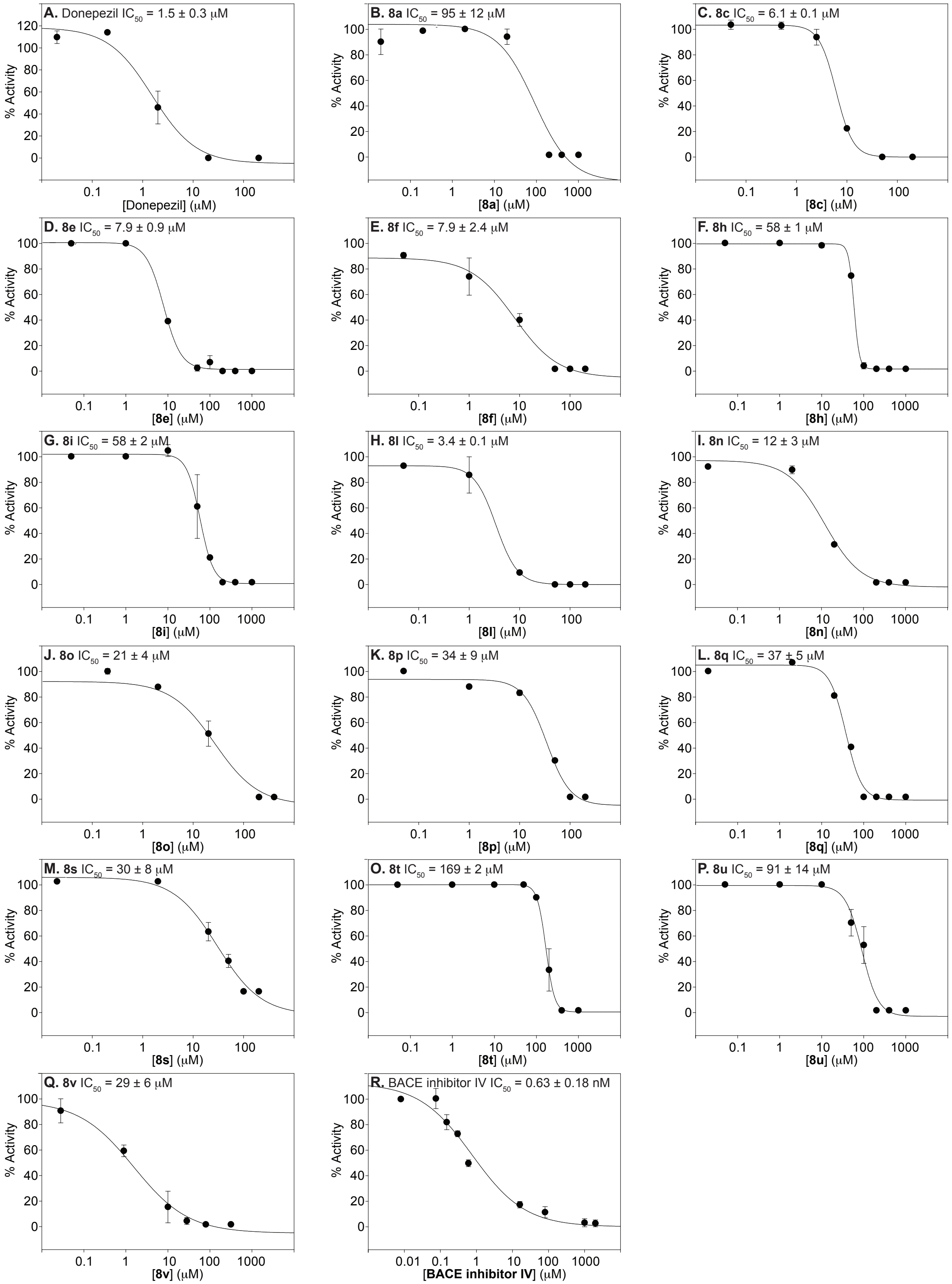

Fig. S87: IC_{50} curves for **A.** donepezil and **B-Q.** its analogues, as well as **R.** for BACE inhibitor IV.

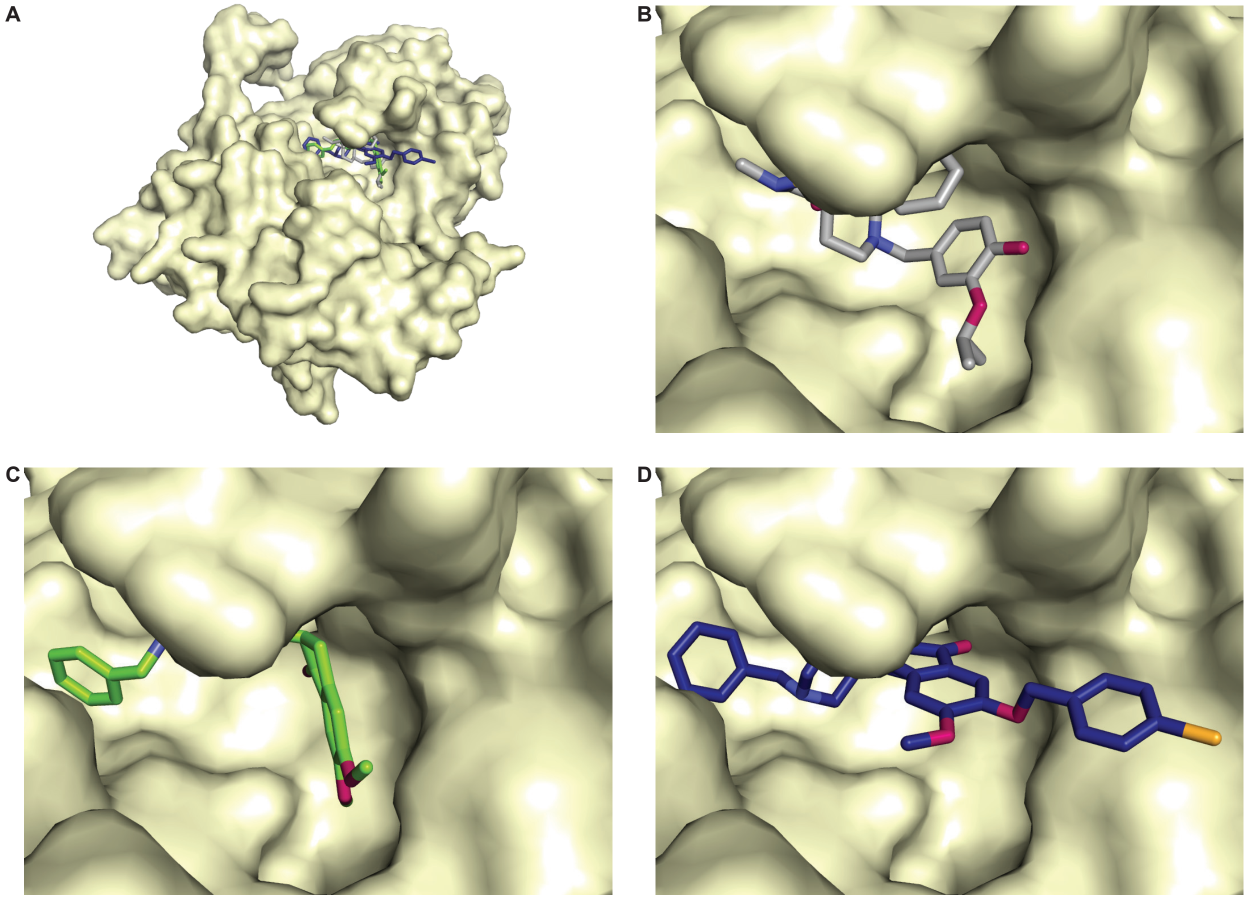

Fig. S88: Molecular docking showing the overlay of donepezil (green) and compound **8l** (navy blue) with the known BACE1 inhibitor (gray) crystallized with BACE1 (PDB# 4FM7) shown as surface representations. Panel **A** shows the three compounds in the active site of BACE1. Panels **B-D** show the zoomed-in view of the known inhibitor (**B**), donepezil (**C**), and compound **8l** (**D**). *Note*: This is the exact same figure as Fig. 1 in the main text to help visualize the space available to BACE1 inhibitor binding.
